# Highly recyclable and efficient electrocatalytic synthesis of adipic acid catalyzed by ultra-alkali-resistant [Ni_36_] nanocages in MOF

**DOI:** 10.1093/nsr/nwag427

**Published:** 2026-07-11

**Authors:** Ze-Long Liang, Rui Li, Zhen-Ting Wang, Jia-Can Lv, Xin-Min Ai, Qi Li, Ying-Ying Yu, Yang-Xi Wang, Fang-Ting Zhu, Qi-Fan Wang, Hang Xu, Bin Zhao

**Affiliations:** Frontiers Science Center for New Organic Matter, Key Laboratory of Advanced Energy Materials Chemistry (MOE), College of Chemistry, Nankai University, Tianjin 300071, China; School of Medicine, Nankai University, Tianjin 300071, China; Frontiers Science Center for New Organic Matter, Key Laboratory of Advanced Energy Materials Chemistry (MOE), College of Chemistry, Nankai University, Tianjin 300071, China; Frontiers Science Center for New Organic Matter, Key Laboratory of Advanced Energy Materials Chemistry (MOE), College of Chemistry, Nankai University, Tianjin 300071, China; Frontiers Science Center for New Organic Matter, Key Laboratory of Advanced Energy Materials Chemistry (MOE), College of Chemistry, Nankai University, Tianjin 300071, China; Frontiers Science Center for New Organic Matter, Key Laboratory of Advanced Energy Materials Chemistry (MOE), College of Chemistry, Nankai University, Tianjin 300071, China; Frontiers Science Center for New Organic Matter, Key Laboratory of Advanced Energy Materials Chemistry (MOE), College of Chemistry, Nankai University, Tianjin 300071, China; Frontiers Science Center for New Organic Matter, Key Laboratory of Advanced Energy Materials Chemistry (MOE), College of Chemistry, Nankai University, Tianjin 300071, China; Frontiers Science Center for New Organic Matter, Key Laboratory of Advanced Energy Materials Chemistry (MOE), College of Chemistry, Nankai University, Tianjin 300071, China; Frontiers Science Center for New Organic Matter, Key Laboratory of Advanced Energy Materials Chemistry (MOE), College of Chemistry, Nankai University, Tianjin 300071, China; Frontiers Science Center for New Organic Matter, Key Laboratory of Advanced Energy Materials Chemistry (MOE), College of Chemistry, Nankai University, Tianjin 300071, China; Frontiers Science Center for New Organic Matter, Key Laboratory of Advanced Energy Materials Chemistry (MOE), College of Chemistry, Nankai University, Tianjin 300071, China; Ganjiang Innovation Academy, Jiangxi Institute of Rare Earths, Chinese Academy of Sciences, Ganzhou 341000, China

**Keywords:** electrocatalysis, adipic acid, metal-organic frameworks, alkali resistance, high-nuclearity nanocages

## Abstract

As a pivotal industrial chemical, adipic acid (AA) is synthesizable through the electrocatalytic oxidation of cyclohexanol under ambient conditions, utilizing electrons as a clean oxidant. This approach circumvents the energy-intensive and environmentally detrimental nature of conventional petrochemical processes, thereby offering a sustainable alternative for AA production. However, existing electrocatalysts are often plagued by instability in alkaline media, and their structure–activity relationships remain poorly understood. Here, we report a novel [Ni_36_] nanocage-based metal-organic framework (**[Ni_9_]-MOF**) synthesized via a bottom-up strategy from discrete **[Ni_9_]-clusters**, exhibiting excellent stability under both strong alkaline (8 M NaOH) and acidic (1 M HCl) conditions. **[Ni_9_]-MOF** demonstrates superior AA production (0.1239 mmol h^−1^ cm^−2^ at 1.61 V), and maintains this high activity over 25 cycles with negligible loss, surpassing all prior high-performance catalysts in durability. Mechanistic studies reveal a three-step oxidation pathway via cyclohexanone, 2-hydroxycyclohexanone and 6-hydroxyadipic acid. Density functional theory calculations indicate the MOF framework enhances the charge density at the active **[Ni_9_]** site, accelerating reaction kinetics. This work not only provides a rational design strategy for constructing highly stable MOFs, but also presents the first MOF-based electrocatalyst for AA synthesis, opening new avenues for sustainable chemical production under mild conditions.

## Teaser

This work offers a rational design strategy for robust MOFs and presents the first MOF electrocatalyst for adipic acid synthesis, enabling sustainable chemical production under mild conditions.

## INTRODUCTION

Adipic acid (AA), a key industrial dicarboxylic acid widely used in nylon-66 [1–3], polyurethanes [4–6] and plasticizers [7,8], is projected to reach a global market value of $6.56 billion by 2030 [9–11]. Currently, over 95% of worldwide AA production relies on a multistep petrochemical process involving energy-intensive cyclohexane oxidation (125°C, 8–15 atm O_2_) followed by nitric acid treatment (50%–65%), which generates ∼3 tons of N_2_O per 10 tons of AA, along with other NO*_x_* emissions [12–14]. This conventional method suffers from low atom economy (∼10% yield), dependence on fossil feedstocks and substantial environmental drawbacks [15]. Therefore, there is an urgent need to develop a green, mild and cost-effective method for synthesizing AA. Recently, the electrocatalytic oxidation of KA oil (a mixture of cyclohexanol and cyclohexanone) to AA has emerged as a sustainable route. This approach employs electrons as clean oxidants under ambient conditions, circumventing the environmental hazards [16,17]. Since Petrosyan’s pioneering work (47% AA yield from cyclohexanol) [18], advancements in nickel-based catalysts (e.g. Ni(OH)_2_-SDS [19] and Cu-doped Ni(OH)_2_ [17]) have achieved Faradaic efficiencies (FEs) exceeding 85%. However, the structure–activity relationship of oxide-based catalysts remains poorly understood, limiting both industrial scalability and the rational design of improved catalysts. Furthermore, the electrocatalytic synthesis of AA generally requires the participation of hydroxide ions in strong alkaline electrolytes, where a harsh alkaline environment can cause the decomposition and deactivation of catalysts, leading to reduced catalytic performance in stability and recyclability [17,19]. Therefore, developing novel catalysts with high stability, high efficiency and tunable structures is urgently needed to advance this field.

As a novel porous material, metal-organic frameworks (MOFs) have attracted significant attention in electrocatalysis owing to their high porosity and tunable structures [20–22]. Among these, cluster-based MOFs exhibit particular promise for electrocatalysis, demonstrating both excellent stability in alkaline electrolytes and abundant catalytically active metal sites that enable highly electrocatalytic performance [23–28]. Our group reported several notable examples including [Zn_5_]- [29], [Hf_13_]- [30] and [Ln_60_] [31]-based MOFs, which exhibited robust acid–base stability, enabling potential application in harsh alkaline environment. Furthermore, the established performance of Ni-based catalysts in AA production suggests that Ni-based MOFs may represent ideal candidates for this transformation [6,32–37]. The inherent structural versatility of MOFs enables systematic exploration of structure–activity relationships, facilitating the rational design of high-performance electrocatalysts for AA synthesis. Furthermore, Ni-based catalysts exhibit excellent hydrogen evolution reaction (HER) performance in previous results [38–40]. Thus, Ni-clusters within MOFs may simultaneously realize electrochemical AA synthesis and green hydrogen production, thereby significantly improving overall energy utilization efficiency. Nevertheless, despite these advantageous characteristics, no MOF-based catalysts have been reported to date for electrocatalytic AA synthesis.

In this contribution, a novel 2D framework {[Ni_9_(btz)_12_(3-pba)_6_]·16H_2_O·8 *N,N*-dimethylformamide (DMF)·4CH_3_OH}*_n_*  **([Ni_9_]-MOF**, Hbtz = benzotriazole, 3-Hpba = 3-(3-pyridinyl)benzoic acid) was synthesized via the bottom-up synthesis strategy from a discrete **[Ni_9_]-cluster** ({[Ni_9_(btz)_12_(NO_3_)_6_(DMA)_6_]·3 *N,N*-dimethylacetamide (DMA)}*_n_*), exhibiting [Ni_36_] cages with the size of 14.4 × 14.4 × 10.0 Å^3^. Remarkably, **[Ni_9_]-MOF** demonstrated excellent stability in both strong alkaline (8 M NaOH) and acid (1 M HCl) aqueous solutions, and such high acid–base stability is rather rare among reported MOFs. The excellent alkaline stability of **[Ni_9_]-MOF** originated from its pronounced hydrophobicity, confirmed by contact angle measurements. Electrocatalytic studies demonstrated that **[Ni_9_]-MOF** achieved an AA production rate of 0.1239 mmol h^−1^ cm^−2^ at 1.61 V, significantly outperforming that of discrete **[Ni_9_]-cluster** (0.0749 mmol h^−1^ cm^−2^) and ranking among the highest values reported to date. The robust **[Ni_9_]-MOF** maintained its catalytic activity over 25 cycles without significant degradation, surpassing all previously reported high-performance catalysts. When integrated into a membrane electrode assembly (MEA), the production rate increased to 1.94 mmol h^−1^ with concurrent 8.39 mmol h^−1^ H_2_ generation, suggesting its potential for practical applications. Mechanistic analysis revealed a three-step electrooxidation pathway for cyclohexanol: initial conversion to cyclohexanone, followed by sequential oxidation to 2-hydroxycyclohexanone and 6-hydroxyadipic acid, ultimately yielding AA. Density functional theory (DFT) analysis further revealed that the [Ni_9_] active sites in **[Ni_9_]-MOF** exhibited enhanced electron density during catalysis (increasing from 0.157 to 0.164 e^−^ per site) than those in the discrete **[Ni_9_]-cluster**. This electronic enhancement facilitated faster oxidation kinetics, leading to superior catalytic performance. This work not only presents a facile strategy for designing highly stable MOFs with polynuclear clusters but also reports the first MOF-based catalyst for the electrosynthesis of AA.

## RESULTS AND DISCUSSION

### Crystal synthesis and characterization

To accurately construct MOFs with high-nuclearity clusters, our group previously proposed a bottom-up synthesis strategy [23], which established a geometric methodology for designing specific network topologies. This approach utilized discrete **[Ni_9_]-cluster** as a secondary building unit, followed by stepwise substitution of coordinated NO_3_^−^ and DMA with organic linkers, ultimately forming **[Ni_72_]** nanocages within MOF. Meanwhile, 2D catalysts demonstrate advantages in the electrocatalytic synthesis of AA due to the following reason [19,27]: 2D layered structures expose a high density of active sites and enhance interfacial contact between reactants and the catalyst surface, thereby enhancing reaction efficiency. Based on the above results, **[Ni_9_]-cluster** was initially synthesized as a discrete building block (Fig. 1a), and the coordinated NO_3_^−^ and DMA were replaced by carboxyl groups and pyridinic nitrogen atoms from (3-pyridinyl)benzoic acid (3-Hpba), forming a 2D layered framework **[Ni_9_]-MOF**. Single-crystal X-ray diffraction (SXRD) analyses revealed that **[Ni_9_]-MOF** crystallized in the hexagonal system with the *P*6_3_ space group (Table S1, CCDC (Cambridge Crystallographic Data Centre): 2492643). In detail, the 2D framework was assembled by diamond **[Ni_36_]** cages with the size of 14.4 × 14.4 × 10.0 Å^3^ (Fig. 1c), which were constructed by 4 **[Ni_9_]-clusters** through 10 ligands. From the topological point of view, a **[Ni_9_]-cluster** coordinated by 12 ligands, where two adjacent clusters are bridged by dual ligands (Fig. 1b). This connectivity pattern assigns each **[Ni_9_]** node as a 6-connected unit, ultimately yielding a 2,6-connected *hxl*-type topology (Fig. 1d).

**Figure 1.**
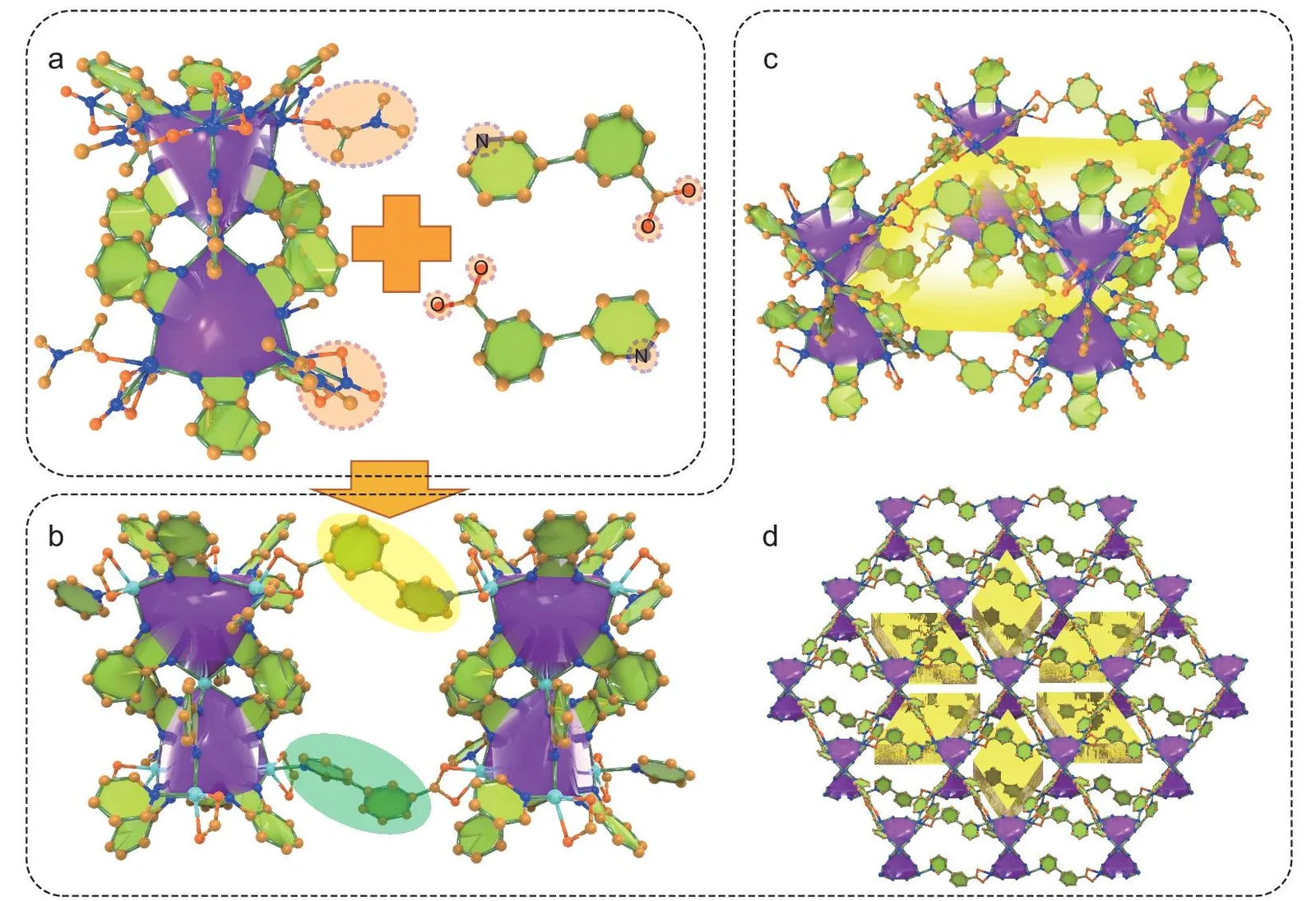
Structure diagram of **[Ni_9_]-cluster** and **[Ni_9_]-MOF**. Assembly process of **[Ni_9_]-MOF** through the bottom-up synthesis strategy. (a) Structure diagram of **[Ni_9_]-cluster** and (3-pyridinyl)benzoic acid. (b) Connection diagram of **[Ni_9_]-MOF**. (c) The structure of [Ni_36_] nanocages. (d) 3D framework of **[Ni_9_]-MOF** with a 2,6-connected ***hxl*** topology.

Scanning electron microscopy confirmed the hexagonal sheet-like structure of **[Ni_9_]-MOF**. Energy dispersive X-ray spectroscopy confirmed the uniform distribution of nickel within the crystalline framework (Fig. S1). Powder X-ray diffraction (PXRD) and SXRD analyses revealed the long-range ordered structure of the framework (Figs S2 and S3 and Tables S1 and S2). The nitrogen adsorption isotherm measurements of **[Ni_9_]-MOF** demonstrated a high N_2_ adsorption capacity of 214 m^3^ g^−1^, along with a Brunauer–Emmett–Teller (BET) surface area of 833 m^2^ g^−1^ (Fig. S4), consistent with its porous structure. Pore volume analysis via DFT determined the pore size of **[Ni_9_]-MOF** to be 0.44 nm (Fig. S5), aligning with the interlayer spacing of its crystalline structure (4.3 Å) (Fig. S6). X-ray photoelectron spectroscopy (XPS) analysis revealed that the oxidation state of nickel in **[Ni_9_]-MOF** was +2, as evidenced by the characteristic 2*p*_3/2_ and Ni 2*p*_1/2_ signals at 856.3 and 873.9 eV, respectively, along with their corresponding satellite features at 862.4 and 880.1 eV. The above characterization data definitively demonstrated the successful synthesis of **[Ni_9_]-MOF**.

The phase purity of the as-synthesized **[Ni_9_]-MOF** was characterized by PXRD. The experimental PXRD pattern matched well with the simulated one, confirming the high phase purity of the samples. Thermogravimetric analysis and solvent stability tests revealed that **[Ni_9_]-MOF** could keep its intact framework up to 383°C (Fig. S10) or immersed in various solvents (Fig. S11), suggesting its high thermostability and solvent stability. Subsequent tests were conducted to evaluate the acid–base stability of **[Ni_9_]-MOF** in aqueous environments. PXRD patterns demonstrated its chemical stability over 24 h across a broad pH range (1–14) (Fig. S12). The corresponding PXRD patterns showed that **[Ni_9_]-cluster** retained integrity from pH 2–13 (Fig. S13). Notably, **[Ni_9_]-MOF** maintained structural integrity even in 8 M NaOH and 12 M KOH (Fig. 2a and Fig. S14), exhibiting strong alkaline resistance. The corresponding N_2_ adsorption capacity and BET surface area are 198 m^3^ g^−1^ and 757 m^2^ g^−1^ after the base stability tests, further confirming its high alkaline stability (Fig. S15). In comparison, upon exposure to increasing concentrations of hydrochloric acid (HCl), **[Ni_9_]-MOF** gradually underwent structural degradation with the concentration of HCl over 1 M (Fig. S16). Contact angle measurements demonstrated that **[Ni_9_]-MOF** exhibited significantly higher hydrophobicity in alkaline solutions than in acidic media (Figs S17 and S18), explaining its enhanced stability under basic conditions. This differential wetting behavior explained **[Ni_9_]-MOF’s** pronounced alkaline resistance versus limited acid tolerance. The high stability in alkaline conditions was further corroborated by SXRD data obtained from **[Ni_9_]-MOF** crystals immersed in 2 M NaOH (Table S3), demonstrating retained framework integrity under harsh basic treatment. As the concentration of the alkaline solution increased, the water contact angle of **[Ni_9_]-MOF** surface rose significantly from 129.05° (pH = 1) to 147.89° (in 8 M NaOH) (Fig. 2c and d, Figs S17 and S18). Figure 2b summarizes the pH-dependent aqueous chemical stability ranges of several typical MOFs [41–48]. **[Ni_9_]-MOF** demonstrated high chemical stability across a broader pH range compared to these MOFs, with particularly notable robustness in alkaline environments. A significant increase in hydrophobicity under strongly alkaline conditions further confirmed the high alkali resistance of **[Ni_9_]-MOF**. Previous studies attributed this high stability to two primary mechanisms [49, 41, 50]: (i) twelve benzotriazole ligands construct a rigid framework of the Ni_9_ cluster in **[Ni_9_]-MOF**. This framework geometrically locks the coordination configuration of carboxylate groups and inhibits ligand rearrangement as well as framework hydrolysis at metal centers. (ii) **[Ni_9_]-MOF** framework exhibits favorable hydrophobicity, which effectively blocks the erosion of alkaline electrolyte and protects the internal skeleton structure. Thus, the 2D layered **[Ni_9_]-MOF** with high alkaline resistance serves as a promising durable platform for electrocatalytic applications, particularly in the electrochemical synthesis of AA.

**Figure 2.**
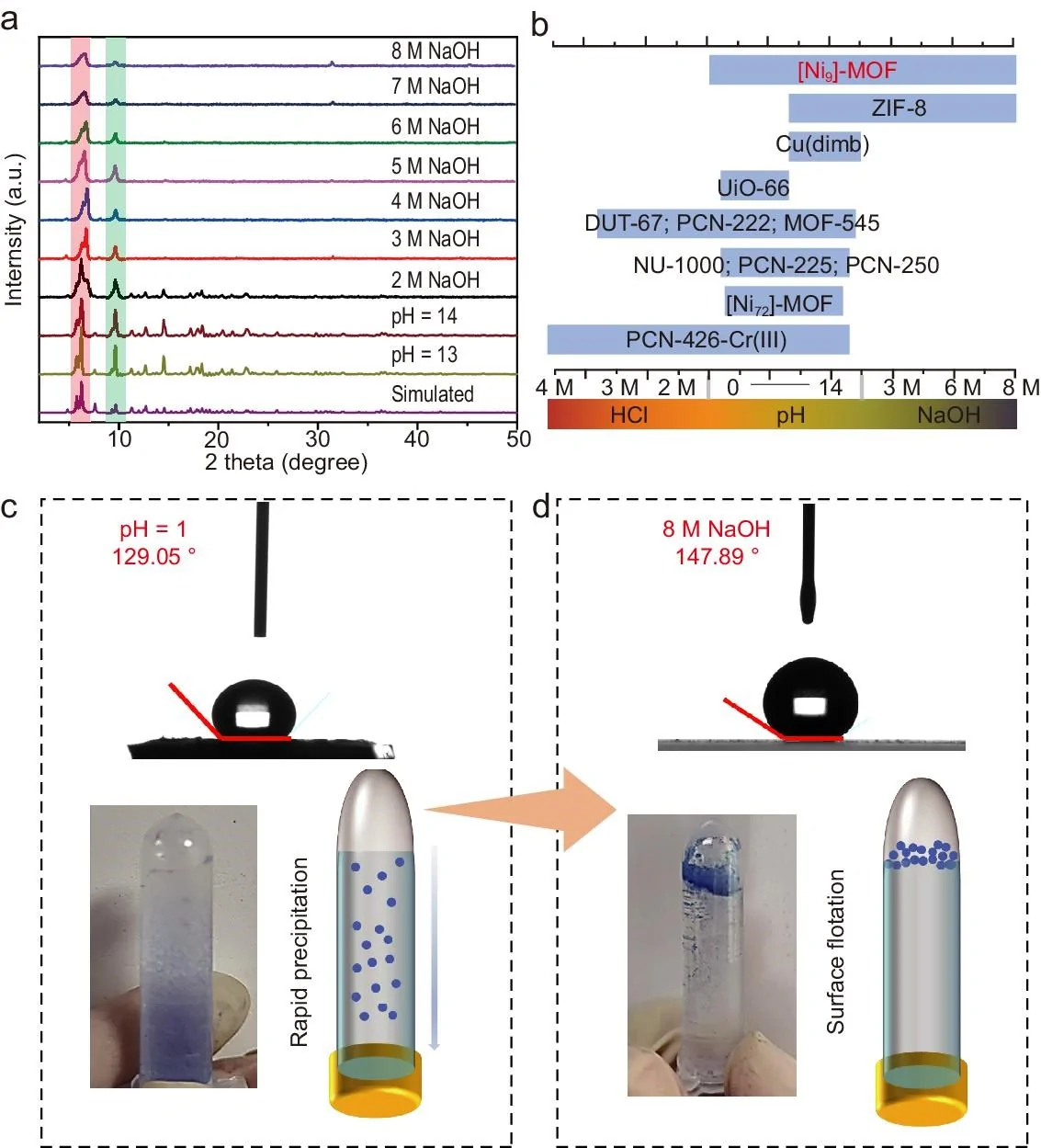
Stability assessment of **[Ni_9_]-MOF** revealed exceptional robustness under extreme conditions. (a) The PXRD spectra of **[Ni_9_]-MOF** sample immersed in a high-concentration aqueous NaOH solution for 24 h were analyzed. (b) The chemical (acid–base) stability of some representative MOFs based on literature data [41–48]. Complementary contact angle measurements showed hydrophobic character with pH = 1 solution (*θ* = 129.05°) (c) and 8 M NaOH solution (*θ* = 147.89°) (d), consistent with visual observations of intact crystals in corresponding immersion tests.

### Catalytic performance

The electrocatalytic performance of **[Ni_9_]-MOF** for AA synthesis was evaluated via cyclohexanol oxidation using a conventional three-electrode system in 0.5 M KOH electrolyte. Linear sweep voltammetry (LSV) was first employed to investigate the influence of cyclohexanol concentrations on catalytic performance. As evidenced in Fig. S19, the optimal electrocatalytic activity toward cyclohexanol oxidation was achieved at 0.1 M cyclohexanol concentration in 0.5 M KOH solution. Cyclic voltammetry (CV) was initially employed to investigate current responses and catalytic activity in a 0.5 M KOH electrolyte in the absence or presence of 0.1 M cyclohexanol (Fig. 3a). Upon the addition of cyclohexanol under the optimal concentration, a significant decrease in onset potential was observed, accompanied by enhanced current density, both results being attributed to the electrochemical oxidation of cyclohexanol. Notably, cyclohexanol underwent electrochemical oxidation at 1.61 V (vs reversible hydrogen electrode (RHE)) with a current density of 52.7 mA cm^−2^, representing a 240 mV reduction in overpotential compared to the oxygen evolution reaction (OER) potential (1.85 V) under identical conditions (Fig. S20). This significant decrease in required potential demonstrated the energetic efficiency of **[Ni_9_]-MOF**-catalyzed process. Meanwhile, the CV curve further revealed that **[Ni_9_]-MOF** achieved a higher current density than **[Ni_9_]-cluster** in 0.1 M cyclohexanol (Fig. S21). The electrosynthesis of AA using **[Ni_9_]-MOF** was evaluated in 0.5 M KOH containing 0.1 M cyclohexanol, and the corresponding oxidation products were analyzed post electrolysis by ^1^H nuclear magnetic resonance (^1^H NMR). Unless otherwise stated, all potentials are referenced to the RHE. As illustrated in Fig. 3b, **[Ni_9_]-MOF** demonstrated a statistically significant enhancement in AA production yield, exhibiting an increase in reaction rate from 0.0749 to 0.1239 mmol h^−1^ cm^−2^ and a rise in FE from 51.1% to 87.6% relative to the **[Ni_9_]-cluster** system at 1.61 V. The corresponding current density was 26.57 mA cm^−2^. This performance superiority persisted across the electrochemically relevant potential window of 1.41–1.81 V, suggesting structural advantages inherent to **[Ni_9_]-MOF** architecture in facilitating charge transfer and stabilizing reaction intermediates under operational conditions. The time-dependent product analysis (Fig. 3c) revealed rapid cyclohexanol consumption alongside a progressive buildup of cyclohexanone, the latter attaining a distinct maximum before gradual depletion. This kinetic profile is consistent with sequential oxidation: cyclohexanol underwent initial dehydrogenation to cyclohexanone, which is then further oxidized to AA via C–C bond cleavage. After 11.38 h electrolysis, **[Ni_9_]-MOF** achieved a 72.3% AA yield with 80.9% FE, surpassing most reported cyclohexanol/cyclohexanone electrooxidation catalysts (Table S4). The time-dependent ^1^H NMR spectroscopy (Fig. 3d) monitored the reaction progress, revealing continuous consumption of cyclohexanol along with the progressive formation of AA throughout the transformation. Systematic comparison of nickel foam, **[Ni_9_]-cluster, [Ni_9_]-MOF** and Ni(OH)_2_ revealed significantly different performances (Fig. 3b–e and Figs S24 and S25). After the reaction, bare nickel foam and Ni(OH)_2_ produced negligible AA yields of only 0.0134 and 0.0283 mmol h^−1^ cm^−2^, respectively, with correspondingly low FE. In contrast, both **[Ni_9_]-cluster** and **[Ni_9_]-MOF** exhibited significantly higher performance, achieving AA production of 0.0749 and 0.1239 mmol h^−1^ cm^−2^, respectively. These results indicated that **[Ni_9_]-MOF** electrocatalyst demonstrated markedly enhanced performance in AA synthesis compared to **[Ni_9_]-cluster**. Electrochemical cycling tests revealed that **[Ni_9_]-MOF** retained over 80% FE after 25 consecutive cycles (50 h) (Fig. 3f), confirming its high catalytic activity and operational durability. To elucidate the superior electrocatalytic performance of **[Ni_9_]-MOF** over **[Ni_9_]-cluster**, intrinsic activity was quantified through double-layer capacitance (*C*_dl_) measurements. Electrochemical surface area (ECSA) calculations revealed a 23.7% larger active area for **[Ni_9_]-MOF** (0.2507 mF cm^−2^) versus **[Ni_9_]-cluster** (0.2026 mF cm^−2^) (Figs S26–S29). This enhanced ECSA directly correlated with improved accessibility of catalytically active sites, confirming **[Ni_9_]-MOF's** superior intrinsic activity for AA electrosynthesis.

**Figure 3.**
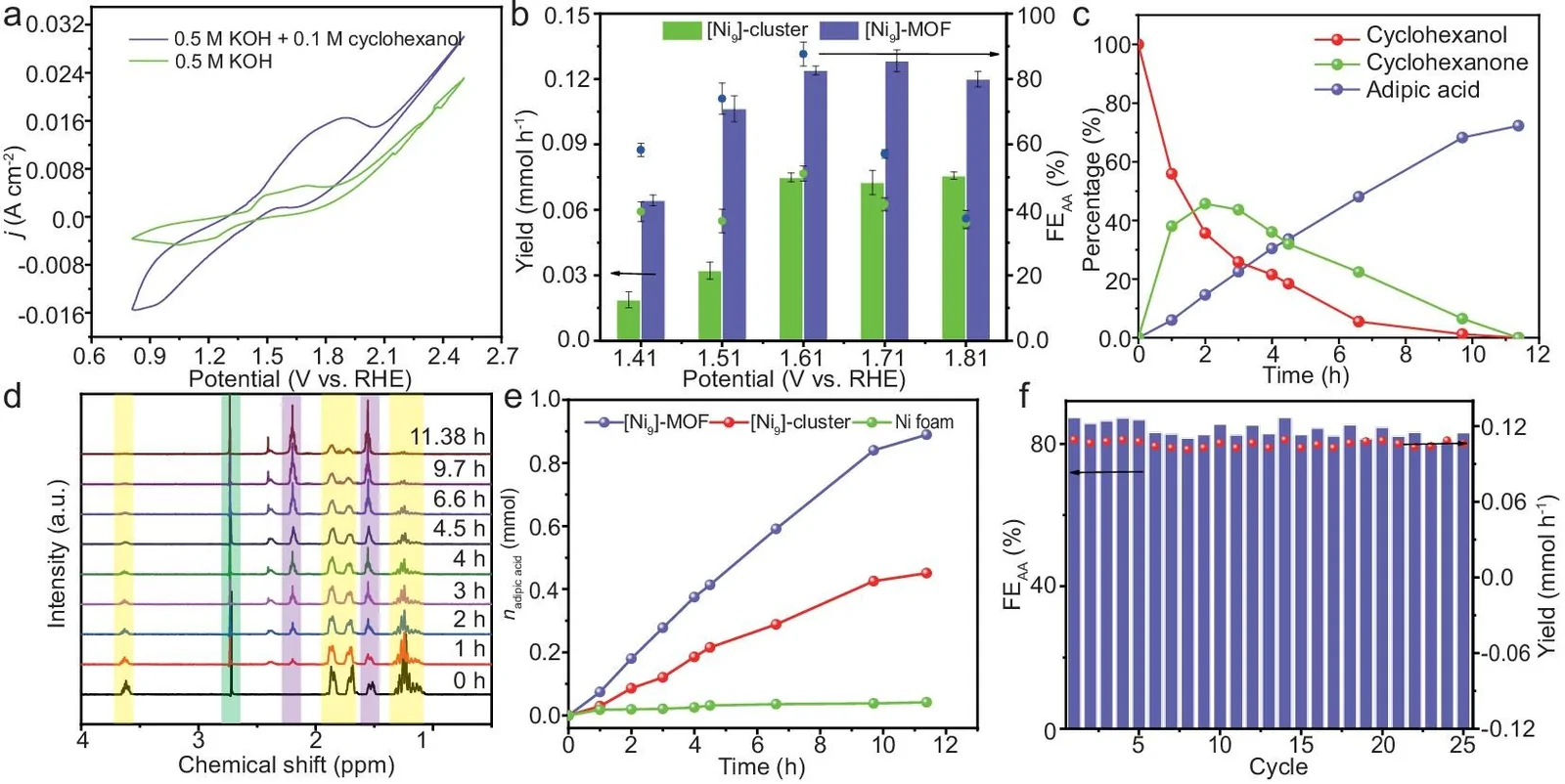
Electrocatalytic performance of materials. (a) CV curves of **[Ni_9_]-MOF** in 0.5 M KOH with and without 0.1 M cyclohexanol in Carbon paper. (b) FE and yield of electrosynthesis of AA using **[Ni_9_]-cluster** and **[Ni_9_]-MOF**. (c) Temporal variations in the concentrations of cyclohexanol, cyclohexanone and AA were systematically monitored during the electrolysis process. The content of cyclohexanone was measured by liquid chromatography. (d) The time-dependent ^1^H NMR spectra of the electrosynthesis of AA, with the positions of cyclohexanol, AA and the internal standard DMSO, labeled with yellow, purple and green, respectively. (e) Comparison of the production amounts of AA synthesized by electrosynthesis over time for Ni foam, **[Ni_9_]-cluster** and **[Ni_9_]-MOF**. (f) FE and synthesis rate of AA obtained from **[Ni_9_]-MOF** cycle for 25 times.

### Stability of catalyst

Structural stability is a critical parameter for evaluating the practical application of MOF-based electrocatalysts. PXRD analysis confirmed that **[Ni_9_]-MOF** still kept its original crystallinity, with diffraction peaks consistent with those observed prior to electrolysis (Fig. 4a). To determine whether the structural stability stems from framework robustness or surface passivation, the supernatant after stability testing was analyzed by NMR. The ^1^H NMR spectrum showed two very small peaks at 7.92 and 1.92 ppm (area ratio ∼1:1.28), but with intensity near the detection limit. The ^13^C NMR spectrum showed no discernible signals even after 512 scans (Fig. S30). No peaks corresponding to organic ligands were observed, indicating negligible ligand leakage. The NMR results further confirm the high stability of **[Ni_9_]-MOF**. XPS analyses of **[Ni_9_]-MOF** before and after the electrocatalysis demonstrated nearly identical results in both peak positions and line shapes (Fig. 4b and Figs S31–S34). In detail, the Ni 2*p*, N 1*s*, C 1*s* and O 1*s* spectra exhibited negligible changes. To further explore the electronic structure and coordination environment, X-ray absorption spectroscopy was employed. The Ni K-edge X-ray absorption near-edge structure (XANES) spectra indicated that the oxidation state of Ni in **[Ni_9_]-MOF** powder was still unchanged after the reaction (Fig. 4c). The results were supported by the Fourier-transform extended X-ray absorption fine structure (FT-EXAFS) analysis (Fig. 4d). Furthermore, the wavelet transform (WT)-EXAFS contour plots further confirmed the FT-EXAFS results (Fig. 4e and f). These results demonstrated the robust structural stability of **[Ni_9_]-MOF** with high performance in electrocatalytic cyclohexanol oxidation. On the other hand, to further explore the electrocatalytic universality of **[Ni_9_]-MOF**, several substrates were applied in electrocatalysis oxidation. As shown in Table 1, the oxidation products from substrates (cyclobutanol, cyclopentanol, cycloheptanol and cyclooctanol) show high yield rates: succinic acid (0.1353 mmol h^−1^ cm^−2^; Figs S35 and S36), glutaric acid (0.1176 mmol h^−1^ cm^−2^; Figs S37 and S38), heptanedioic acid (0.1121 mmol h^−1^ cm^−2^; Figs S39 and S40) and suberic acid (0.0793 mmol h^−1^ cm^−2^; Figs S41 and S42). Thus, **[Ni_9_]-MOF**, with its high stability and broad catalytic versatility, demonstrated promising potential for the electrocatalytic synthesis of AA.

**Figure 4.**
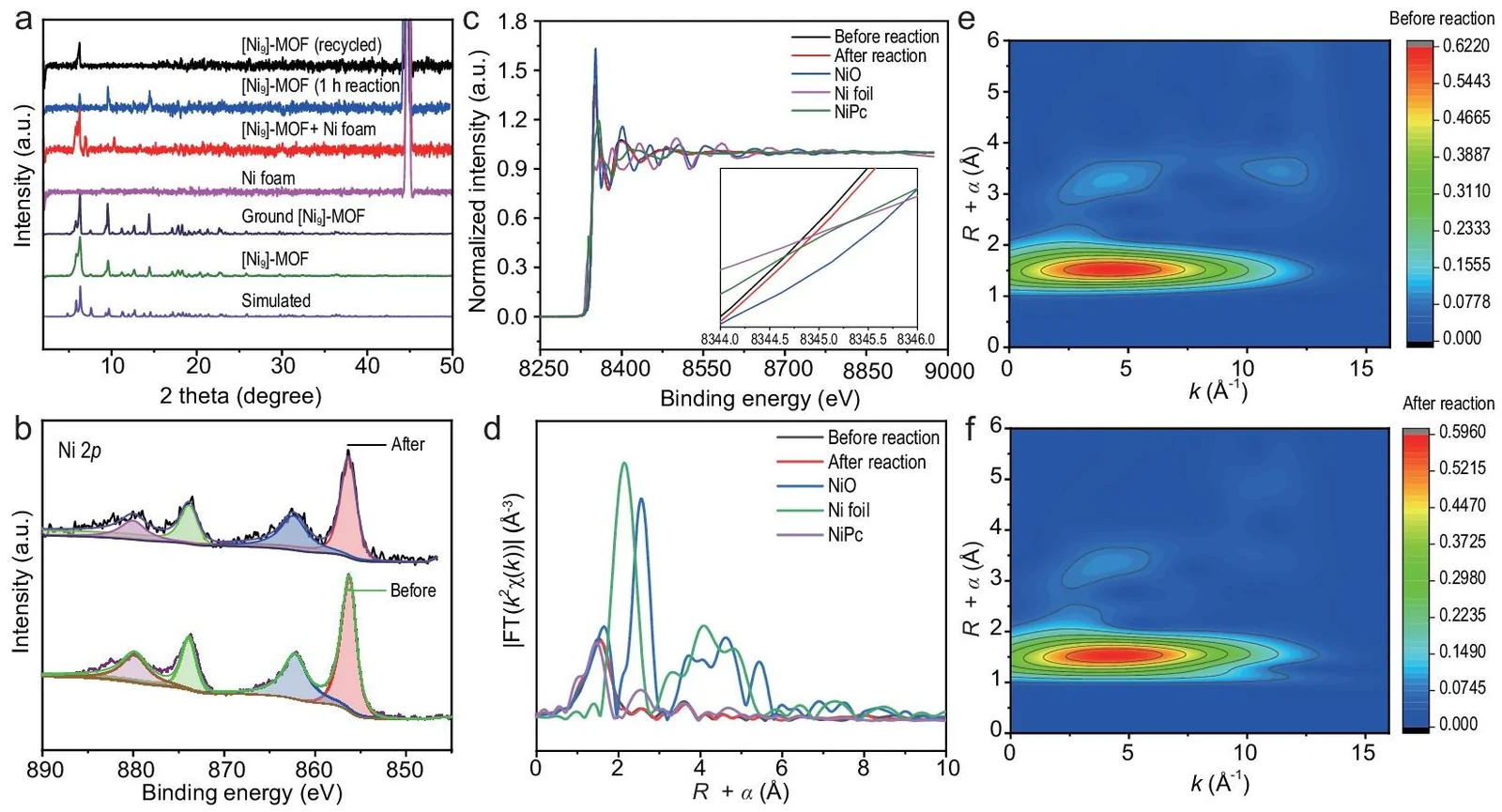
Stability characterization of **[Ni_9_]-MOF**. (a) PXRD of **[Ni_9_]-MOF** before and after the reaction. (b) XPS spectra of Ni 2*p* in **[Ni_9_]-MOF** before and after the reaction. Ni K-edge XANES (c) and FT-EXAFS (d) spectra of **[Ni_9_]-MOF** powder before and after the reaction. WT-EXAFS plots of **[Ni_9_]-MOF** powder before (e) and after (f) the reaction.

**Table 1.**
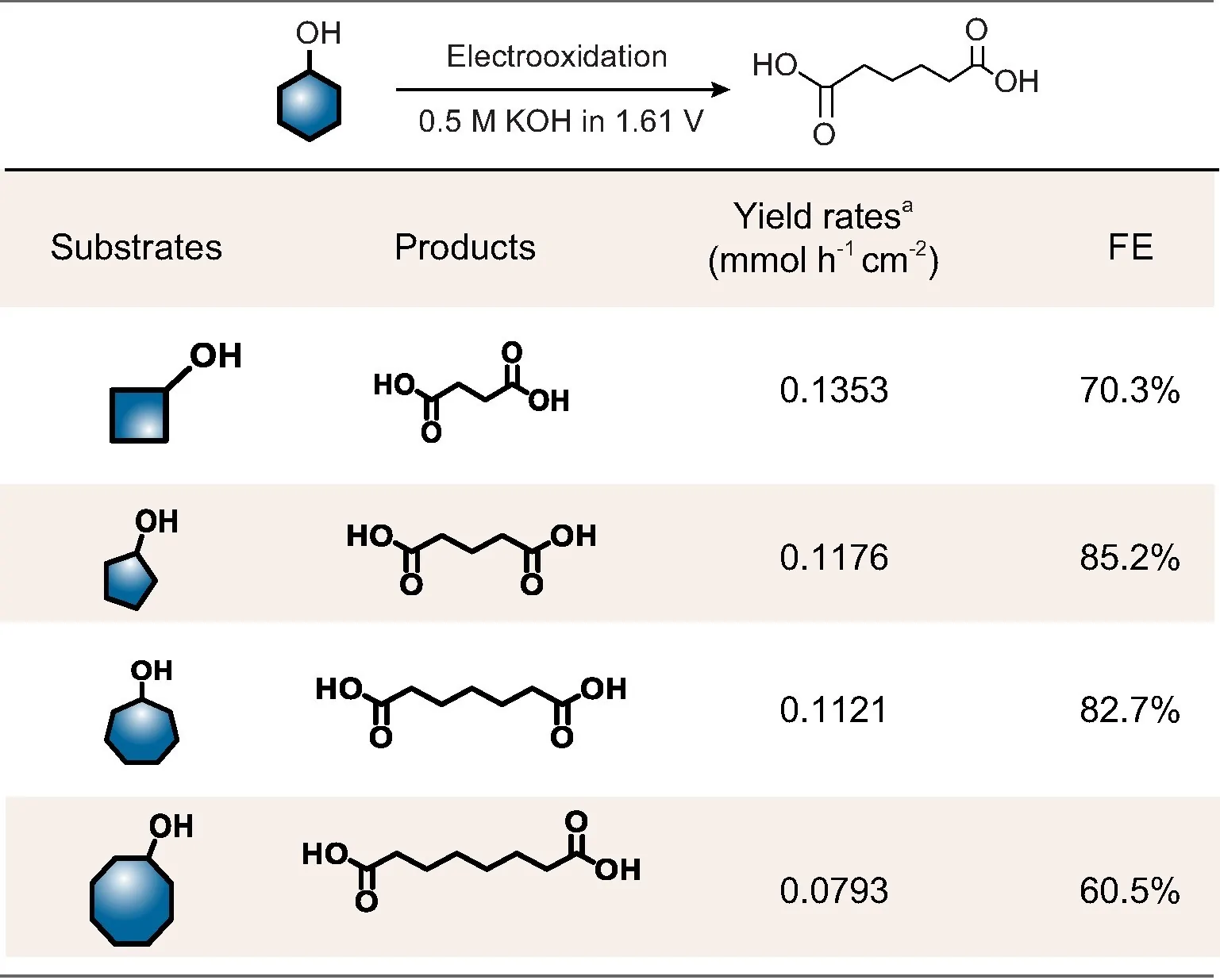
Electrolytic products and corresponding yield rates of various substrates.^a^

^a^[Ni_9_]-MOF was employed as an electrocatalyst for the oxidation of cyclobutanol, cyclopentanol, cycloheptanol, and cyclooctanol, with corresponding product yield rates and FE systematically quantified using ^1^H NMR.

### Reaction mechanism

Furthermore, the corresponding electrooxidation mechanism was explored through controlled experiments and characterization. Electron paramagnetic resonance (EPR) was initially performed to identify the possible intermediates using 5,5-dimethyl-1-pyrroline *N*-oxide (DMPO) as a spin-trapping agent. The signal of DMPO-C (marked by *) was clearly observed in the presence of cyclohexanol (Fig. 5a), but no obvious signal was observed without electricity. Through EPR signal simulation, the spectral features were successfully deconvoluted into contributions from both the DMPO-C adduct (A_N_ = 16.3 G, A_H_ = 23.7 G) and the oxidized DMPO species (DMPO-O) (A_N_ = 16.6 G) [51]. The above results revealed the formation and participation of carbon-centered radicals (*C) during

cyclohexanol oxidation process. To further verify the critical role of *C in the cyclohexanol oxidation process, we introduced the radical scavenger 2,2,6,6-tetramethylpiperidinyloxy (TEMPO), which could effectively quench *C. Upon TEMPO addition, the corresponding FE dropped from 87.6% to 46.2% (Fig. 5b), strongly supporting the *C-mediated reaction pathway. Furthermore, high-resolution mass spectrometry (HR-MS) was employed to identify potential intermediates formed during the oxidation process. As shown in Fig. 5c, the observed peak at 212.1565 corresponded to the composite of DMPO-cyclohexanone, consistent with the trapping of carbon-centered radicals. To capture transient reaction intermediates, hydroxylamine was employed as a chemical trapping agent. HR-MS analysis revealed a characteristic peak at *m/z* = 130.0835, being assigned to the condensation product formed between 2-hydroxycyclohexanone and hydroxylamine (Fig. 5d). This observation provided direct evidence for the formation of 2-hydroxycyclohexanone as a reactive intermediate during the electrocatalysis reaction. Furthermore, cyclohexanone was employed as the substrate to confirm these reaction intermediates using **[Ni_9_]-MOF**. CV and ^1^H NMR monitoring demonstrated the high electrocatalytic performance of **[Ni_9_]-MOF** in the electrochemical oxidation of cyclohexanone to AA, achieving an impressive FE of 88.4% with a production rate of 0.0685 mmol h^−1^ cm^−2^ at 1.51 V (Figs S44–S46). Owing to the inherently low aqueous solubility of cyclohexanone, utilizing cyclohexanone as the feedstock resulted in no observable yield enhancement; conversely, the overall product yield rate decreased. In addition, time-dependent ^1^H NMR analysis during electrolysis detected only the characteristic signals of cyclohexanol (*δ* = 3.66, 1.86, 1.71, 1.24 ppm) and AA (*δ* = 2.38, 2.23, 1.55 ppm), with no observable intermediates. This observation suggested the rapid conversion of reactive intermediates to the final product under the alkaline reaction conditions, thereby confirming their transient nature in the electrocatalytic synthesis.

**Figure 5.**
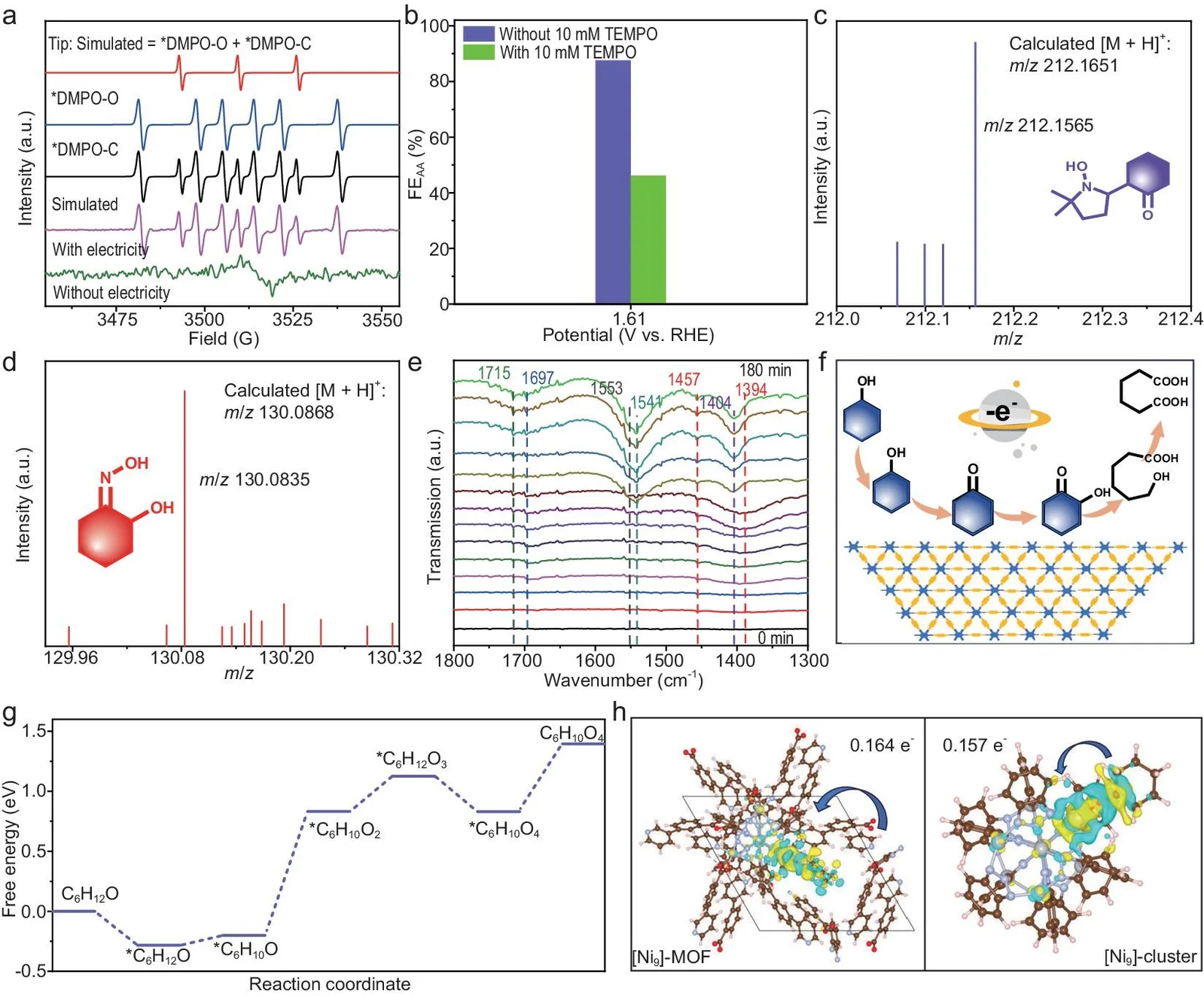
Reaction mechanism studies. (a) EPR signals with DMPO for cyclohexanol electrooxidation over **[Ni_9_]-MOF**. (b) Comparison of the yields of AA catalyzed by **[Ni_9_]-MOF** with and without 10 mM Tempo. (c) HR-MS of intermediates in the electrooxidation process of cyclohexanol captured by DMPO. (d) HR-MS of intermediates in the electrooxidation process of cyclohexanol captured by hydroxylamine. (e) *In situ* ATR-FTIR spectra of cyclohexanol electrooxidation over **[Ni_9_]-MOF**. (f) Proposed reaction mechanism. (g) Free energy diagram of **[Ni_9_]-MOF** catalyzed electrooxidation of cyclohexanol to AA. (h) The differential charge density at the interface between **[Ni_9_]-MOF** and **[Ni_9_]-cluster** in the presence of cyclohexanone was analyzed.

Subsequently, real-time monitoring of reaction intermediates was performed through *in situ* attenuated total reflection Fourier transform infrared spectroscopy (ATR-FTIR) during electrocatalysis, enabling precise tracking of transient species (Fig. 5e). Infrared spectra were collected over 180 min at 1.61 V in 0.5 M KOH and 0.1 M cyclohexanol. The cyclohexanol absorption band at 1457 and 1394 cm^−1^ exhibited progressive intensity decrease over the 180-min observation period, correlating with substrate conversion (Fig. S47). The gradual enhancement of the characteristic *ν*(C=O) stretching vibration at 1697 cm^−1^ was simultaneously observed, corresponding to cyclohexanone formation (Fig. S48). This spectral evolution aligned with above ^1^H NMR results, which confirmed the gradual accumulation of cyclohexanone as a key intermediate throughout the reaction timeline. The characteristic peaks of AA at 1553 and 1404 cm^−1^ enhanced with the reaction (Fig. S49). A transient peak at 1715 cm^−1^, assigned to 2-hydroxycyclohexanone, initially increased before declining, indicating rapid intermediate conversion (Fig. S50). Similarly, the characteristic peak of 6-hydroxyhexanoic acid at 1541 cm^−1^ showed comparable behavior (Fig. S51). The ATR-FTIR results demonstrated that **[Ni_9_]-MOF** catalyzed the electrooxidation of cyclohexanol through a sequential pathway involving cyclohexanone, 2-hydroxycyclohexanone and 6-hydroxyhexanoic acid as key intermediates.

Then, to validate the proposed reaction pathway, the electrochemical oxidation activity of strategically selected intermediates was systematically evaluated. When employing 2-hydroxycyclohexanone and 6-hydroxyhexanoic acid as substrates, AA formation rates reached 0.2015 and 0.2078 mmol h^−1^ cm^−2^, respectively (Figs S54 and S55). Kinetic analysis revealed a distinct substrate-dependent progression: AA production rates increased sequentially across the reactant series, cyclohexanol (0.1239 mmol h^−1^ cm^−2^), 2-hydroxycyclohexanone (0.2015 mmol h^−1^ cm^−2^) and 6-hydroxyhexanoic acid (0.2078 mmol h^−1^ cm^−2^), demonstrating accelerated reaction kinetics with intermediates structurally closer to the final product. Notably, cyclohexanone oxidation yielded an intermediate rate of 0.0814 mmol h^−1^ cm^−2^ (1.61 V), deviating from this progression due to solubility limitations. This kinetic hierarchy provided compelling evidence supporting the sequential formation of these intermediates in the proposed mechanism. Notably, a postulated intermediate of 1,2-cyclohexanedione generated glutaric acid (C_5_ byproduct) under alkaline conditions via radical-mediated pathways (Fig. S56). These results confirmed the involvement of cyclohexanone, 2-hydroxycyclohexanone and 6-hydroxyhexanoic acid as critical intermediates in the electrooxidation of cyclohexanol to AA.

Integrated experimental analyses confirmed an electrocatalytically mediated nucleophilic oxidation pathway for cyclohexanol-to-AA conversion over **[Ni_9_]-MOF** (Fig. 5f). The reaction initiated with potential-controlled adsorption of cyclohexanol onto the catalyst surface. This primary alcohol underwent sequential oxidation: first to cyclohexanone, then to 2-hydroxycyclohexanone via *α*-C-H activation facilitated by **[Ni_9_]-MOF**. Subsequent oxidative cleavage of the C(1)–C(2) bond generated 6-hydroxyhexanoic acid as a key intermediate. The catalytic cycle culminated in the selective oxidation of this species to AA with >85% FE, demonstrating the catalyst’s exceptional catalytic activity under electrochemical conditions.

DFT calculations were performed to further explore the interactions between **[Ni_9_]-MOF** and reactive intermediates during the reaction process. The geometries of **[Ni_9_]-MOF** and **[Ni_9_]-cluster** were optimized using the Vienna Ab initio Simulation Package with the Projector Augmented Wave method, the Perdew-Burke-Ernzerhof functional within the generalized gradient approximation and the DFT-D3 dispersion correction. The optimized structures are shown in Figs S59 and S60. Gibbs free energy profiles for the electrooxidation of cyclohexanol to AA on **[Ni_9_]-MOF** were computed based on optimized adsorption configurations, proposing a plausible oxidation pathway (Fig. 5g). **[Ni_9_]-MOF** facilitated dynamic coordination bonding, where cyclohexanol initially adsorbed onto an unsaturated Ni site to form the activated intermediate *C_6_H_12_O (Fig. S61). Subsequent dehydrogenation generated *C_6_H_10_O (cyclohexanone) (Fig. S62), which was further oxidized to *C_6_H_10_O_2_ (2-hydroxycyclohexanone) (Fig. S63), as verified by HR-MS and FTIR spectroscopy. The transformation of *C_6_H_10_O to *C_6_H_10_O_2_ was identified as the potential-determining step with an energy barrier of 1.03 eV for **[Ni_9_]-MOF** (Fig. 5g). Then, *C_6_H_10_O_2_ was rapidly converted to *C_6_H_12_O_3_ (6-hydroxyhexanoic acid) (Fig. S64), which was deeply oxidized to *C_6_H_10_O_4_ (AA) under alkaline conditions (Fig. S65). The differential charge density revealed that cyclohexanone adsorption transferred 0.164 electrons to **[Ni_9_]-MOF** framework, compared to 0.157 electrons for **[Ni_9_]-cluster** (Fig. 5h). Differential charge density maps confirmed enhanced electron redistribution at the cyclohexanone–**[Ni_9_]–MOF** interface, promoting efficient charge transfer. These DFT results further elucidated that the porous architecture of **[Ni_9_]-MOF** accelerated the conversion of cyclohexanone to 2-hydroxycyclohexanone.

Based on the reaction mechanism, theoretical calculations and experimental results proposed in previous literature, a nucleophilic oxidation reaction pathway for the electrosynthesis of AA from cyclohexanol was proposed (Fig. S66): **Path 1** is the operative nucleophilic oxidation route, in which initial substrate adsorption is followed by two-electron dehydrogenation to cyclohexanone, and then by electrolyte-driven hydroxylation to 2-hydroxycyclohexanone. At this critical branching point, **Path 2** (cyclohexanedione to glutaric acid) was excluded due to product incongruence with observed AA formation. Similarly, **Path 3** (adipic anhydride intermediate) was thermodynamically precluded by its prohibitive activation energy barrier. Consequently, the activated carbonyl intermediate underwent hydroxide-triggered *β*-carbon C–C bond cleavage, producing 6-hydroxyhexanoic acid that is then selectively oxidized to AA with over 85% FE. This work definitively established **Path 1** while providing unprecedented mechanistic insight into electrocatalytic intermediate evolution.

Electrochemical impedance spectroscopy (EIS) and kinetic analyses were conducted to explore the enhanced electrooxidation performance of **[Ni_9_]-MOF**. Nyquist plots acquired at different potentials revealed interfacial characteristics between the electrode and electrolyte. For the OER, the Bode phase angle plot of **[Ni_9_]-MOF** exhibited a characteristic low-frequency inflection point at 1.46 V, indicating OER onset (Fig. S67a). Upon introducing cyclohexanol, this inflection shifted to 1.41 V, demonstrating that cyclohexanol oxidation occurred at a lower potential than OER (Fig. S68b), thereby suppressing competitive oxygen evolution. The phase angle decreased progressively with increasing applied potential, suggesting accelerated interfacial charge transfer during cyclohexanol oxidation. Corresponding EIS spectra (Fig. S68c and d) aligned with Bode plot trends. Notably, while **[Ni_9_]-cluster** analogue followed similar electrochemical behavior (Fig. S68), its larger phase angle magnitude compared to **[Ni_9_]-MOF** underscored the superior charge transfer kinetics of the 2D MOF architecture over discrete cluster-based systems.

A custom dual-electrode flow reactor was engineered to assess the industrial viability of high-current-density electrochemical systems. The electrochemical cell comprised a **[Ni_9_]-MOF** working electrode and nickel foam counter electrode, separated by a low contact-resistance FAA-3-50 anion-exchange membrane (AEM) (Fig. 6a). Electrolytes were continuously circulated (20 mL min^−1^) via peristaltic pumps through isolated loops containing: (i) 0.5 M KOH catholyte and (ii) 0.5 M KOH with 0.1 M cyclohexanol anolyte. LSV revealed a substantial voltage reduction from 2.38 to 2.00 V at 0.75 A upon cyclohexanol introduction compared to KOH electrolyte (Fig. 6b), indicating significantly decreased onset potentials at equivalent current densities due to electrocatalytic activation. The result demonstrated the catalyst’s retained efficacy for cyclohexanol oxidation in MEA configurations. To evaluate the performance of **[Ni_9_]-MOF** under different applied currents, subsequent constant-current electrolysis experiments were performed between 0.25 A to 2.0 A. These experiments employed an electrolyte solution of 0.5 M KOH and 0.1 M cyclohexanol, enabling the determination of the FE and AA yield for each condition. At a current of 0.5 A (0.125 A cm^−2^), the system reached a peak production rate of 1.94 mmol h^−1^ with an FE of 83% over a 2-h electrolysis period (Fig. 6c), accompanied by hydrogen evolution at a rate of 8.39 mmol h^−1^. Extended stability testing under MEA configuration (Fig. 6d) exhibited low voltage decay over 32 hours (from 2.76 to 2.36 V), confirming exceptional operational durability. The reactor successfully converted cyclohexanol to AA within 32 h (89.3% conversion rate). In addition, this system can also convert KA oil (with a molar ratio of cyclohexanol to cyclohexanone of 3) into AA coupled with HER. At a current of 0.5 A, both AA (15.6 mmol) and H_2_ (67.4 mmol) were simultaneously obtained in 10 h. This proof-of-concept demonstrated gram-scale AA and H_2_ production through MEA electrolysis, establishing foundational parameters for industrial implementation while highlighting the excellent performance of **[Ni_9_]-MOF** in electrocatalytic AA synthesis.

**Figure 6.**
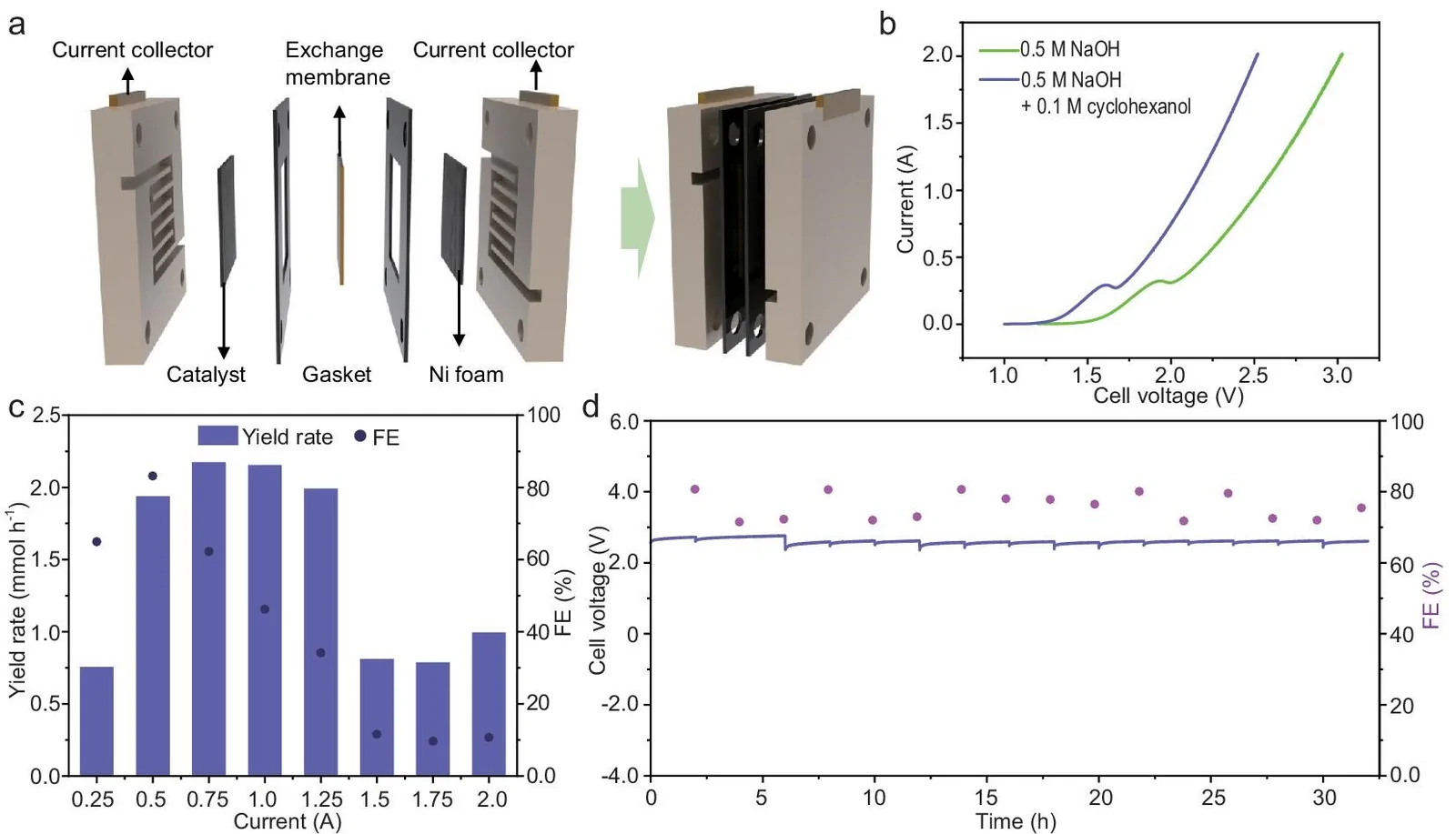
A two-electrode flow reactor for AA production. (a) Schematic illustration of a two-electrode flow reactor (4 cm^2^). (b) LSV curves of the MOF-based membrane electrode were systematically compared in 0.5 M KOH electrolyte with and without 0.1 M cyclohexanol. (c) Current density-dependent FEs and AA yield rates were systematically studied using **[Ni_9_]-MOF**. (d) Durability test in a two-electrode flow electrolyzer at 0.5 A.

## CONCLUSION

In summary, this work successfully synthesized a novel 2D framework **[Ni_9_]-MOF** through a bottom-up assembly strategy from a discrete **[Ni_9_]-cluster**, forming [Ni_36_] cages with dimensions of 14.4 × 14.4 × 10.0 Å^3^. **[Ni_9_]-MOF** exhibited excellent acid–base stability in both 8 M NaOH and 1 M HCl, which was attributed to its pronounced hydrophobicity in alkaline environments, as confirmed by contact angle measurements. Electrocatalysis results revealed that **[Ni_9_]-MOF** achieved a remarkable AA production rate of 0.1239 mmol h^−1^ cm^−2^ at 1.61 V, significantly surpassing the discrete **[Ni_9_]-cluster** (0.0749 mmol h^−1^ cm^−2^) and ranking among the highest values reported to date. The catalyst maintained high activity over 25 cycles without notable degradation. Substrate scope expansion revealed its versatility in synthesizing C_4_-C_8_ dicarboxylic acids (succinic, glutaric, adipic, heptanedioic and suberic acids) with FEs exceeding 60%. When implemented in an MEA, the production rate further increased to 1.94 mmol h^−1^, accompanied by H_2_ generation at 8.39 mmol h^−1^, highlighting its practical potential. Mechanistic studies identified a three-step electrooxidation pathway from cyclohexanol to AA: (i) cyclohexanol oxidation to cyclohexanone, (ii) rate-determining hydroxylation to 2-hydroxycyclohexanone (energy barrier: 1.03 eV) and (iii) oxidative cleavage to 6-hydroxyhexanoic acid followed by final conversion to AA. DFT calculations revealed an increased electron density at the [Ni_9_] active sites, from 0.157 e^−^ per site in the isolated **[Ni_9_]-cluster** to 0.164 e^−^ in **[Ni_9_]-MOF**. This electronic enhancement rationalizes the accelerated oxidation kinetics and superior catalytic performance. This study not only introduces a robust MOF design strategy based on polynuclear clusters but also establishes the first MOF electrocatalyst for AA synthesis, opening avenues for sustainable electrochemical conversions.

## MATERIALS AND METHODS

Preparation of {[Ni_9_(btz)_12_(NO_3_)_6_(DMA)_6_]·3DMA}*_n_*  **([Ni_9_]-cluster)**: A mixture of Ni(NO_3_)_2_·6H_2_O (0.3 mmol), Hbtz (0.4 mmol) in DMA and CH_3_OH (1:1, 5 mL) were sealed in a 25-mL Teflon-lined stainless autoclave, and then heated at 100°C for 24 h. After cooling down to room temperature, blue cubic crystals were obtained and washed with DMA for several times. CCDC number: 1569801.

Preparation of {[Ni_9_(btz)_12_(3-pba)_6_]·16H_2_O·8DMF·4CH_3_OH}*_n_* (**[Ni_9_]-MOF**): A mixture of **[Ni_9_]-cluster** (15 mg), 3-Hpba (0.1 mmol), 50 μL HNO_3_ in DMF and CH_3_OH (1:1, 3 mL) were sealed in a 25-mL Teflon-lined stainless autoclave, and then heated at 150°C for 72 h. After cooling down to room temperature, blue cubic crystals were obtained and washed with DMF for several times. CCDC number: 2492643.

## Supplementary Material

nwag427_Supplemental_Files
