# Supplementary material for: Highly recyclable and efficient electrocatalytic synthesis of adipic acid catalyzed by ultra-alkali-resistant [Ni36] nanocages in MOF
Source: Natl Sci Rev. 2026 Jul 11;13(19):nwag427. doi: 10.1093/nsr/nwag427 (PMC13678815; doi:10.1093/nsr/nwag427)

checkCIF/PLATON report

You have not supplied any structure factors. As a result the full set of tests cannot be run.

THIS REPORT IS FOR GUIDANCE ONLY. IF USED AS PART OF A REVIEW PROCEDURE FOR PUBLICATION, IT SHOULD NOT REPLACE THE EXPERTISE OF AN EXPERIENCED CRYSTALLOGRAPHIC REFEREE.

No syntax errors found. CIF dictionary Interpreting this report

Datablock: Ni9-MOF

```
Bond precision:   C-C = 0.0229 A          Wavelength=0.71073

Cell:              a=17.3771(5)      b=17.3771(5)      c=36.4536(18)
                   alpha=90          beta=90           gamma=120
Temperature:       193 K
```

	Calculated	Reported
Volume	9532.9(7)	9532.9(7)
Space group	P 63	P 63
Hall group	P 6c	P 6c
Moiety formula	C48 H32 N14 Ni3 O4 [+ solvent]	C48 H32 N14 Ni3 O4
Sum formula	C48 H32 N14 Ni3 O4 [+ solvent]	C48 H32 N14 Ni3 O4
Mr	1044.95	1045.00
Dx,g cm-3	1.092	1.092
Z	6	6
Mu (mm-1)	0.924	0.924
F000	3204.0	3204.0
F000'	3210.96	
h,k,lmax	20,20,43	20,20,43
Nref	11728[5963]	11162
Tmin,Tmax	0.912,0.912	0.584,0.751
Tmin'	0.912	

```
Correction method= # Reported T Limits: Tmin=0.584 Tmax=0.751
AbsCorr = MULTI-SCAN

Data completeness= 1.87/0.95       Theta(max)= 25.397

R(reflections)= 0.1092( 9145)                     wR2(reflections)=
                                                  0.2830( 11162)
S = 1.038                 Npar= 305
```

The following ALERTS were generated. Each ALERT has the format
test-name_ALERT_alert-type_alert-level.
Click on the hyperlinks for more details of the test.

Alert level B

```
PLAT341_ALERT_3_B Low Bond Precision on  C-C Bonds ..............     0.0229 Ang.
```

Alert level C

```
CRYSC01_ALERT_1_C No recognised colour has been given for crystal colour.
STRVA01_ALERT_4_C           Flack test results are ambiguous.
           From the CIF: _refine_ls_abs_structure_Flack    0.320
           From the CIF: _refine_ls_abs_structure_Flack_su    0.060
PLAT082_ALERT_2_C High R1 Value ................................       0.11 Report
PLAT084_ALERT_3_C High wR2 Value (i.e. > 0.25) ..................       0.28 Report
PLAT220_ALERT_2_C NonSolvent   Resd 1  C   Ueq(max)/Ueq(min) Range       3.2 Ratio
PLAT220_ALERT_2_C NonSolvent   Resd 1  N   Ueq(max)/Ueq(min) Range       3.9 Ratio
PLAT241_ALERT_2_C High   'MainMol' Ueq as Compared to Neighbors of        O1 Check
PLAT241_ALERT_2_C High   'MainMol' Ueq as Compared to Neighbors of        O2 Check
PLAT241_ALERT_2_C High   'MainMol' Ueq as Compared to Neighbors of      C027 Check
PLAT242_ALERT_2_C Low    'MainMol' Ueq as Compared to Neighbors of      O00J Check
PLAT242_ALERT_2_C Low    'MainMol' Ueq as Compared to Neighbors of      N009 Check
PLAT242_ALERT_2_C Low    'MainMol' Ueq as Compared to Neighbors of        C1 Check
```

Alert level G

```
PLAT002_ALERT_2_G Number of Distance or Angle Restraints on AtSite         47 Note
PLAT003_ALERT_2_G Number of Uiso or U(i,j)  Restrained non-H-Atoms         66 Report
PLAT004_ALERT_5_G Polymeric Structure Found with Maximum Dimension          2 Info
PLAT012_ALERT_1_G N.O.K.   _shelx_res_checksum Found in CIF ......    Please Check
PLAT033_ALERT_4_G Flack x Value Deviates > 3.0 * Sigma from Zero .      0.320 Note
PLAT072_ALERT_2_G SHELXL First  Parameter in WGHT  Unusually Large       0.13 Report
PLAT083_ALERT_2_G SHELXL Second Parameter in WGHT  Unusually Large     103.49 Why ?
PLAT171_ALERT_4_G The CIF-Embedded .res File Contains EADP Records          7 Report
PLAT172_ALERT_4_G The CIF-Embedded .res File Contains DFIX Records         43 Report
PLAT186_ALERT_4_G The CIF-Embedded .res File Contains ISOR Records         21 Report
PLAT187_ALERT_4_G The CIF-Embedded .res File Contains RIGU Records          1 Report
PLAT606_ALERT_4_G Solvent Accessible VOID(S) in  Crystal Structure          ! Info
PLAT720_ALERT_4_G Number of Unusual/Non-Standard Labels ..........         79 Note
              Ni01    Ni02    Ni03    Ni04    Ni05    N006    N007    C1AA
              H1AA    N008    N009    C00A    N00B    N00C    N00E    N00F
              N00G    C00H    N00I    O00J    C00K    H00K    N00L    C00N
              N00P    N00Q    C00R    C00W    H00W    C00Y    H00Y    C00Z
```

```
              H00Z    C013    H013    C014    H014    C01D    H01D    C01E
              C016    H016    C01U    C01T    H01T    C024    H024    C01Y
              H01Y    C01G    C010    C01L    H01L    C01O    H01O    C01H
              H01H    C5AA    N00V    C02O    H02O    C01S    H01S    C027
              C02E    C01F    H01F    C015    H015    C012    H012    C00O
              H00O    C00U    C4AA    C3AA    H3AA    C0AA    H0AA
PLAT794_ALERT_5_G Tentative Bond Valency for Ni01      (II)       .       1.98 Info
PLAT794_ALERT_5_G Tentative Bond Valency for Ni02      (II)       .       2.06 Info
PLAT794_ALERT_5_G Tentative Bond Valency for Ni03      (II)       .       1.99 Info
PLAT794_ALERT_5_G Tentative Bond Valency for Ni04      (II)       .       2.14 Info
PLAT794_ALERT_5_G Tentative Bond Valency for Ni05      (II)       .       1.96 Info
PLAT860_ALERT_3_G Number of Least-Squares Restraints .............         440 Note
PLAT869_ALERT_4_G ALERTS Related to the Use of SQUEEZE  Suppressed           ! Info
PLAT933_ALERT_2_G Number of HKL-OMIT Records in Embedded .res File         136 Note
               -3  5  2,  -1  4  0,  -3  4  6,  -3  5 -2,  -3  4  5,  -3  4 -5,
               -1  2  0,  -3  4 -6,  -1  5 -4,  -1  5  4,  -1  4  1,  -1  4  2,
               -2  3 -6,  -2  3  6,  -1  4 -1,  -5  6  1,   0  1  2,   0  1 -2,
               -1  4 -2,  -1  2 -3,  -1  2  3,  -1  9 -2,  -1 10  2,  -1  9  2,
               -5  6 -1,  -2  4 -3,  -2  4  3,  -1 10 -2,   0  2  4,  -2  3  5,
                0  2 -4,  -4  7 14,  -2  9 -3,  -2  9  3,  -1  5-13,  -4  7-14,
                0  1  0,   0  1  1,  -1  9  6,  -6  8  3,  -1  9 10,   0  1 -1,
               -1  9 -6,  -1  5 13,  -4  9  0,  -2  3 -5,  -4 17 -3,  -4 17  3,
               -3  4 -7,  -2  3  3,
```

```
  0 ALERT level A = Most likely a serious problem - resolve or explain
  1 ALERT level B = A potentially serious problem, consider carefully
 12 ALERT level C = Check. Ensure it is not caused by an omission or oversight
 21 ALERT level G = General information/check it is not something unexpected

  2 ALERT type 1 CIF construction/syntax error, inconsistent or missing data
 14 ALERT type 2 Indicator that the structure model may be wrong or deficient
  3 ALERT type 3 Indicator that the structure quality may be low
  9 ALERT type 4 Improvement, methodology, query or suggestion
  6 ALERT type 5 Informative message, check
```

It is advisable to attempt to resolve as many as possible of the alerts in all categories. Often the minor alerts point to easily fixed oversights, errors and omissions in your CIF or refinement strategy, so attention to these fine details can be worthwhile. It is up to the individual to critically assess their own results and, if necessary, seek expert advice.

PLATON version of 26/09/2025; check.def file version of 20/09/2025

---

duplicate check

No duplication found

Datablock Ni9-MOF - ellipsoid plot

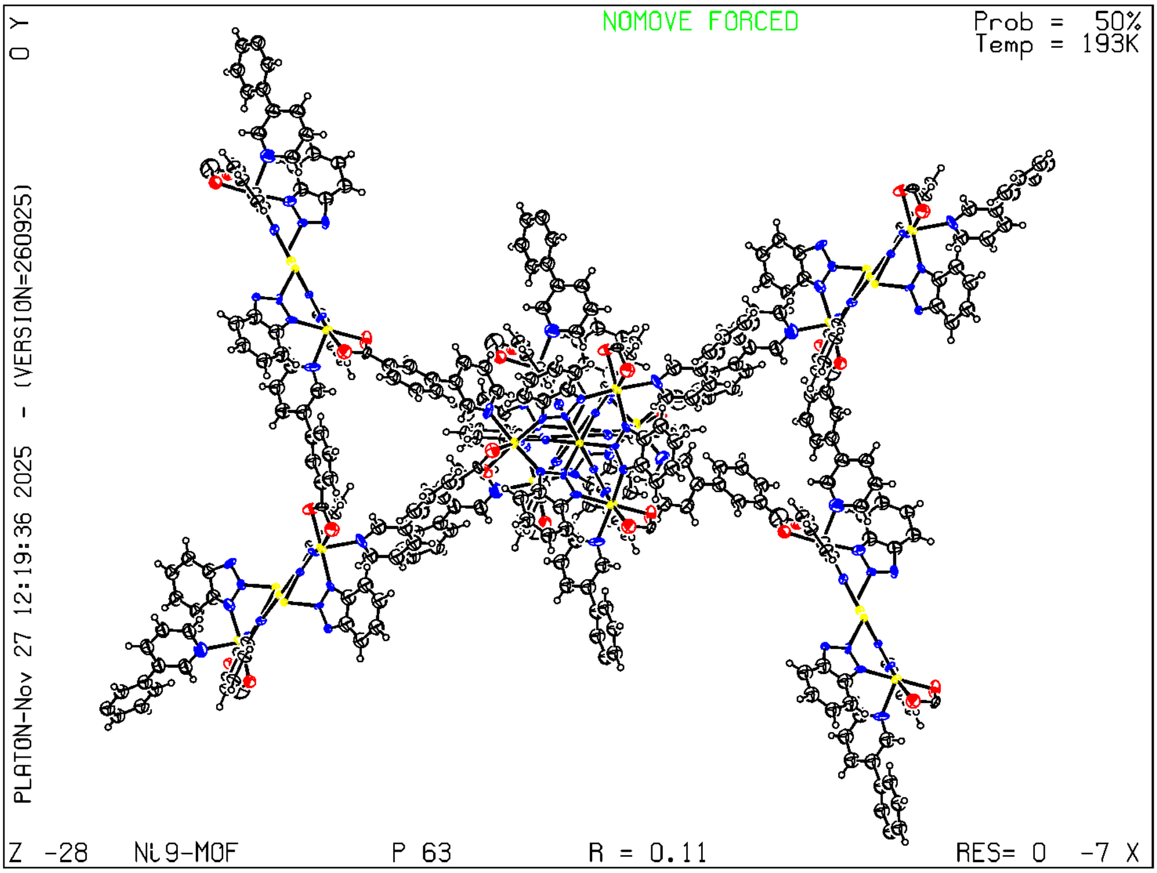

Supplement: Supplementary file 1 [file nwag427_supplemental_files.zip › CIFs-2026-660.R2/checkcif.pdf]
