# Supplementary material for: Highly recyclable and efficient electrocatalytic synthesis of adipic acid catalyzed by ultra-alkali-resistant [Ni36] nanocages in MOF
Source: Natl Sci Rev. 2026 Jul 11;13(19):nwag427. doi: 10.1093/nsr/nwag427 (PMC13678815; doi:10.1093/nsr/nwag427)
Supplement: Supplementary file 1 [file nwag427_supplemental_files.zip › Supporting_Information-NSR_MS-2026-660.R2.pdf]

Supporting Information for

Highly recyclable and efficient electrocatalytic synthesis of adipic acid catalyzed by ultra-alkali-resistant [Ni_{36}] nanocages in MOF

Ze-Long Liang[1], Rui Li[3], Zhen-Ting Wang[1], Jia-Can Lv[1], Xin-Min Ai[1], Qi Li[1], Ying-Ying Yu[1], Yang-Xi Wang[1], Fang-Ting Zhu[1], Qi-Fan Wang[1], Hang Xu[1,*], and Bin Zhao[1,2,*]

[1]Frontiers Science Center for New Organic Matter, Key Laboratory of Advanced Energy Materials Chemistry (MOE), College of Chemistry, Nankai University, Tianjin 300071, China;

[2]Ganjiang Innovation Academy, Jiangxi Institute of Rare Earths, Chinese Academy of Sciences, Ganzhou 341000, China and [3]School of Medicine, Nankai University, Tianjin 300071, China

***Corresponding authors.** E-mails: zhaobin@nankai.edu.cn; xuhang@nankai.edu.cn

Supporting Information Text

Section S1. Materials

Nickel nitrate ($Ni(NO_3)_2 \cdot 6H_2O$), benzotriazole (Hbtz) were purchased from Beijing Innochem Science & Technology Co., Ltd. Ethanol, methanol, dichloromethane were from Alfa Aesar China Co., Ltd. Organic ligand 3-(3-Pyridinyl)Benzoic Acid (3-Hpba) was purchased commercially from Chemextenesion. N, N-Dimethylacetamide (DMA) and N, N-Dimethylformamide (DMF), isopropanol, dimethyl sulfoxide (DMSO) and Nitric acid were purchased from Hengshan chemical corporation. D_2O and Potassium hydroxide (KOH) were purchased from Adamas. 5 wt% Nafion solution was bought from Alfa Aesar. Cyclohexanol, cyclobutanol, cyclopentanol, cycloheptanol, cyclooctanol, 1,2-cyclohexanedione, 5,5-dimethyl-1-pyrroline N-oxide (DMPO), and 2,2,6,6-tetramethylpiperidinyl-1-oxide (TEMPO) were obtained from Bide Pharmatech Co., Ltd. 6-Hydroxyhexanoic acid and hydroxylamine were procured from Energy Chemical. 2-Hydroxycyclohexan-1-one was acquired from Leyan Reagent Company. FAA-3-PK-130 and FAA-3-50 were supplied by Sineros Technology Co., Ltd. Ni foam (NF, thickness: 1.6 mm; porosity: ~ 98%) was purchased from Guangshengjia New Material Co., Ltd. The rest of the reagents were purchased from commercial channels as same. All reagents were used directly without purification.

Section S2. Materials characterization

Single-crystal X-ray diffraction analysis was conducted on an XtaLAB Synergy-R diffractometer equipped with a PhotonJet-R microfocus rotating anode Cu Kα source (λ = 1.54 Å), a high-speed κ-goniometer, and a HyPix HPC detector. The crystal structure was determined via direct methods using the OLEX 2 software and refined to convergence through full-matrix least-squares minimization on F^2. Powder X-ray diffraction (PXRD) data were collected at ambient temperature on a Rigaku Ultima IV diffractometer with Cu Kα radiation (40 kV, 40 mA). Solution-state ^1H and ^{13}C NMR spectra were acquired on a Bruker 400 MHz spectrometer using D_2O as the solvent. Thermogravimetric analysis (TGA) was performed on a NETZSCH TG209 instrument under a nitrogen atmosphere at a heating rate of 5 °C min^{-1}. Scanning electron microscopy (SEM) and energy-dispersive X-ray spectroscopy (EDS) were carried out using a Hitachi SU3500 microscope coupled to a Bruker Quantax EDS system. Nitrogen adsorption-desorption isotherms were measured at 77 K on a V-Sorb 2800P analyzer, with surface area and porosity derived via Brunauer-Emmett-Teller (BET). Prior to analysis, samples were degassed at 120 °C for 10 h. Fourier-transform infrared (FT-IR) spectra were recorded on a ThermoFisher Nicolet iS50 spectrometer. Elemental composition was analyzed by inductively coupled plasma mass spectrometry (ICP-MS; SPECTRO-BLUE) and atomic emission spectroscopy (ICP-AES). Gas chromatography (GC; Au-light Technologies) quantified reaction conversion and selectivity, while high-resolution mass spectrometry (HR-MS) data were obtained using a Waters Synapt-G2-Si UPLC-TOFMS system. X-ray photoelectron spectroscopy (XPS) studies were performed on a Thermo Fisher ESCALAB Xi^+ instrument, with binding energies referenced to the C 1*s* peak at 284.8 eV. The X-ray absorption (XAS) spectra at the Ni K-edges were recorded in transmission mode on a Table XAFS-500-A speciation instrument. N_2 adsorption at 77 K were used to calculate pore volumes. All characterizations, including NMR, GC, and XPS, were conducted at room temperature unless noted. Product yields for adipic acid are reported as NMR values. A liquid chromatography–mass spectrometer (LC–MS) was used on an Agilent 6550 QTOF instrument. Accurate mass measurements of the products and intermediates were acquired via Waters ACQUITY UPLC I-Class/XEVO G2-XS QTOF high-resolution mass spectrometry (HR-MS, ESI). Radicals were explored via electron paramagnetic resonance (EPR) spectroscopy (E580-10/12, BRUKER, American).

Section S3. Experimental procedures

PXRD Testing. PXRD patterns of **[Ni_9]-cluster** and **[Ni_9]-MOF** after immersing in aqueous solvents with different pH for 24 h. Typically, 50 mg of **[Ni_9]-cluster** and **[Ni_9]-MOF** were immersed in 20 mL corresponding aqueous solvents respectively and change the solution every three hours. After 24 h, the processed samples were collected and washed by centrifugation. The samples were dried in vacuum oven at room temperature overnight and ground before PXRD test. The solution used in the experiment is adjusted to pH value by HCl and NaOH. In addition, PXRD patterns of **[Ni_9]-cluster** and **[Ni_9]-MOF** after immersing in common organic solvents for 24 h (H_2O, MeOH, DMF, CH_3CN, CH_2Cl_2, CH_3CH_2OH, DMA). Typically, 50 mg of **[Ni_9]-cluster** and **[Ni_9]-MOF** were immersed in 20 mL corresponding solvents respectively and sealed for 24 h. The processed samples were collected and washed by centrifugation. After drying in vacuum oven at 80 °C overnight, the samples were ground before PXRD test.

Section S4. Electrochemical measurements.

All electrochemical tests are conducted on CHI760e electrochemical workstation (Shanghai Chenhua Instrument Co., LTD.), using potassium hydroxide as electrolyte sealed flow cell. In the three-electrode

system, platinum electrode, Hg/HgO electrode and FAA-3-PK-130 are used as the counter electrode, the reference electrode and the anion exchange membrane, respectively. Each compartment contained 15 mL electrolyte. The reference electrode was Hg/HgO and this was placed in the anode compartment near the working electrode. Powder sample (16 mg), isopropyl alcohol (1.6 mL) and Nafion solution (80 μL) were used as catalyst, dispersant and binder, respectively, to form uniform ink after ultrasound. The electrolyte is made from Cyclohexanol into 0.5 M KOH dissolved in ultra-pure Milli-Q water for 30 min. The ultrasonic ink was dropped on Ni foam of 2 × 2 cm^2 to ensure that the Ni foam load was controlled at 1 mg cm^{-2}. After the Ni foam is dried, the flow pool is assembled as an electrolytic cell, and the electrolyte circulation pump are opened. The flow rate of the electrolyte stream was 5 mL min^{-1}, controlled by a peristaltic pump unless otherwise noted. The linear voltammetry curve scan rate is 10 $mV \cdot s^{-1}$. Linear voltammetry data are tested without IR compensation. Electrochemical impedance spectroscopy (EIS) was tested in the 5 mV range of 0.01-10^5 Hz. The resistance (R) was determined as the intersection of the curve with the real axis of the Nyquist plot. Current densities were calculated with respect to the catalyst-covered geometric area of the working electrode. For the cyclohexanol oxidation experiments, unless otherwise stated, the electrolyte was 0.5 M aqueous KOH. To evaluate the performance of electrocatalytic cyclohexanol oxidation, controlled potential electrolysis was performed for 60 min in a 15 mL electrolyte at different potentials. During the experiment, the flow rate of the electrolyte circulating in the peristaltic pump was also controlled at 5 mL min^{-1}. The formula for converting the potential to a reversible hydrogen electrode is as follows:

$$E_{(RHE)}=E_{(Hg/HgO)} + 0.098 + 0.0591 * pH$$

The liquid phase products were detected by nuclear magnetic resonance spectrometry. 100 μL electrolyte was added to 375 μL D_2O with DMSO (dimethyl sulfoxide) as the internal standard. According to the content of DMSO internal standard, the molar number of liquid products was determined by integral method. The Faraday efficiency (FE_{liquid}) of liquid products is calculated by:

$$FE_{liquid} = \frac{Z \times F \times V_{electrolyte} \times C_{DMSO} \times S_{liquid}}{Q \times S_{DMSO}} \times 100\%$$

F: Faraday constant is 96485 C mol^{-1}; Q: the total coulomb amount after electrolysis; Z: The number of transferred electrons required for the conversion from cyclohexanol to adipic acid; C_{DMSO}: Molar concentration of DMSO internal standard, mol L^{-1}; $V_{electrolyte}$: electrolyte total volume, mL; S_{liquid}: integral area of the liquid product; S_{DMSO}: Integral area of DMSO. The integral area is normalized by the number of hydrogen atoms.

The reaction rate was calculated according to the following equation:

$$Reaction\ rate\ (mmol\ h^{-1}) = \frac{n}{t \times A}$$

The gas products were analyzed by off-line gas chromatography (GC-2014C), combined with thermal conductivity detector (TCD) and two flame ionization detector (FID). TDX-01 packed column was used for gas chromatography, and the carrier gas was high purity argon (99.999%). The gas-phase products were sampled using a gas-tight syringe (Agilent). The molar concentration (C_{gas}) and the volume of gas (V_{gas}) are determined by the microgas injection needle according to the corresponding integrated gas area.

$$FE_{gas} = \frac{Z \times F \times C_{gas} \times V_{gas}}{Q} \times 100\%$$

F: Faraday constant is 96500 C mol^{-1}; Q: the total coulomb amount after electrolysis; Z: The number of transferred electrons required for conversion from OH^- to gaseous products; C_{gas}: gas product concentration measured by gas chromatography, mol mL^{-1}; V_{gas}: Total gas product volume, mL.

Electrochemical active surface area (ECSA) was estimated by double−layer capacitance (C_{dl}) measured with different scan rates from 5 ~ 100 mV s^{-1}. The ECSA of the working electrodes could be calculated according to the following:

$$ECSA = R_f \times S$$

(R_f: roughness factor can be calculated by following equation: $R_f = C_{dl}/a$; S: surface area of the working electrode, 1 cm^2).

Section S5. Attenuated total reflection Fourier transform infrared (ATR-FTIR) spectra ATR-SEIRAS spectra were obtained on a Nicolet iS50 spectrometer in the range of 4000-400 cm^{-1} with 256 scans and a resolution of 4 cm^{-1}. Au film underlayers were pre-deposited onto Si ATR crystal by chemical deposition. The prism was first polished with 0.05 μm Al_2O_3 slurry and sonicated in acetone and water, respectively. Then, the reflecting plane of the prism was immersed in a 40% NH_4F solution for 5 min to create a hydrogen-terminated surface. The prism was then immersed into a mixture of 2% HF and Au plating solution consisting of 5.75 mM $NaAuCl_4 \cdot 2H_2O$, 0.025 M NH_4Cl, 0.025 M $Na_2S_2O_3 \cdot 5H_2O$, 0.075 M Na_2SO_3 and 0.026 M NaOH

at 60°C for 10 min. The obtained Au film was activated with potential scans from -0.2 to 1.5 V_{RHE} in 0.1 M $NaHCO_3$ at a scanning rate of 100 mV s^{-1} to improve the surface enhancement effect. The **[Ni₉]-cluster** and **[Ni₉]-MOF** were prepared by dropping ink onto the activated Au film. The ink was prepared by dispersing 16 mg of **[Ni₉]-cluster** and **[Ni₉]-MOF** powder in 2.5 mL of isopropanol, followed by adding 40 µL of 5% Nafion solution into the mixture. After sonicating for 30 min, 75 µL of the ink was uniformly dropped onto 1 cm^2 of the Au film, followed by drying at room temperature overnight.

A home-designed spectro-electrochemical cell with a three-electrode configuration was employed for the in situ SEIRAS test. Au supported **[Ni₉]-cluster** and **[Ni₉]-MOF** electrode was used as the working electrode, a Platinum wire as the counter electrode and a Hg/HgO electrode as the reference electrode. The cell was integrated into the Nicolet iS50 spectrometer equipped with a liquid-nitrogen-cooled mercury cadmium telluride detector. A 0.5 M KOH solution was used as the electrolyte with cyclohexanol. The background spectrum was collected with a 0.5 M KOH solution and cyclohexanol at open-circuit voltage (OCP) before each test. The spectra were recorded at 1.61 V for different times.

Section S6. Computational details.

Calculations based on first-principles density functional theory (DFT), including molecular dynamics simulations were executed utilizing the Vienna Ab initio Simulation Package (VASP) (1) in conjunction with the Projector Augmented Wave (PAW) methodology.(2) The exchange-correlation functional was managed within the parameters of the Generalized Gradient Approximation (GGA), adopting the Perdew-Burke-Ernzerhof (PBE) functional.(3) The long-range van der Waals interactions are accounted for through the DFT-D3 approach.(4) We implemented a plane wave basis set with an energy cutoff set at 500 eV, and the geometric relaxation was carried through until the forces acting on each atom were less than 0.03 eV/Å. The sampling of the Brillouin zone was conducted using a 1 × 1 × 1 k-point grid. To assure rigorous consistency, calculations were performed until the energy convergence threshold was less than 10^{-5} eV. To effectively isolate periodic structures and preclude their interaction, a vacuum buffer of 15 Å was inserted along the z-axis.

The free energy of the intermediates is calculated:

$$\Delta G = \Delta E_{DFT} + \Delta ZPE - T\Delta S$$

where ΔE_{DFT}, ΔZPE and ΔS are the changes of the reaction energy obtained from DFT calculations, zero-point energy, and the changes of entropy from the initial state to the final state, respectively. T is temperature and the T of 298.15 K was used in all computations.

Figures

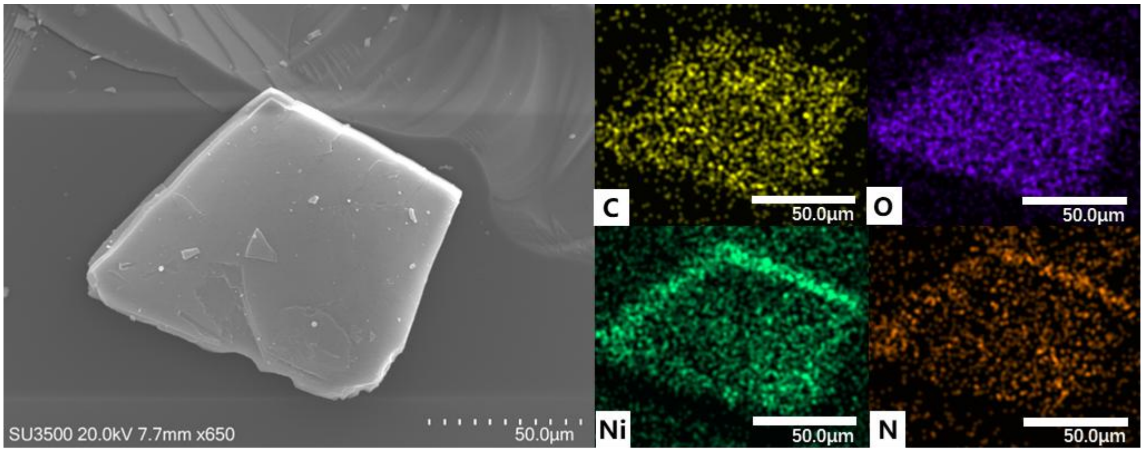

Fig. S1. The mapping results of **[Ni_9]-MOF** by scanning electron microscopy (SEM), showing the uniform distribution of the four elements-Ni, C, N, O on the material surface.

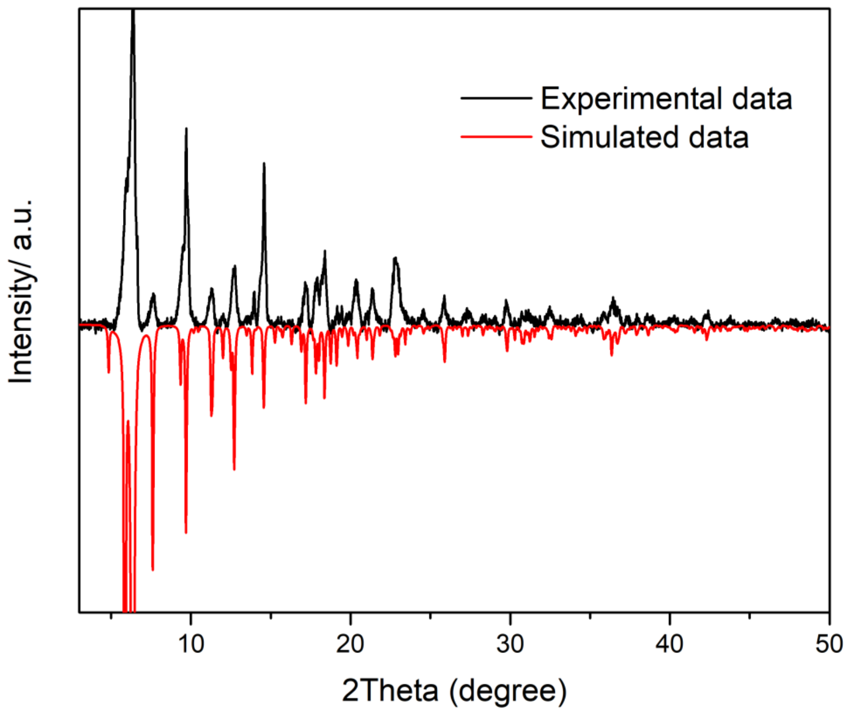

Fig. S2. PXRD Patterns of as-synthesized **[Ni$_9$]-MOF** and simulated data based on the single-crystal data via Mercury.

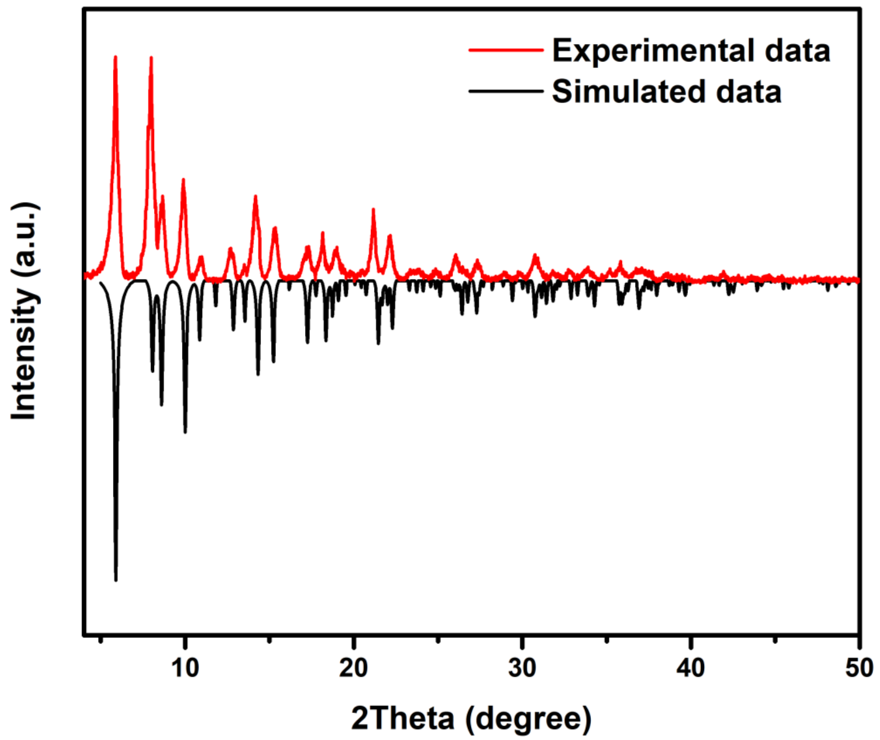

Fig. S3. PXRD Patterns of as-synthesized **[Ni$_9$]-cluster** and simulated data based on the single-crystal data via Mercury.

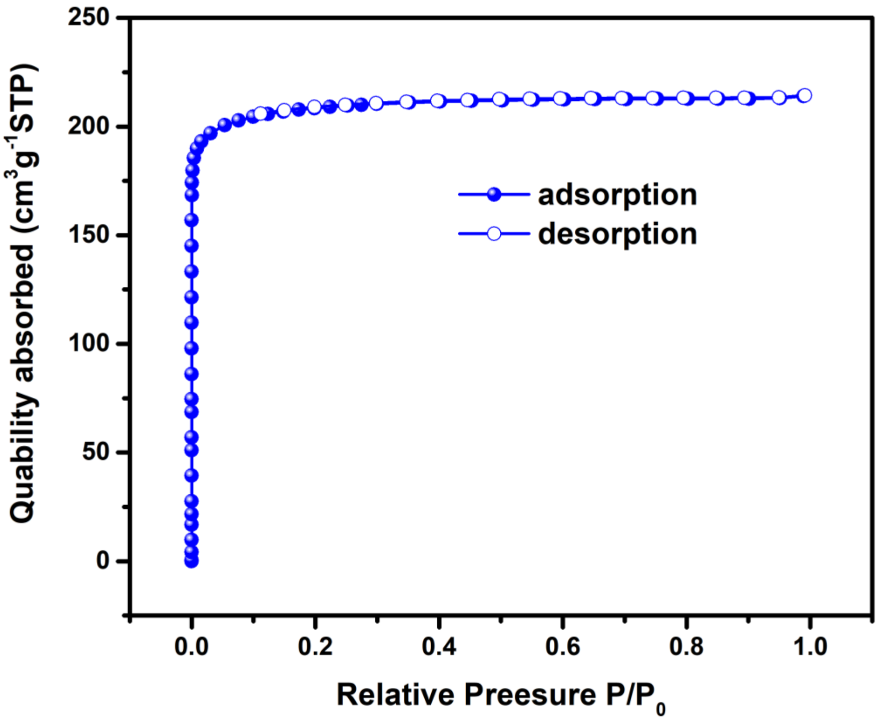

Fig. S4. N_2 adsorption/desorption of **[Ni$_9$]-MOF** (77 K).

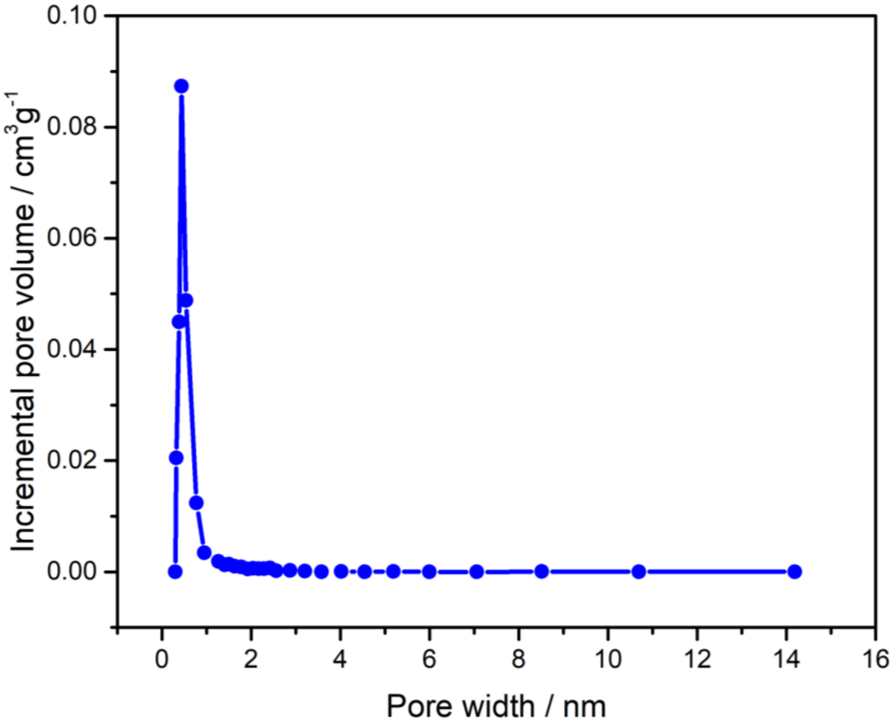

Fig. S5. The pore diameter of **[Ni₉]-MOF** was determined using density functional theory (DFT)-based pore volume analysis as part of this study.

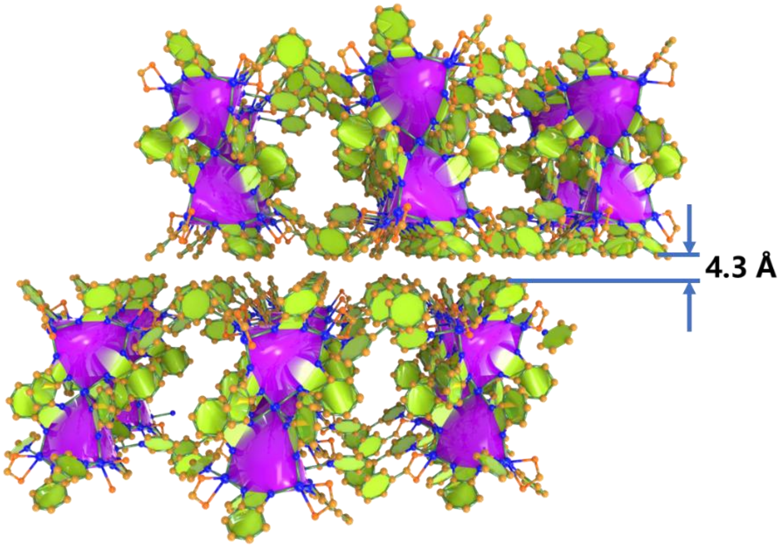

Fig. S6. Lamellar structure of **[Ni_9]-MOF.**

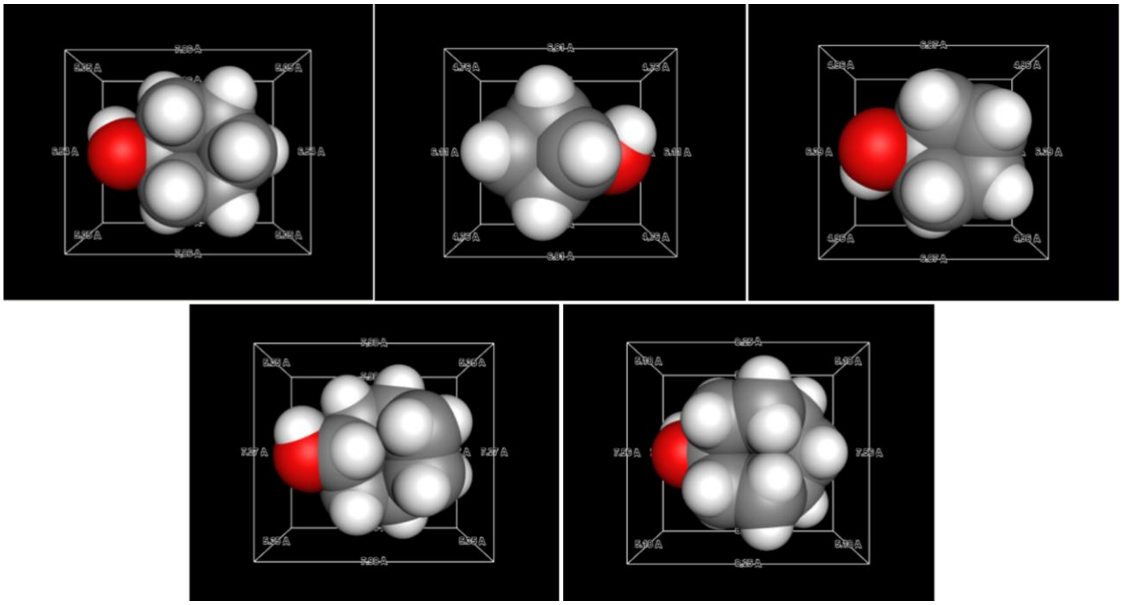

Fig. S7. The three-dimensional sizes of cyclobutanol (6.81 × 6.11 × 4.76 Å^3), cyclopentanol (6.87 × 6.39 × 4.96 Å^3), cyclohexanol (7.86 × 6.54 × 5.05 Å^3), cycloheptanol (7.98 × 7.27 × 5.35 Å^3), and cyclooctanol (8.25 × 7.56 × 5.18 Å^3).

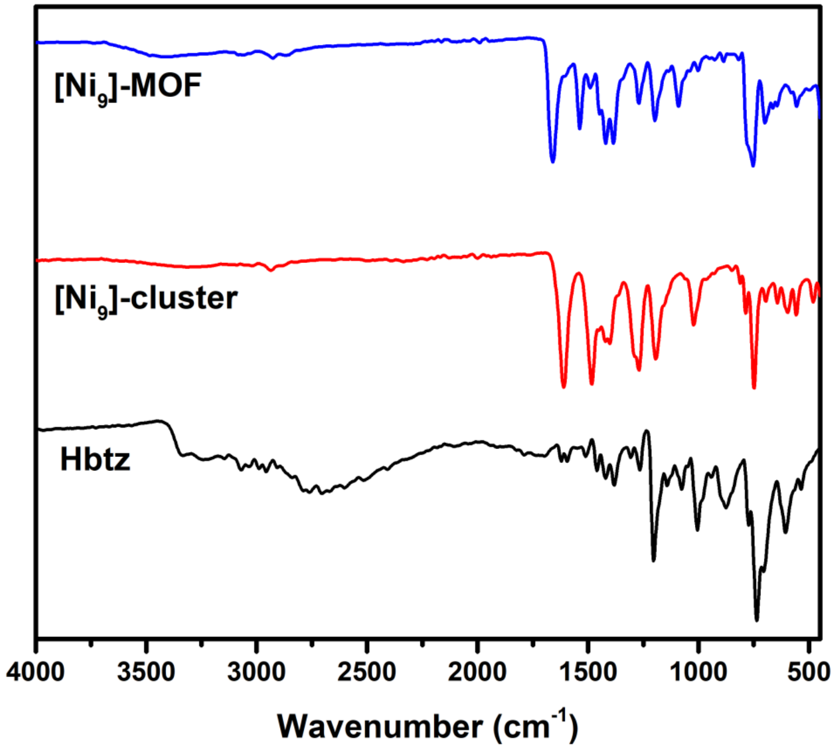

Fig. S8. IR spectra of **Hbtz**, **[Ni9]-cluster** and **[Ni9]-MOF**.

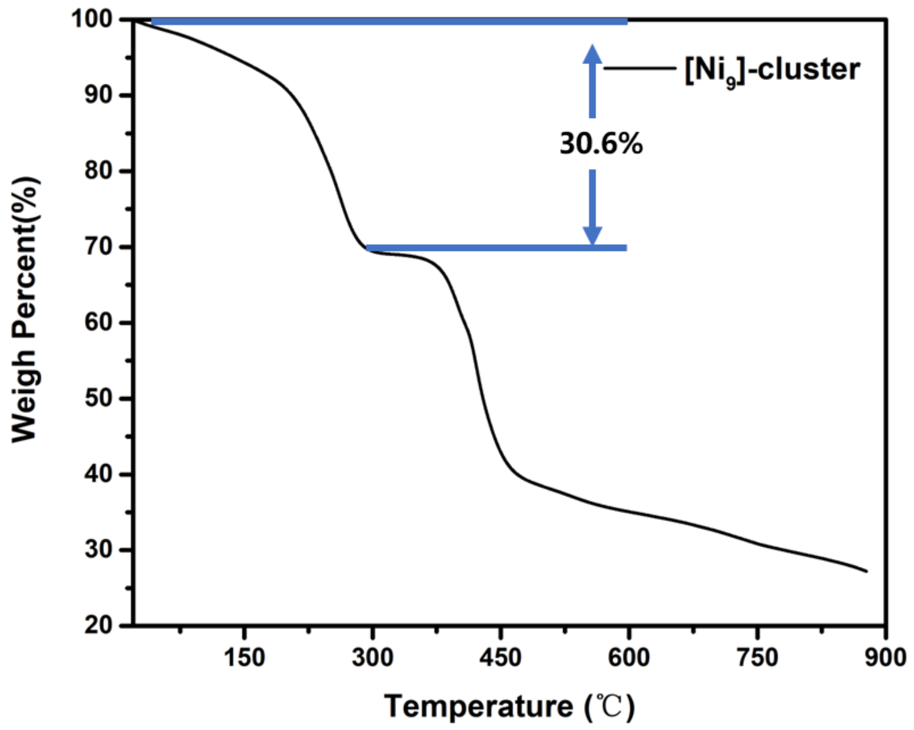

Fig. S9. TG curves of **[Ni$_9$]-cluster**.

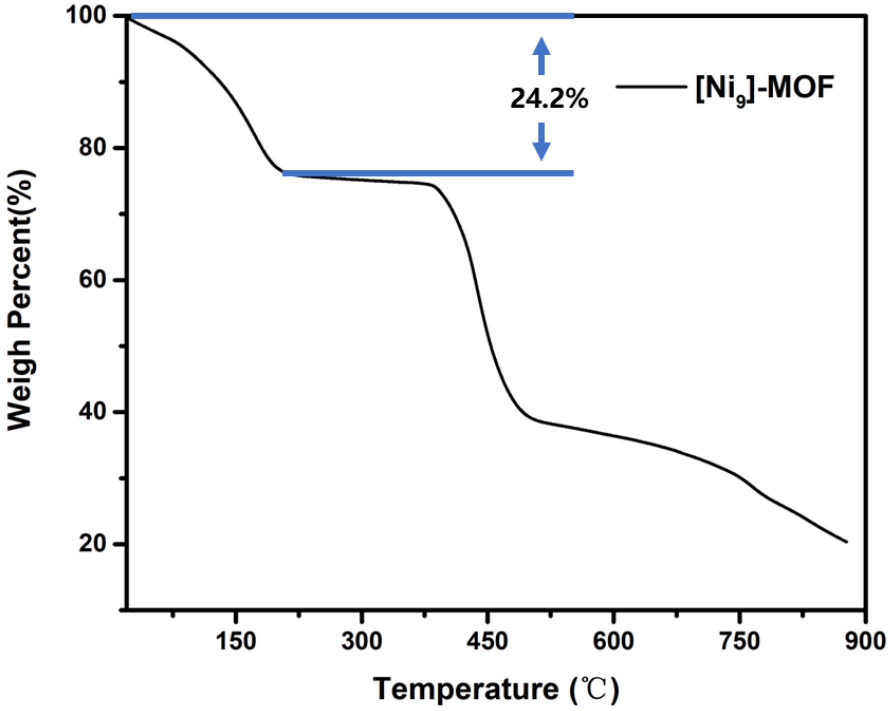

Fig. S10. TG curves of **[Ni9]-MOF**.

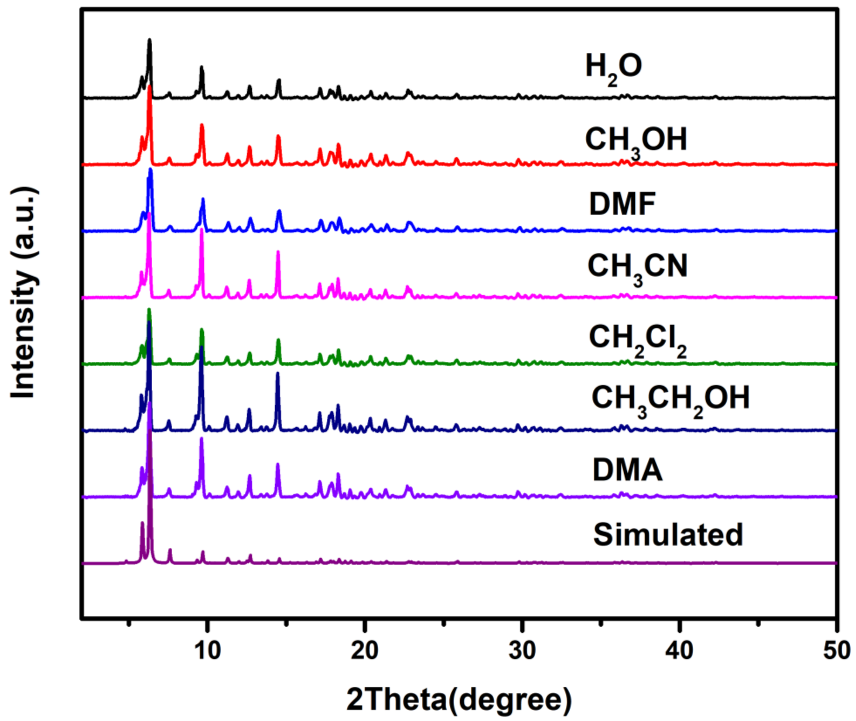

Fig. S11. PXRD patterns of **[Ni₉]-MOF** after immersing in common organic solvents for 12 h. Typically, 50 mg of **[Ni₉]-MOF** were immersed in 20 mL corresponding solvents respectively and sealed for 12 h. The processed samples were collected and washed by centrifugation. After drying in vacuum oven at 80 °C overnight, the samples were ground before PXRD test.

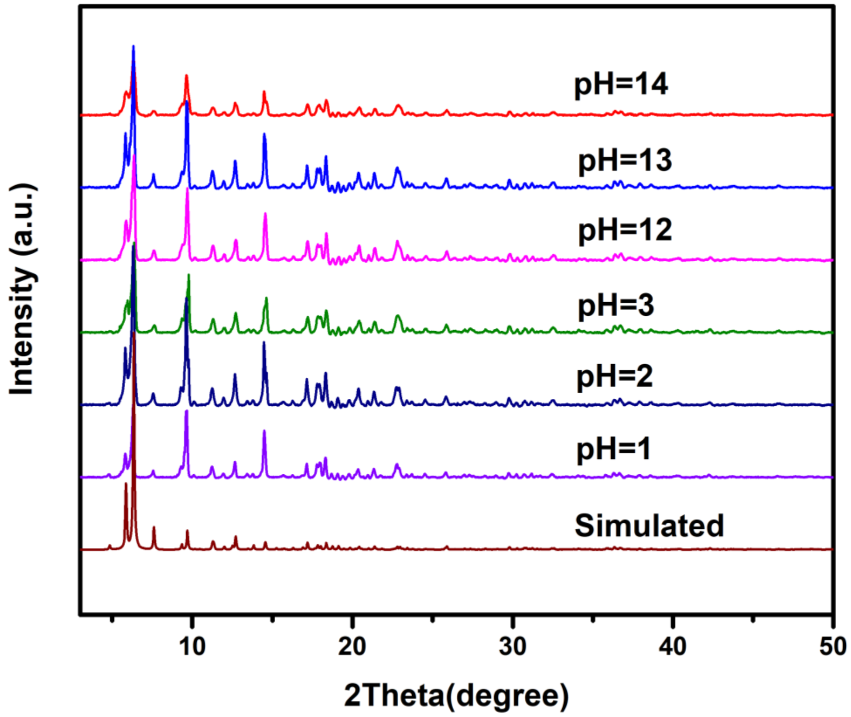

Fig. S12. PXRD patterns of **[Ni$_9$]-MOF** after immersing in aqueous solvents with different pH for 24 h. Typically, 50 mg of **[Ni$_9$]-MOF** were immersed in 20 mL corresponding aqueous solvents respectively and change the solution every three hours. After 24 h, the processed samples were collected and washed by centrifugation. The samples were dried in vacuum oven at 80 °C overnight and ground before PXRD test. The solution used in the experiment is adjusted to pH value by HCl and NaOH.

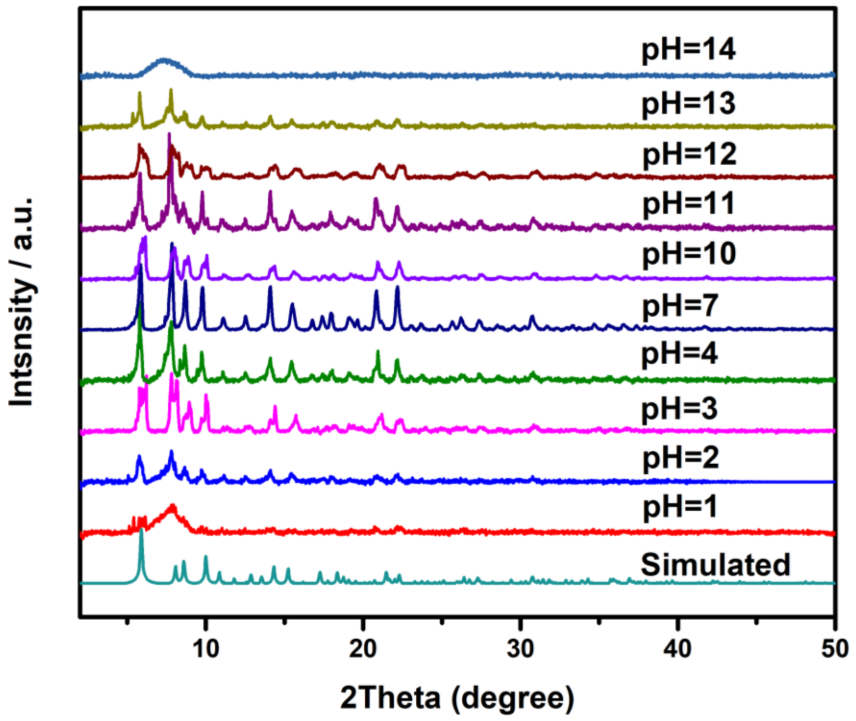

Fig. S13. PXRD patterns of **[Ni9]-cluster** after immersing in aqueous solvents with different pH for 24 h. Typically, 50 mg of **[Ni9]-cluster** were immersed in 20 mL corresponding aqueous solvents respectively and change the solution every three hours. After 24 h, the processed samples were collected and washed by centrifugation. The samples were dried in vacuum oven at 80 °C overnight and ground before PXRD test. The solution used in the experiment is adjusted to pH value by HCl and NaOH.

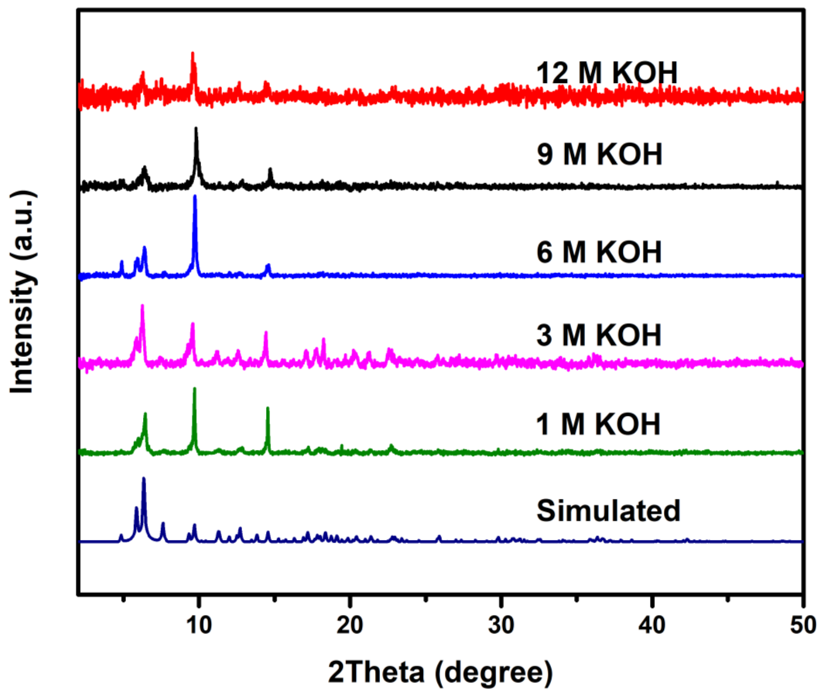

Fig. S14. The PXRD spectra of **[Ni$_9$]-MOF** after immersing in a series of KOH solutions with concentrations from 1 to 12 M for 24 h.

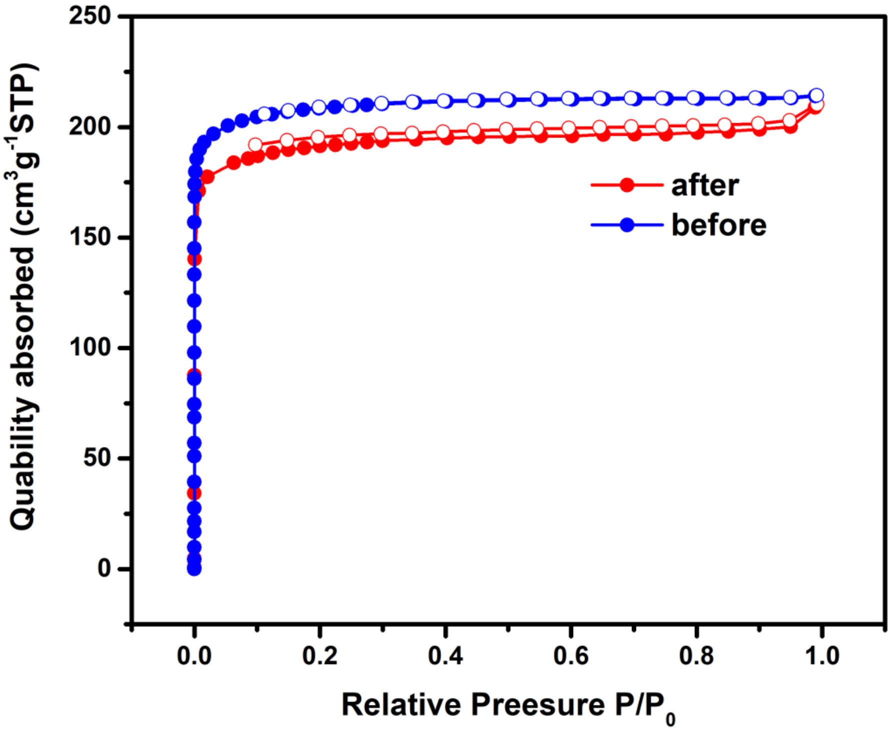

Fig. S15. N_2 adsorption/desorption of **[Ni$_9$]-MOF** (77 K) before and after the base stability tests.

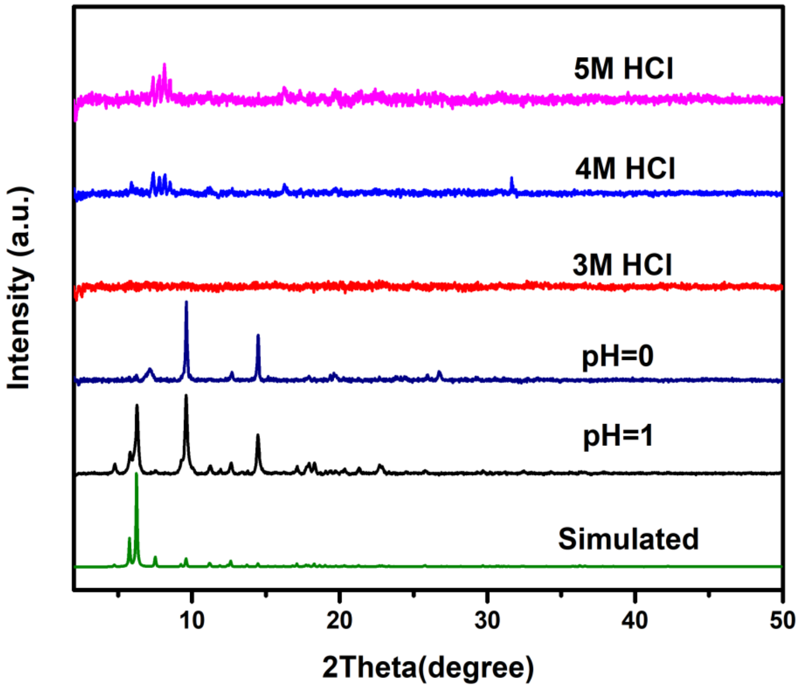

Fig. S16. PXRD patterns of **[Ni9]-MOF** after immersing in aqueous solvents with different concentrations of hydrochloric acid (HCl) for 24 h. Typically, 50 mg of **[Ni9]-MOF** were immersed in 20 mL corresponding aqueous solvents respectively and change the solution every three hours. After 24 h, the processed samples were collected and washed by centrifugation. The samples were dried in vacuum oven at 80 °C overnight and ground before PXRD test. The solution used in the experiment is adjusted to pH value by HCl.

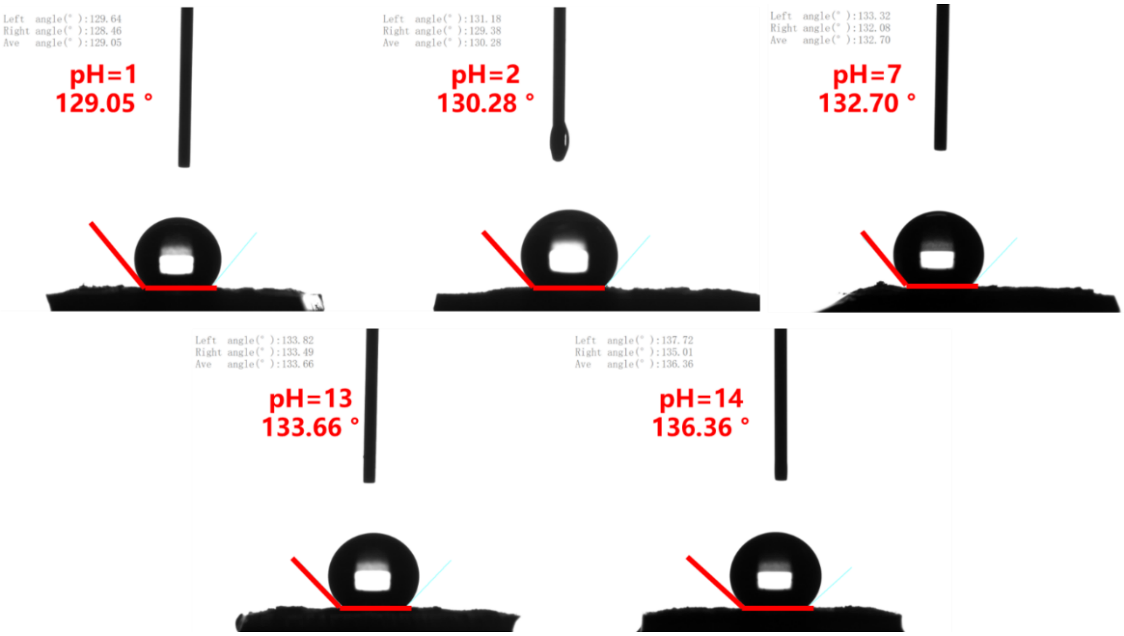

Fig. S17. The contact angle of **$[Ni_9]$-MOF** samples immersed in aqueous solutions across varying pH levels was analyzed. The results demonstrate that the MOF exhibits enhanced hydrophilic behavior in acidic solutions (pH < 7) compared to alkaline conditions (pH > 7), suggesting that reduced hydrophilicity in alkaline environments contributes to its structural stability under such conditions.

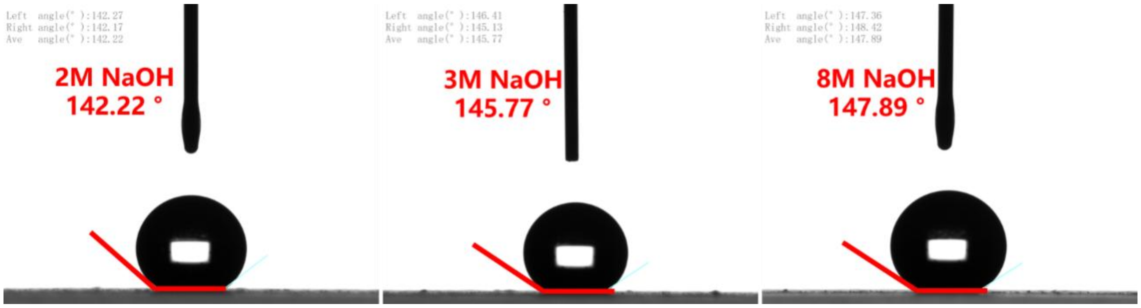

Fig. S18. To verify its hydrophobicity in higher concentrations of alkali, the contact angle of **[Ni_9]-MOF** samples immersed in aqueous solutions across NaOH (2 M, 3 M, 8 M) was analyzed.

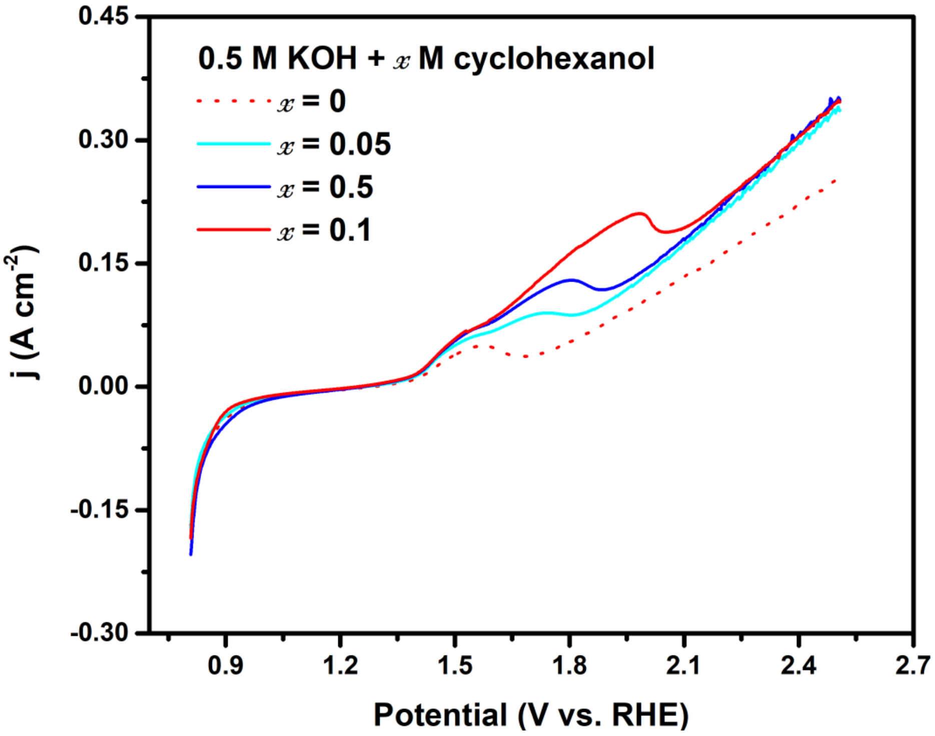

Fig. S19. LSV curves of **[Ni₉]-MOF** in 0.5 M KOH containing different concentrations cyclohexanol.

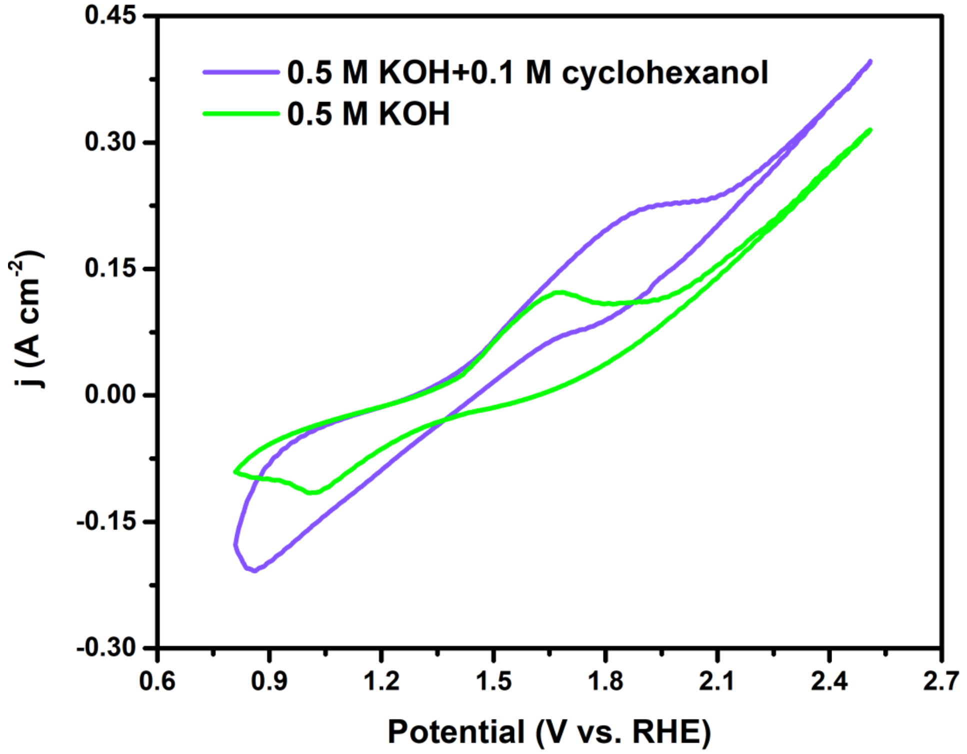

Fig. S20. CV curves of **[Ni$_9$]-MOF** in 0.5 M KOH with and without 0.1 M cyclohexanol in Ni foam.

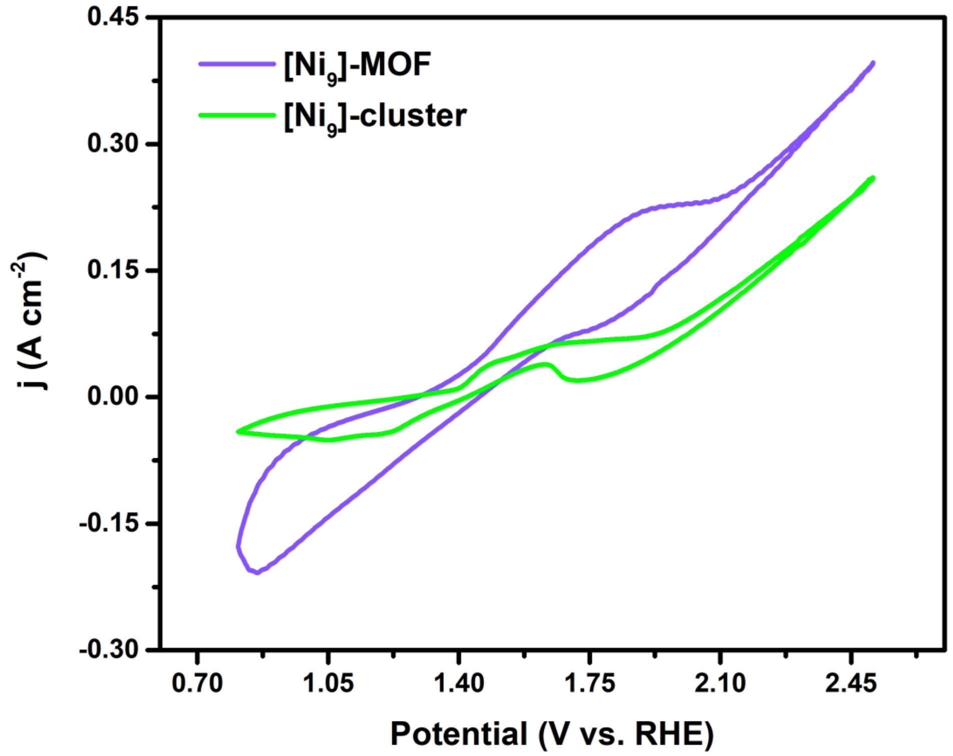

Fig. S21. CV curves of **[Ni₉]-MOF** and **[Ni₉]-cluster** in 0.5 M KOH with 0.1 M cyclohexanol at a scan rate of 10 mV s^{-1}.

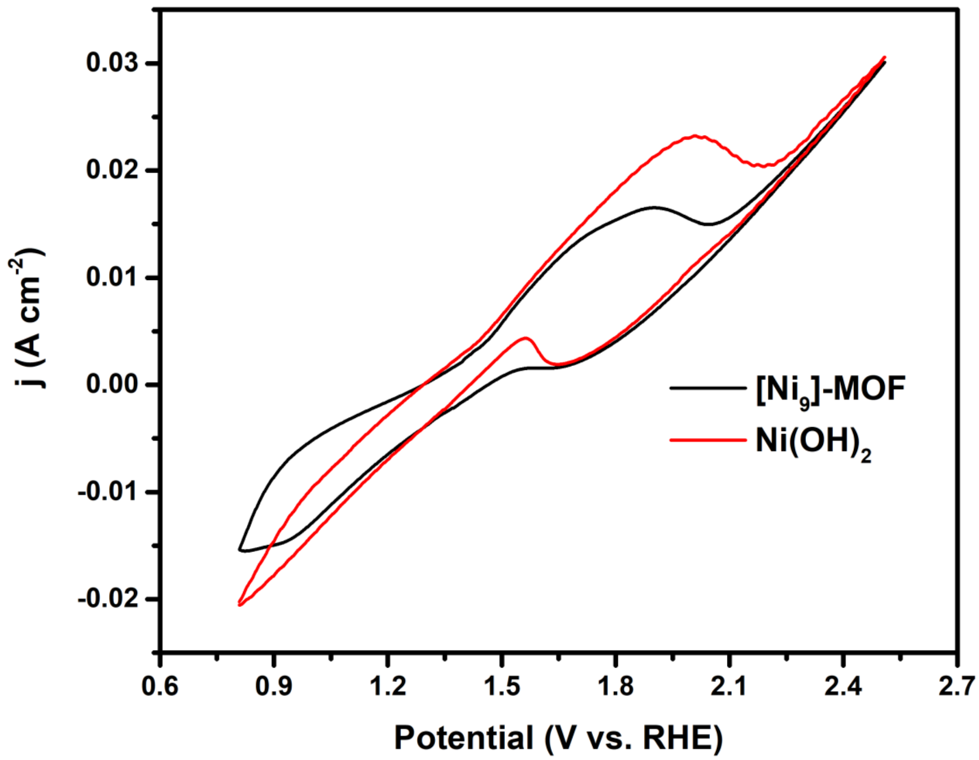

Fig. S22. CV curves of **[Ni₉]-MOF** and $Ni(OH)_2$ in 0.5 M KOH with 0.1 M cyclohexanol on carbon paper.

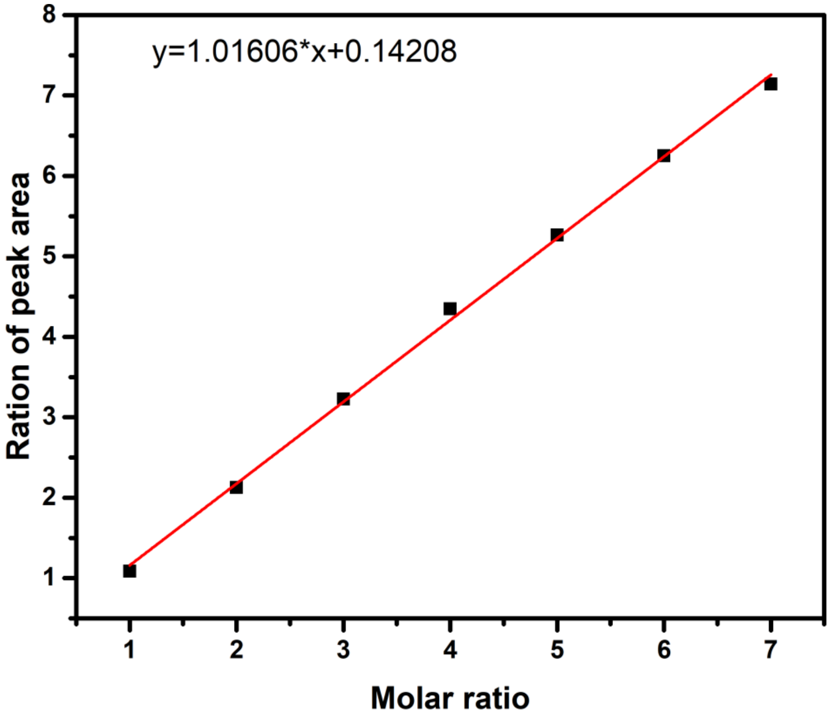

Fig. S23. The correct calibration curve of AA was obtained by adding DMSO as the internal standard for the ^{1}H-NMR tests.

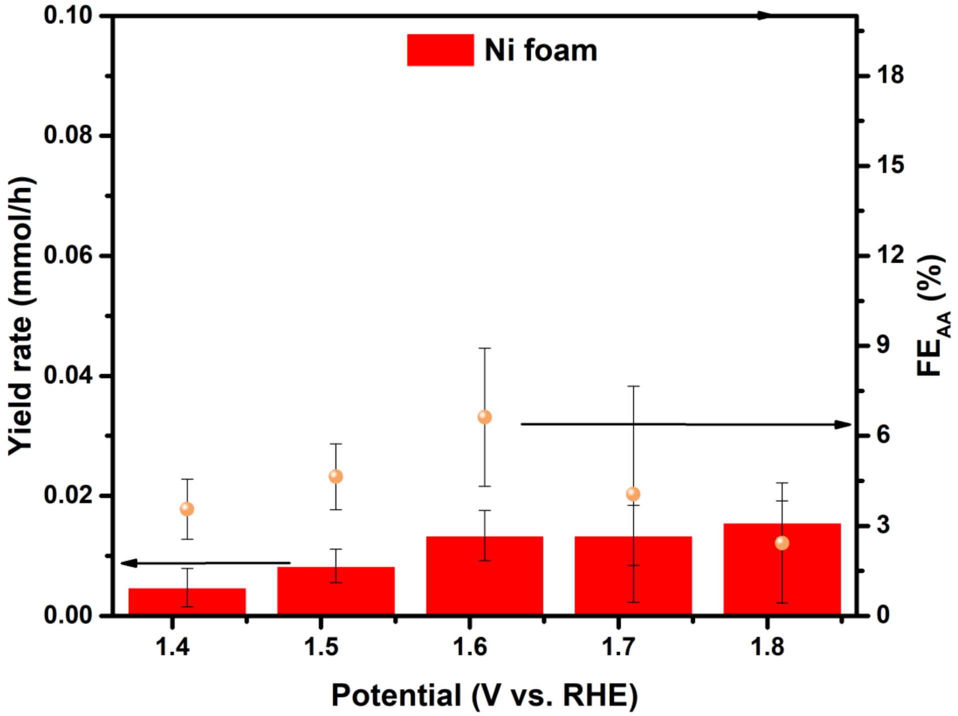

Fig. S24. Faraday efficiency and yield of electrosynthesis of AA using Ni foam.

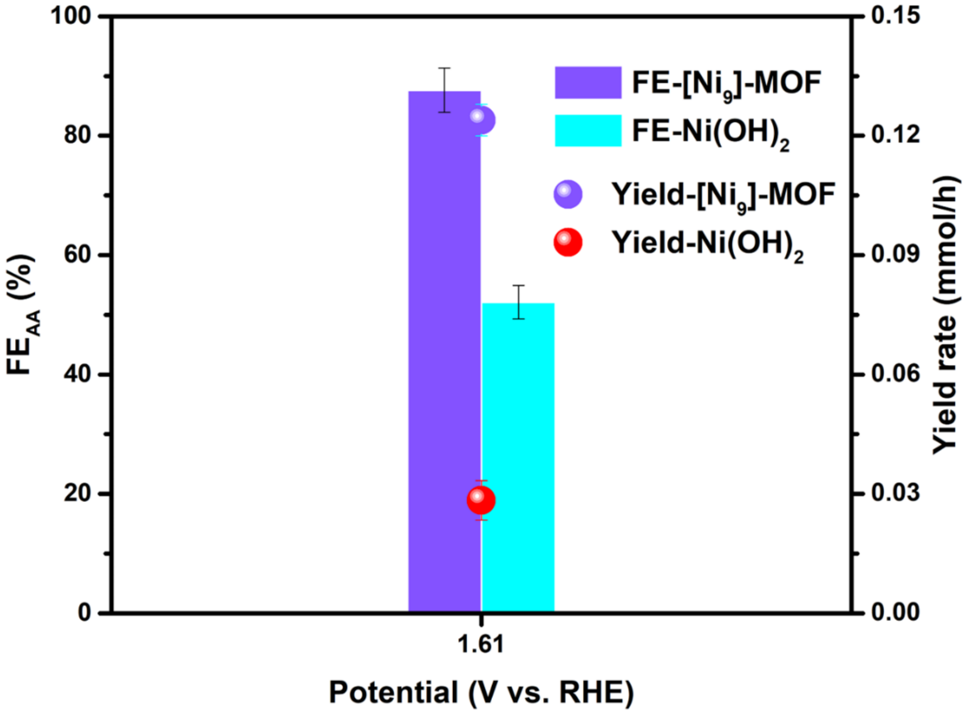

Fig. S25. Faraday efficiency and yield of electrosynthesis of AA using **[Ni$_9$]-MOF** and $Ni(OH)_2$.

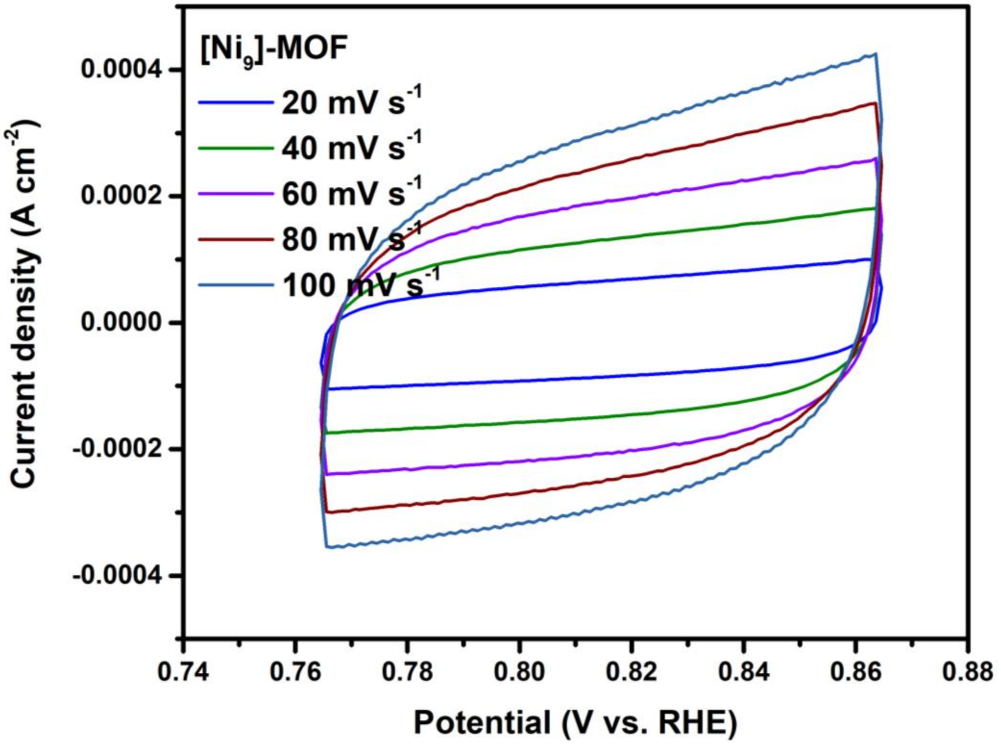

Fig. S26. Measured CVs of **[Ni$_9$]-MOF** in 0.5 M KOH with 0.1 M cyclohexanol electrolyte with a potential range from 0.76 V to 0.88 V.

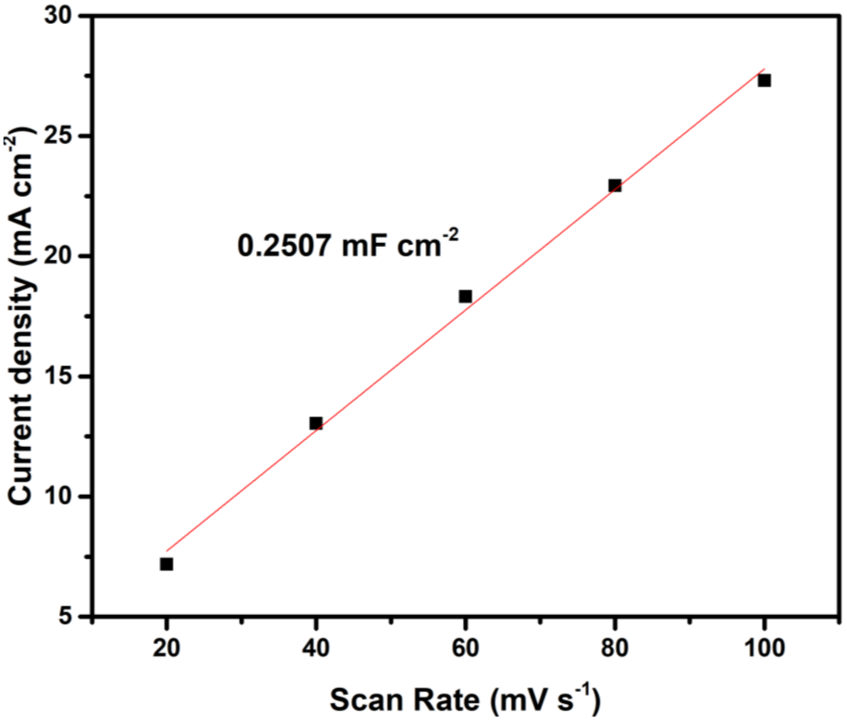

Fig. S27. Charging current density differences plotted against the scan rates on for the calculation of ECSA for **[Ni_9]-MOF.**

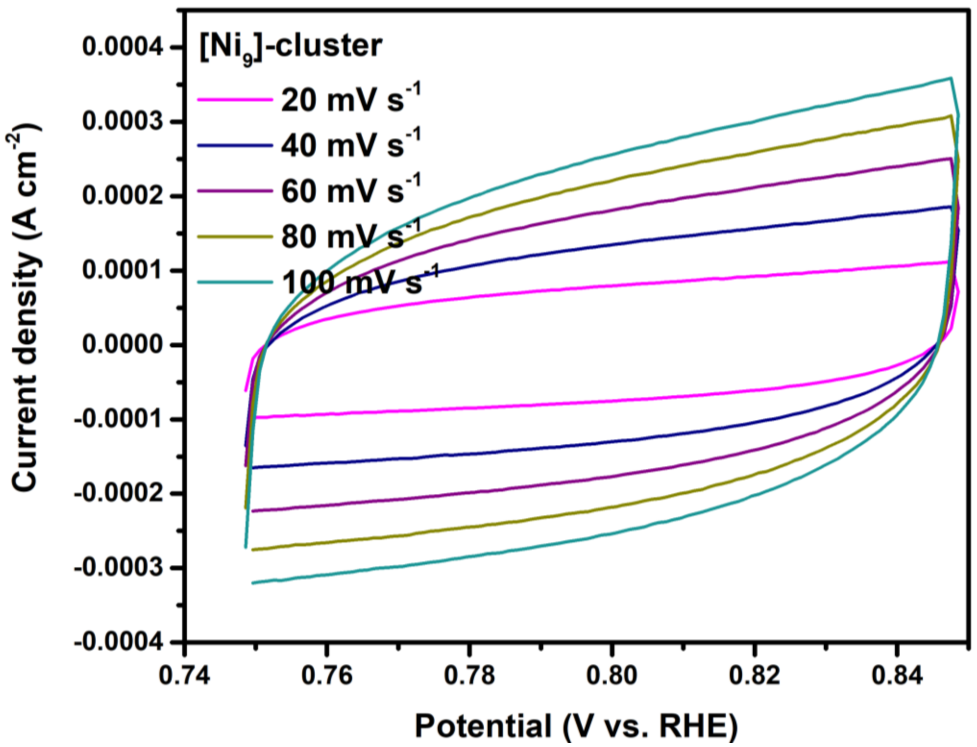

Fig. S28. Measured CVs of **[Ni_9]-cluster** in 0.5 M KOH with 0.1 M cyclohexanol electrolyte with a potential range from 0.75 V to 0.85 V.

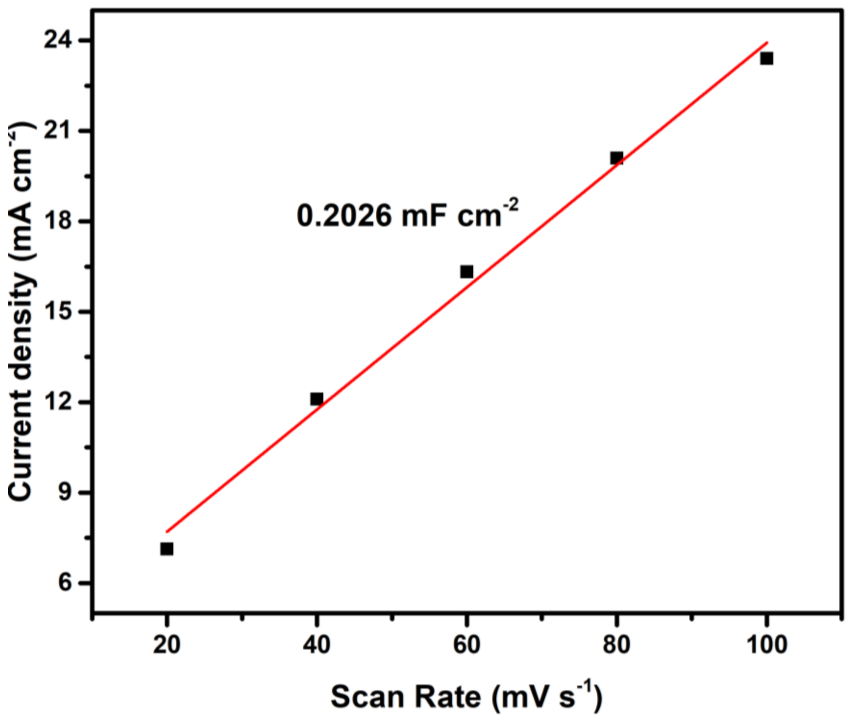

Fig. S29. Charging current density differences plotted against the scan rates on for the calculation of ECSA for **[Ni_9]-cluster**.

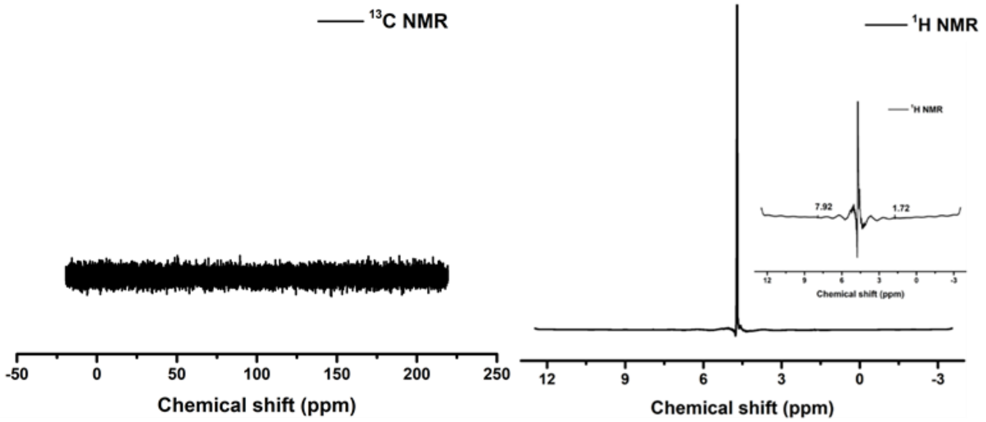

Fig. S30. ^{13}C NMR and ^{1}H NMR spectrum measured from the supernatant after immersing **[Ni$_9$]-MOF** in 8 M KOH solution for 24 hours.

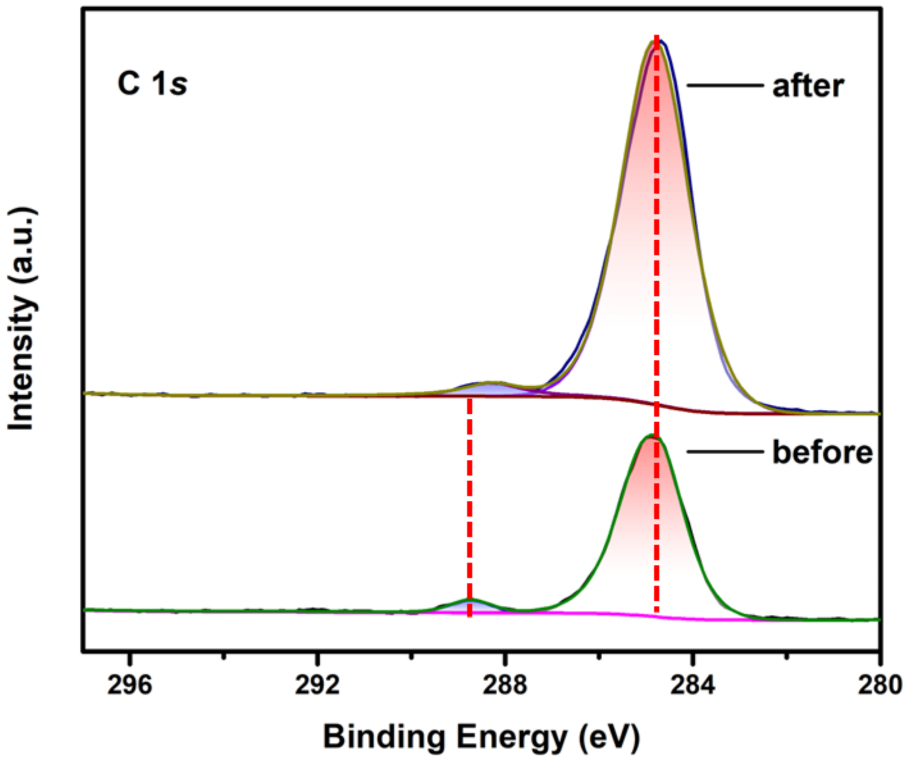

Fig. S31. Comparison of XPS spectra for **[Ni$_9$]-MOF** materials before and after reaction: C 1*s*.

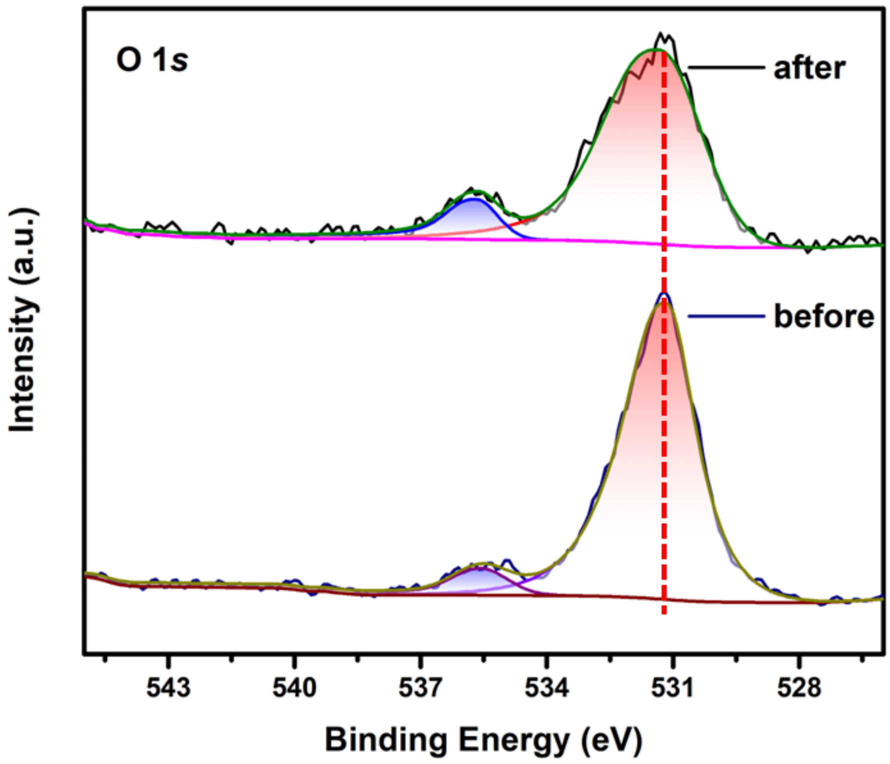

Fig. S32. Comparison of XPS spectra for **[Ni9]-MOF** materials before and after reaction: O 1*s*.

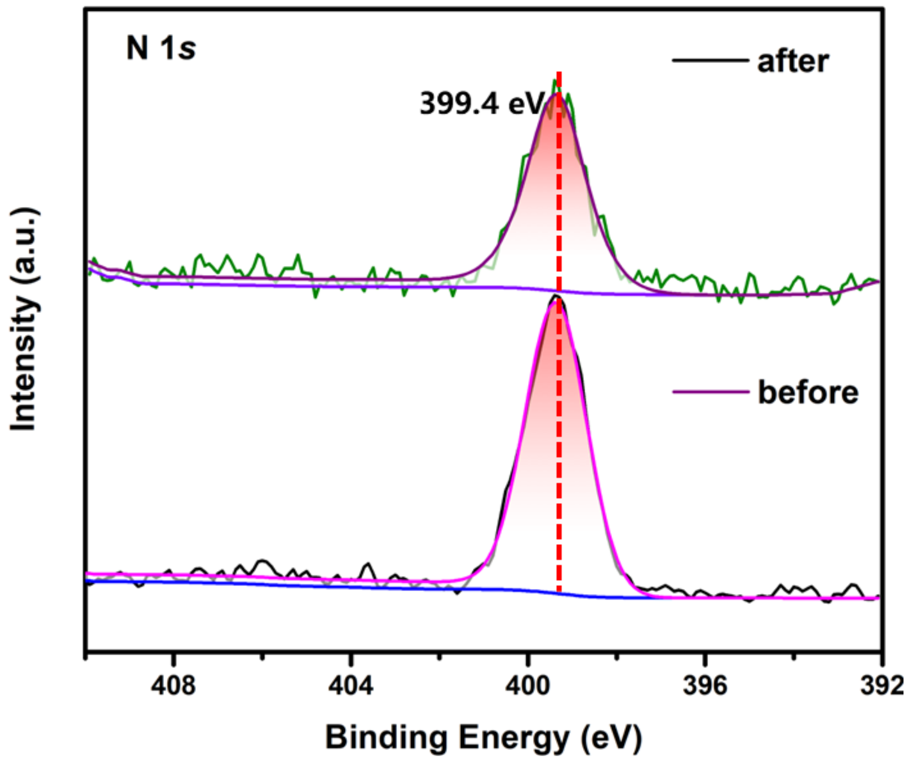

Fig. S33. Comparison of XPS spectra for **[Ni$_9$]-MOF** materials before and after reaction: N 1*s*.

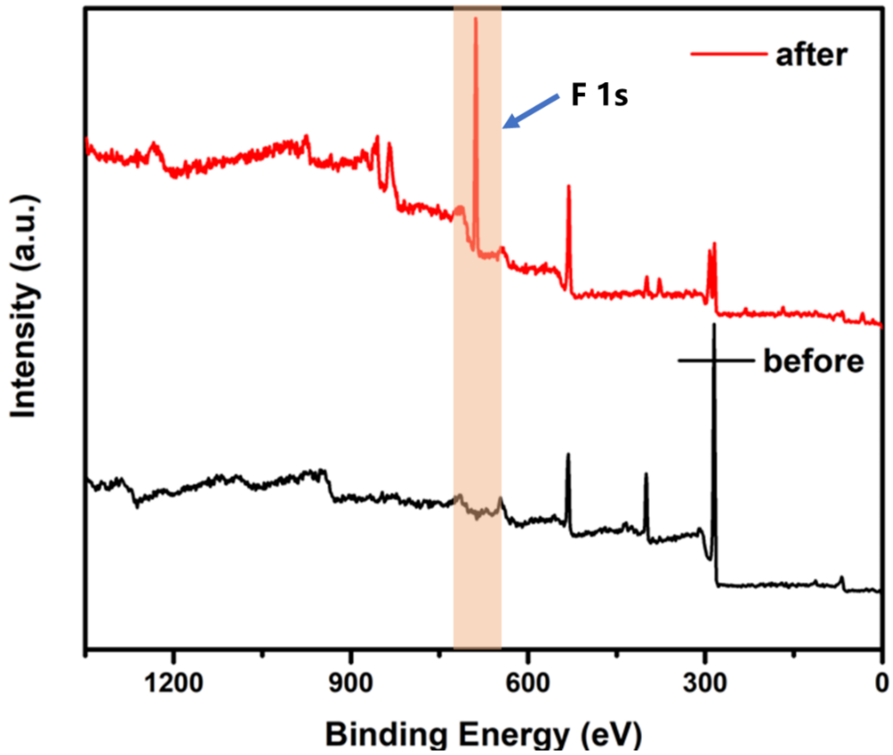

Fig. S34. Comparison of XPS spectra for **[Ni$_9$]-MOF** materials before and after reaction. The peak at 689 eV corresponds to the F 1s binding energy of perfluoro(4-methyl-3,6-dioxa-7-octene) sulfonyl fluoride present in 5 wt% Nafion solution.

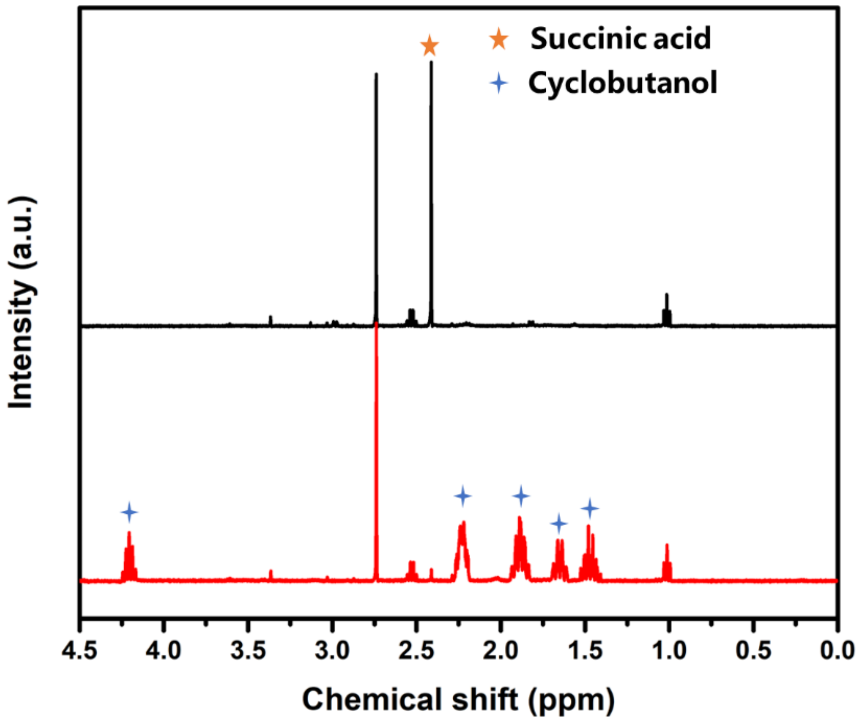

Fig. S35. The catalytic reaction employing cyclobutanol as the starting material yielded succinic acid as the primary product. The ^{1}H NMR spectrum of the reaction products obtained over the **[Ni9]-MOF** catalyst in 0.5 M KOH confirmed the structural identity of succinic acid.

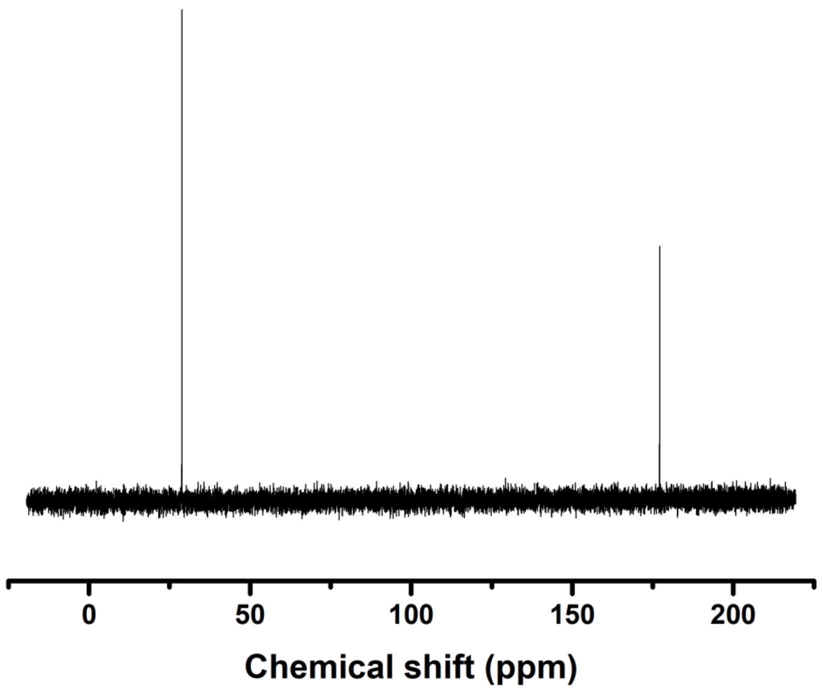

Fig. S36. The ^{13}C nuclear magnetic resonance (NMR) spectrum of purified succinic acid was recorded.

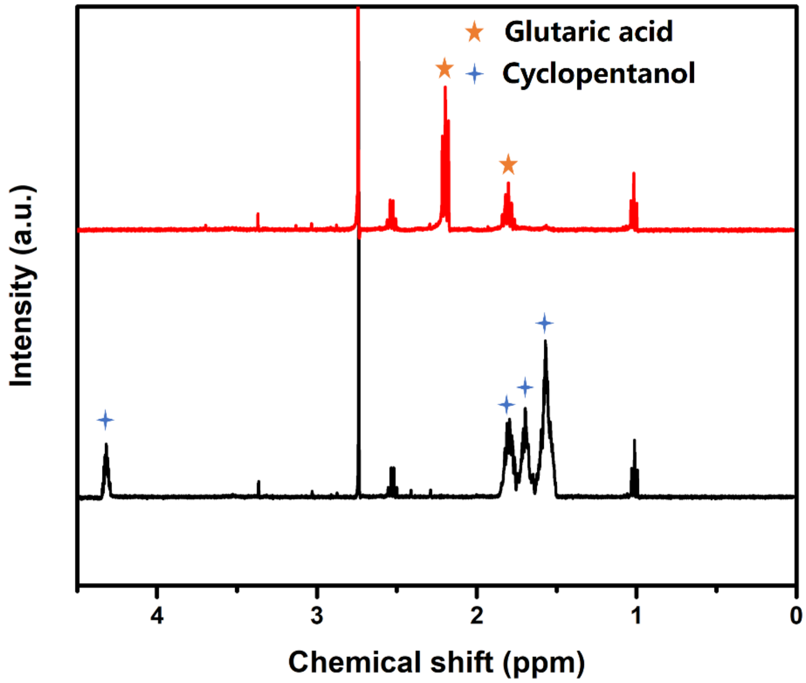

Fig. S37. The catalytic reaction employing cyclopentanol as the starting material yielded Glutaric acid as the primary product. The ^{1}H NMR spectrum of the reaction products obtained over the **[Ni9]-MOF** catalyst in 0.5 M KOH confirmed the structural identity of Glutaric acid.

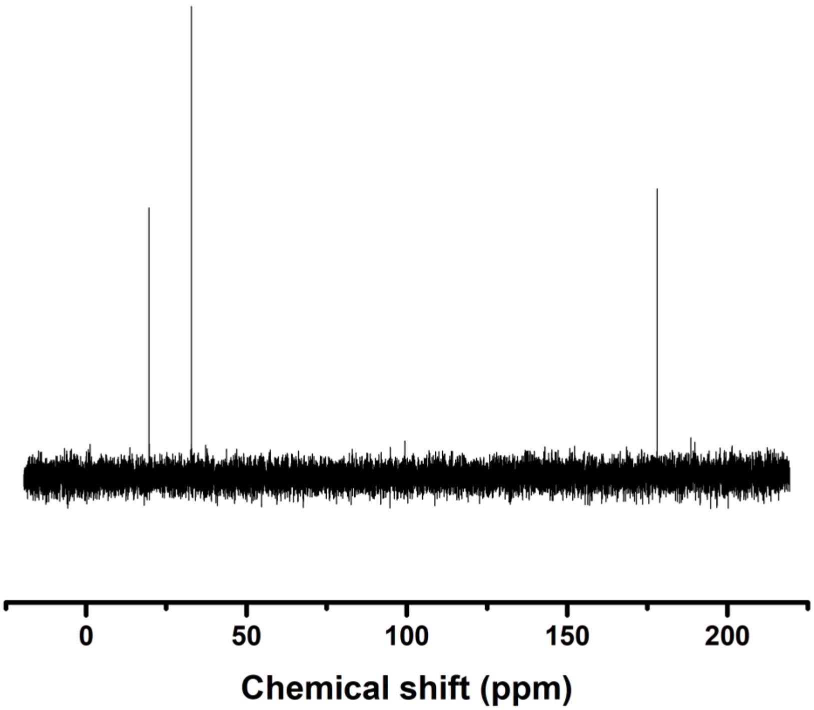

Fig. S38. The ^{13}C nuclear magnetic resonance (NMR) spectrum of purified Glutaric acid was recorded.

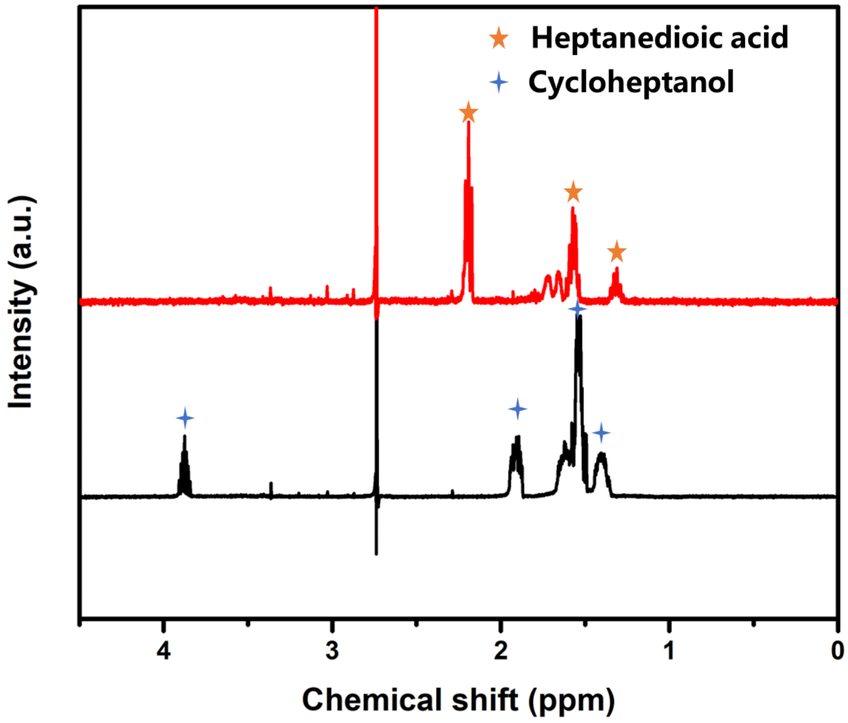

Fig. S39. The catalytic reaction employing cycloheptanol as the starting material yielded heptanedioic acid as the primary product. The ^{1}H NMR spectrum of the reaction products obtained over the **[Ni$_9$]-MOF** catalyst in 0.5 M KOH confirmed the structural identity of heptanedioic acid.

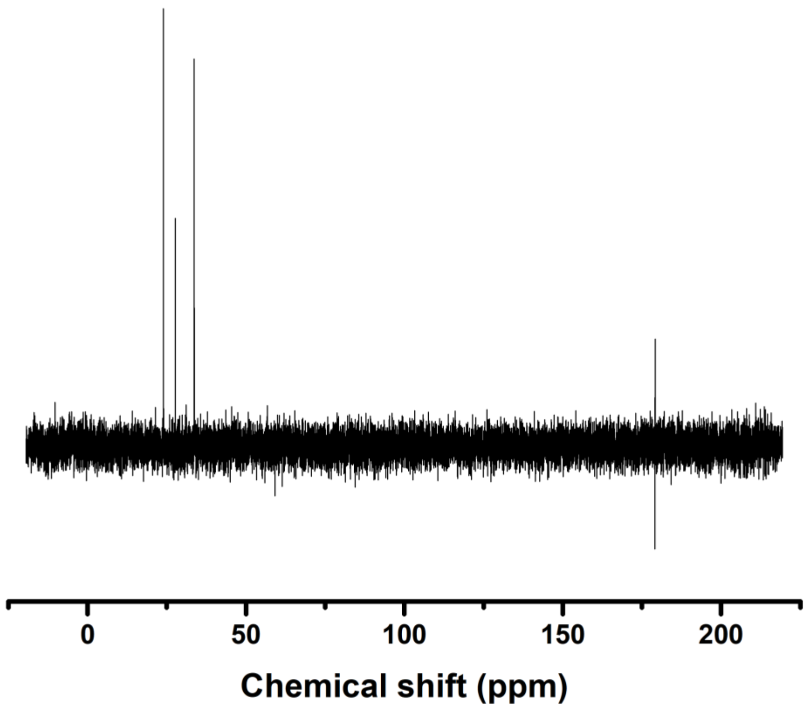

Fig. S40. The ^{13}C nuclear magnetic resonance (NMR) spectrum of purified heptanedioic acid was recorded.

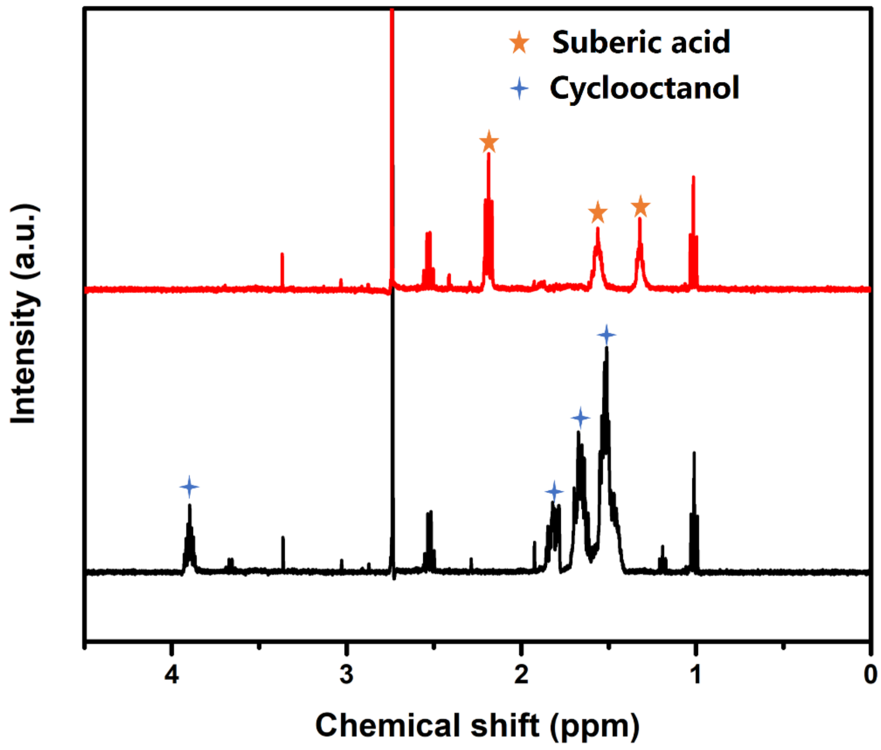

Fig. S41. The catalytic reaction employing cyclooctanol as the starting material yielded suberic acid as the primary product. The ^{1}H NMR spectrum of the reaction products obtained over the **[Ni$_9$]-MOF** catalyst in 0.5 M KOH confirmed the structural identity of suberic acid.

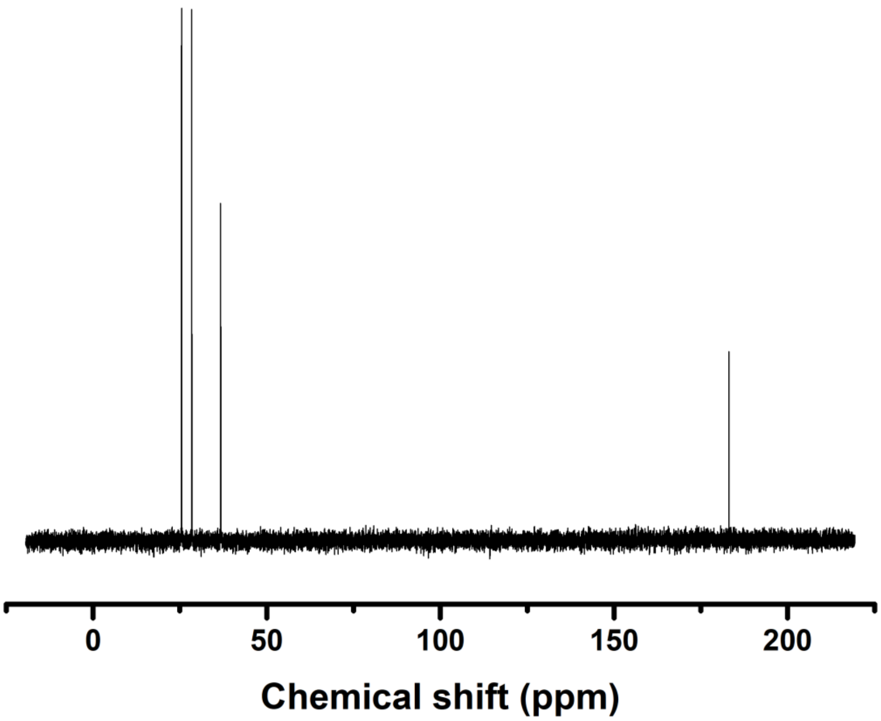

Fig. S42. The ^{13}C nuclear magnetic resonance (NMR) spectrum of purified Suberic acid was recorded.

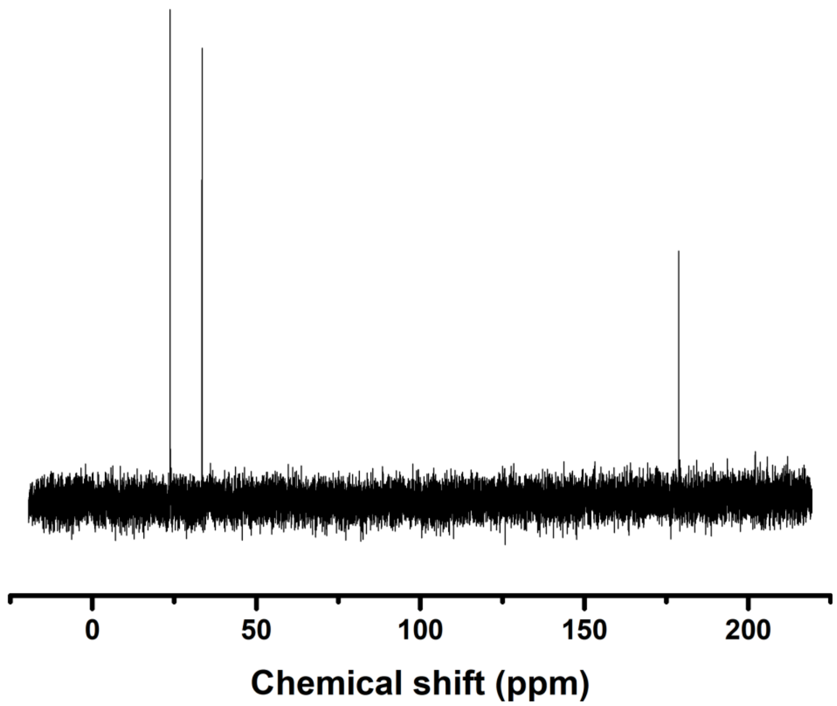

Fig. S43. The ^{13}C nuclear magnetic resonance (NMR) spectrum of purified adipic acid was recorded.

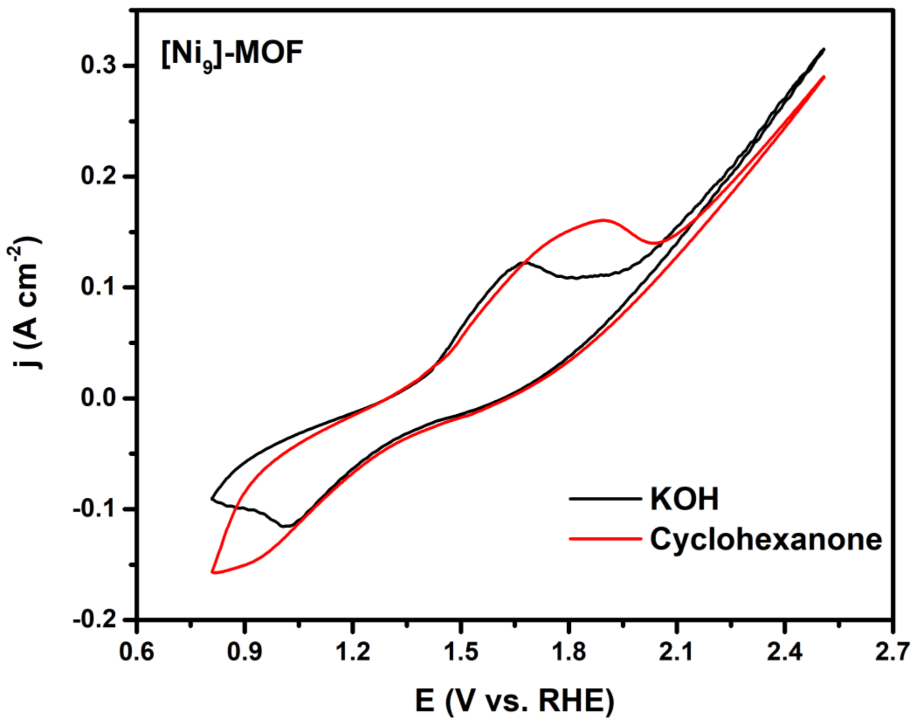

Fig. S44. CV curves of **[Ni9]-MOF** in 0.5 M KOH electrolyte were recorded at a scan rate of 10 mV s^{-1}, both in the absence and presence of cyclohexanone.

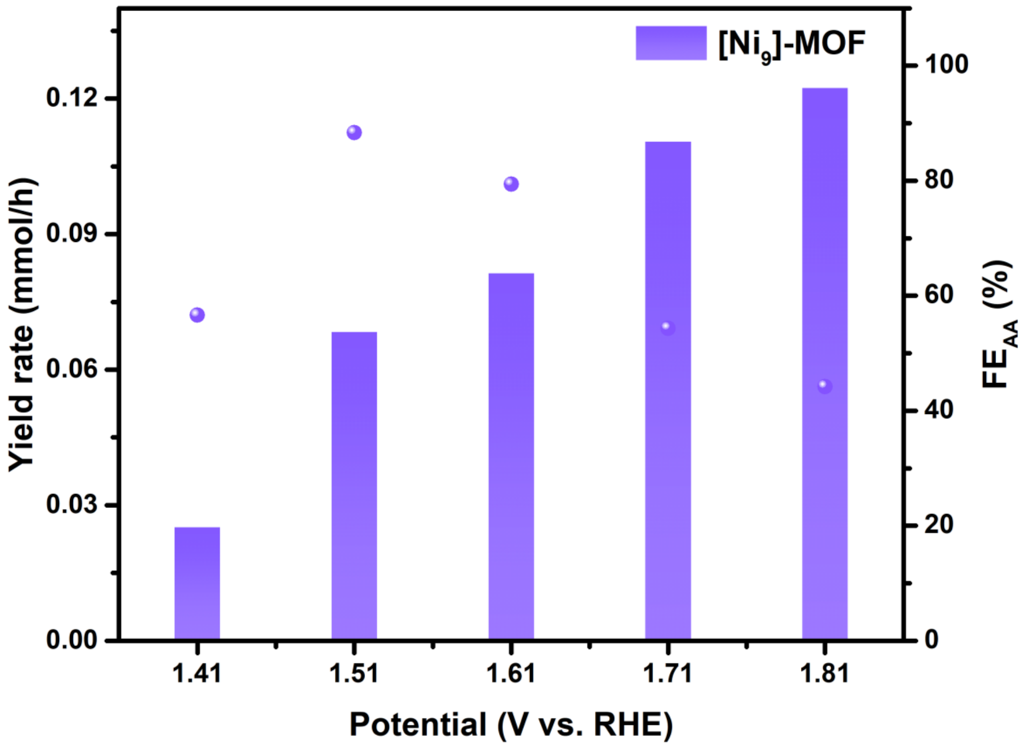

Fig. S45. Faradaic efficiency and yield of adipic acid from cyclohexanone at different potentials.

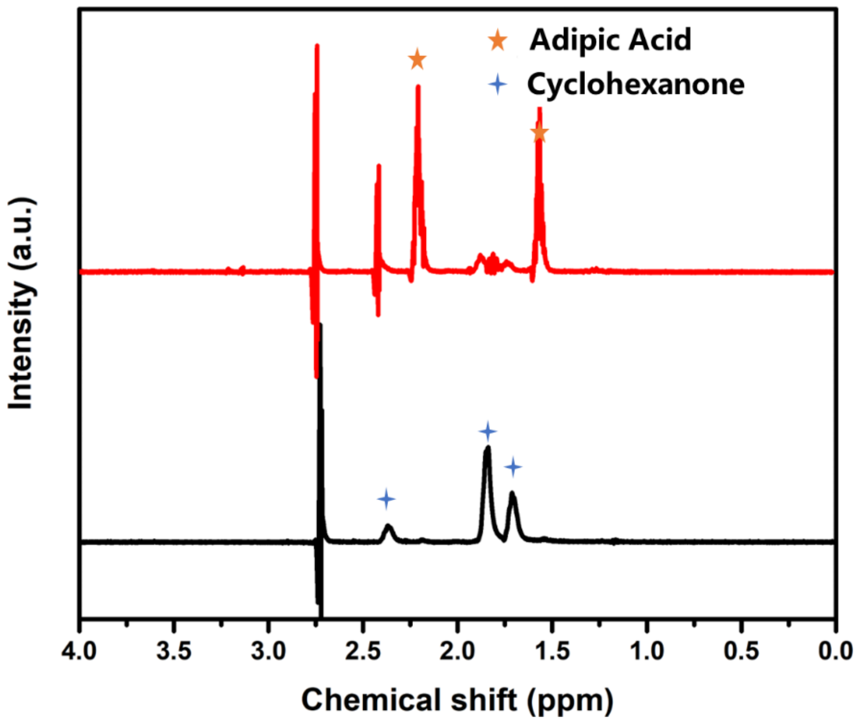

Fig. S46. ^{1}H-NMR spectrum of products over **[Ni$_9$]-MOF** in 0.5 M KOH with cyclohexanone as the substrate.

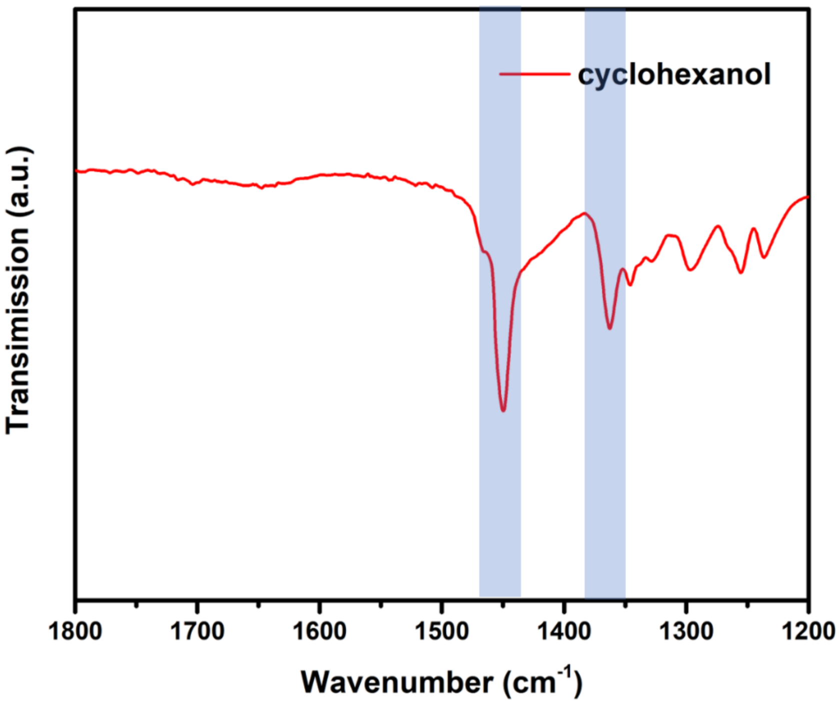

Fig. S47. FTIR spectrum of cyclohexanol as the standard sample.

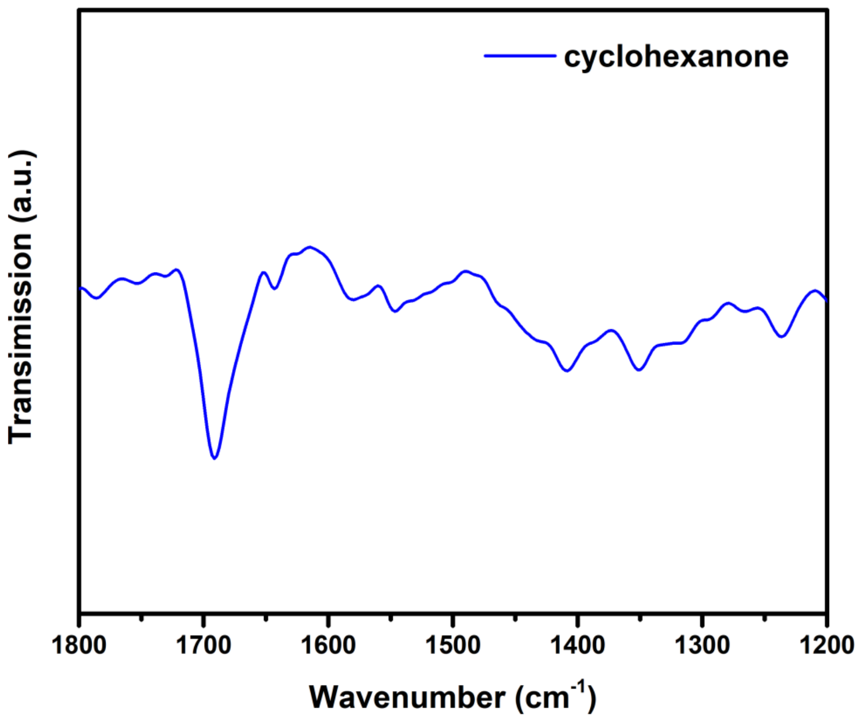

Fig. S48. FTIR spectrum of cyclohexanone as the standard sample.

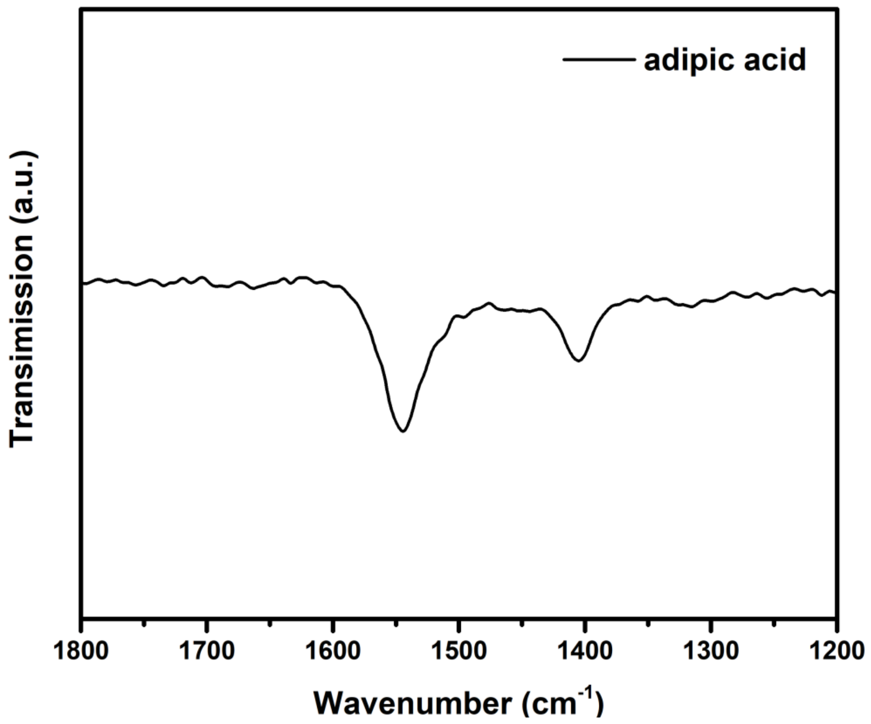

Fig. S49. FTIR spectrum of adipic acid as the standard sample.

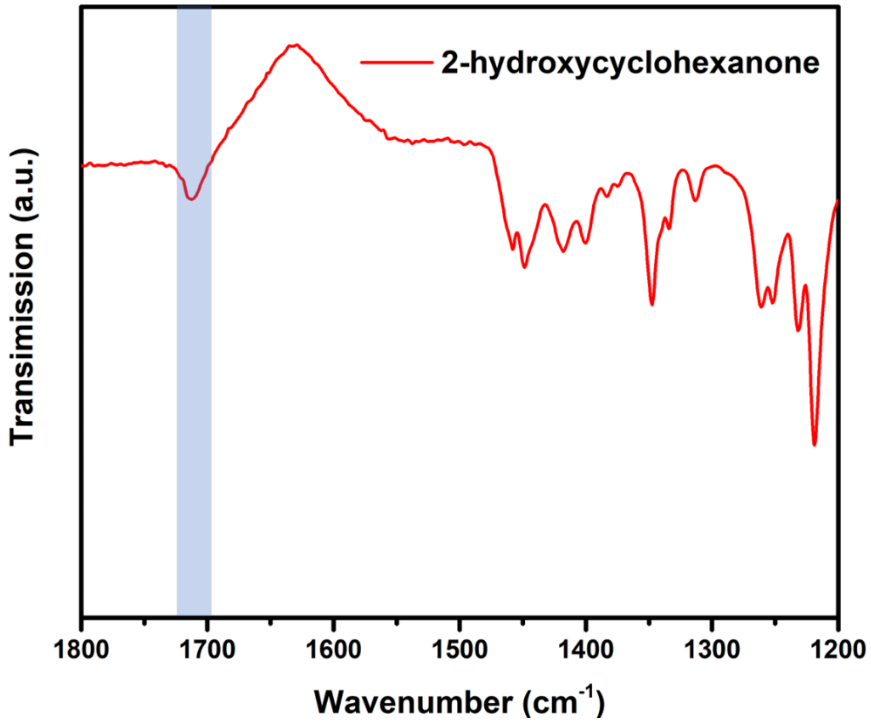

Fig. S50. FTIR spectrum of 2-hydroxycyclohexanone as the standard sample.

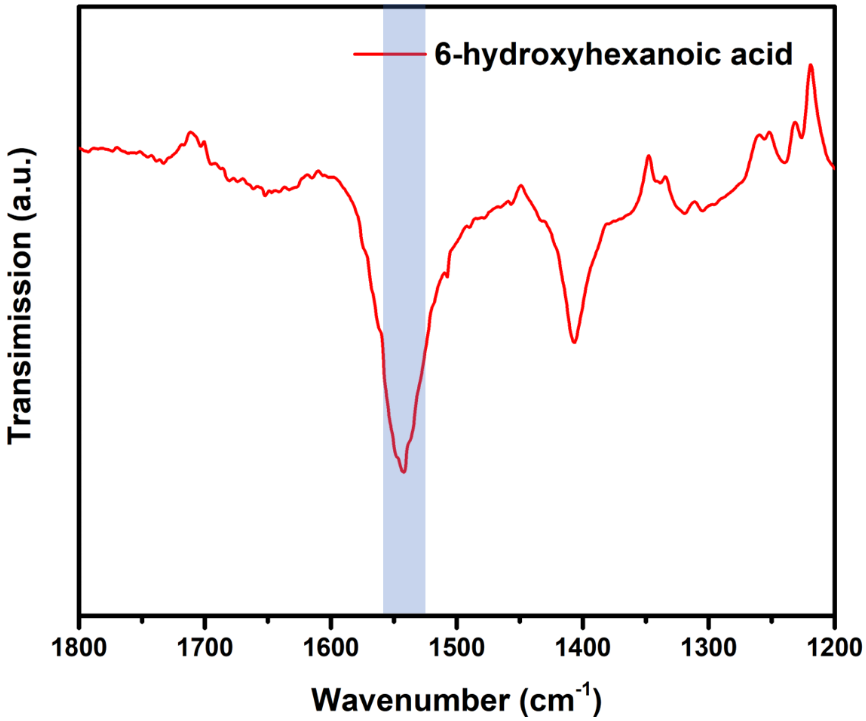

Fig. S51. FTIR spectrum of 6-hydroxyhexanoic acid as the standard sample.

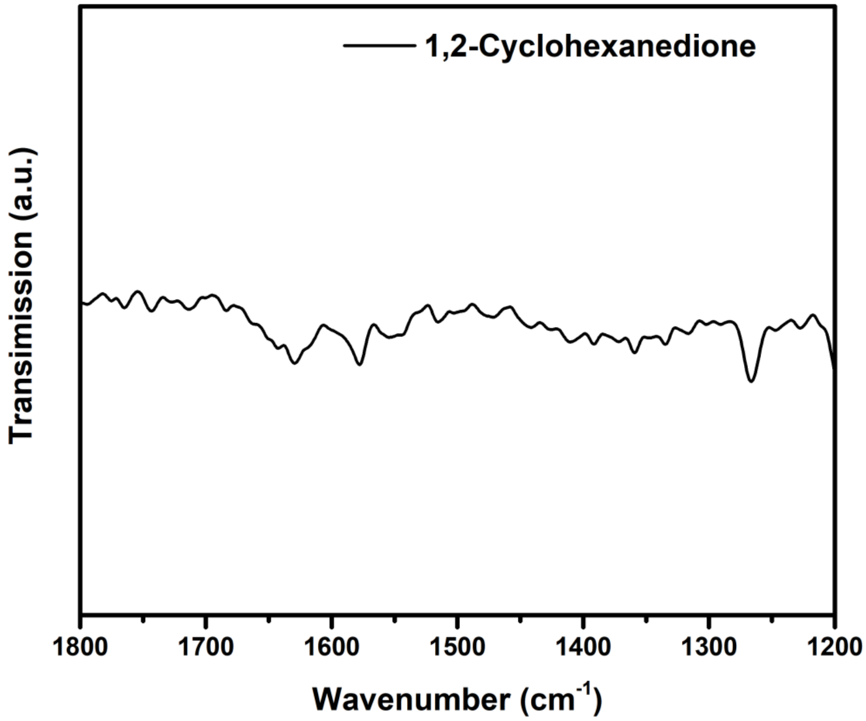

Fig. S52. FTIR spectrum of 1,2-cyclohexanedione as the standard sample.

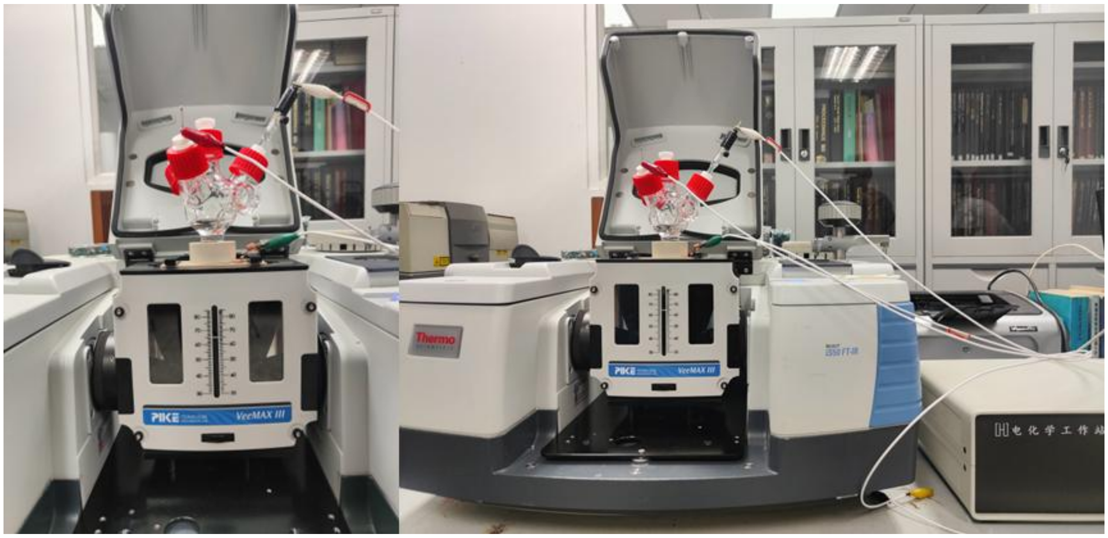

Fig. S53. In situ testing diagram of ATR-FTIR.

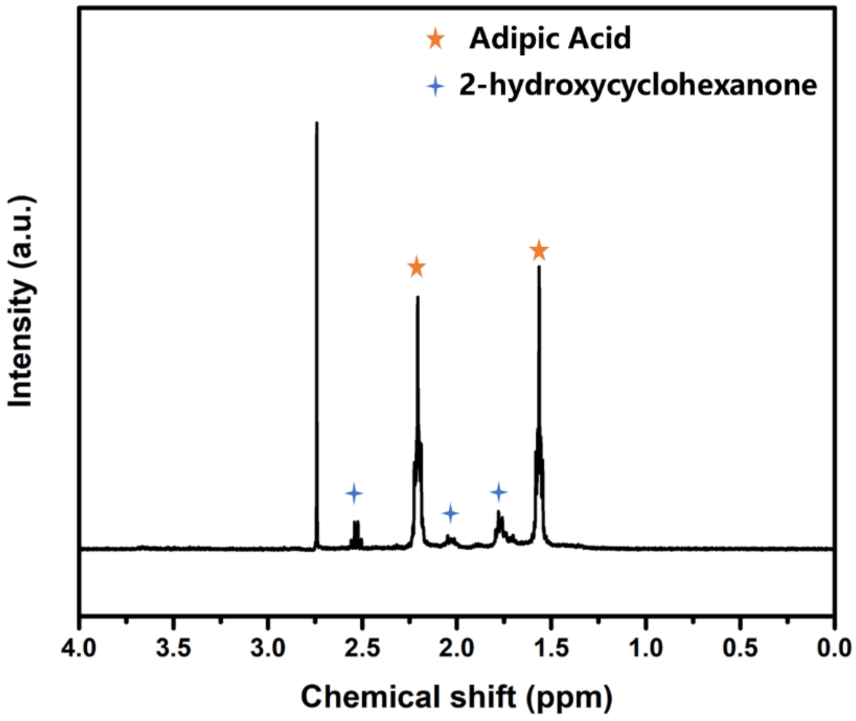

Fig. S54. ^{1}H-NMR spectrum of products over **[Ni$_9$]-MOF** in 0.5 M KOH with 2-hydroxycyclohexanone as the substrate.

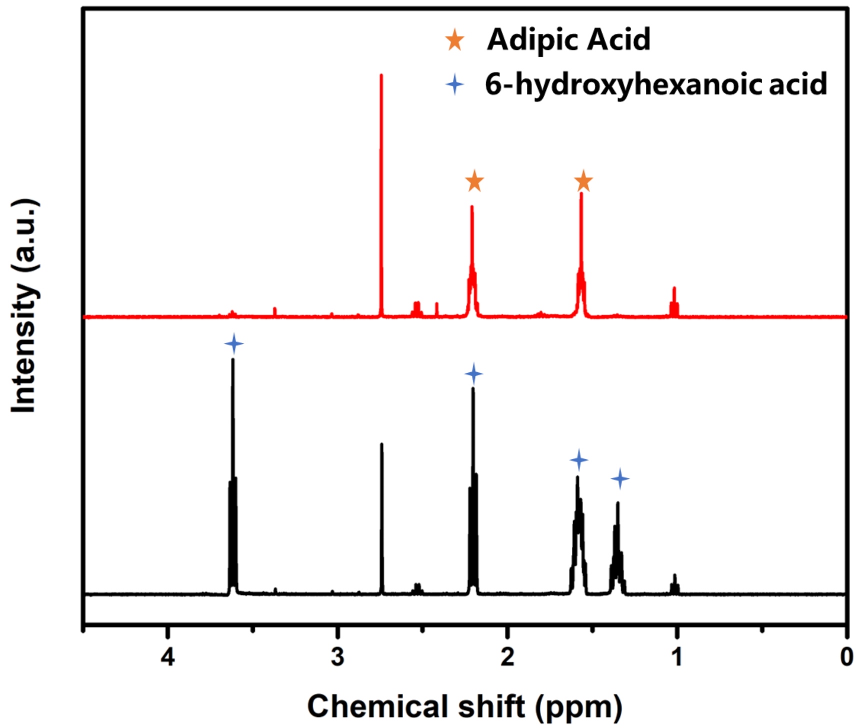

Fig. S55. ^{1}H-NMR spectrum of products over **[Ni9]-MOF** in 0.5 M KOH with 6-hydroxyhexanoic acid as the substrate.

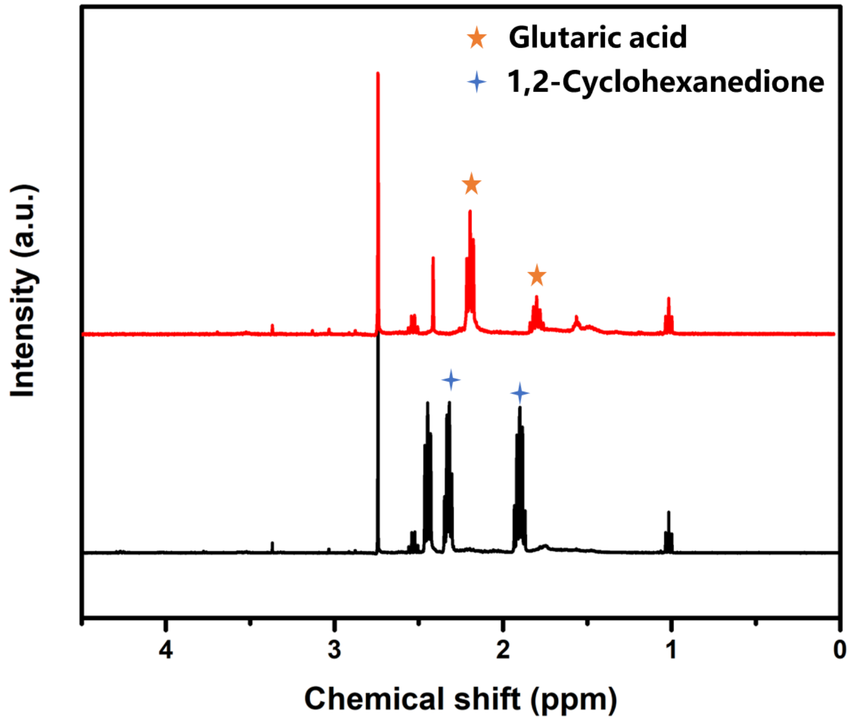

Fig. S56. ^{1}H-NMR spectrum of products over **[Ni$_9$]-MOF** in 0.5 M KOH with 1,2-cyclohexanedione as the substrate.

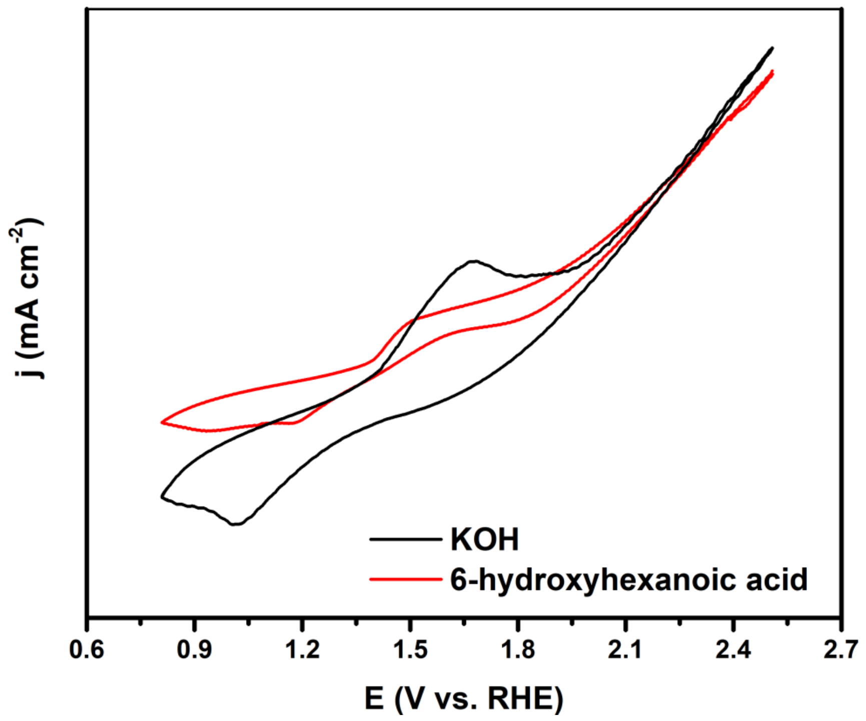

Fig. S57. CV curves of **[Ni₉]-MOF** in 0.5 M KOH electrolyte were recorded at a scan rate of 10 mV s^{-1}, both in the absence and presence of 6-hydroxyhexanoic acid.

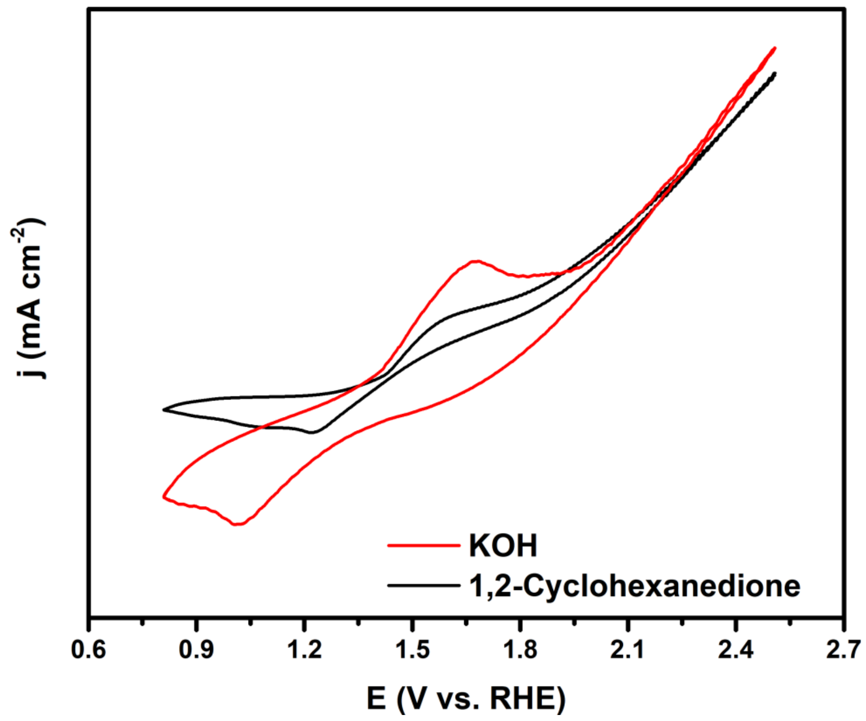

Fig. S58. CV curves of **[Ni9]-MOF** in 0.5 M KOH electrolyte were recorded at a scan rate of 10 mV s^{-1}, both in the absence and presence of 1,2-cyclohexanedione.

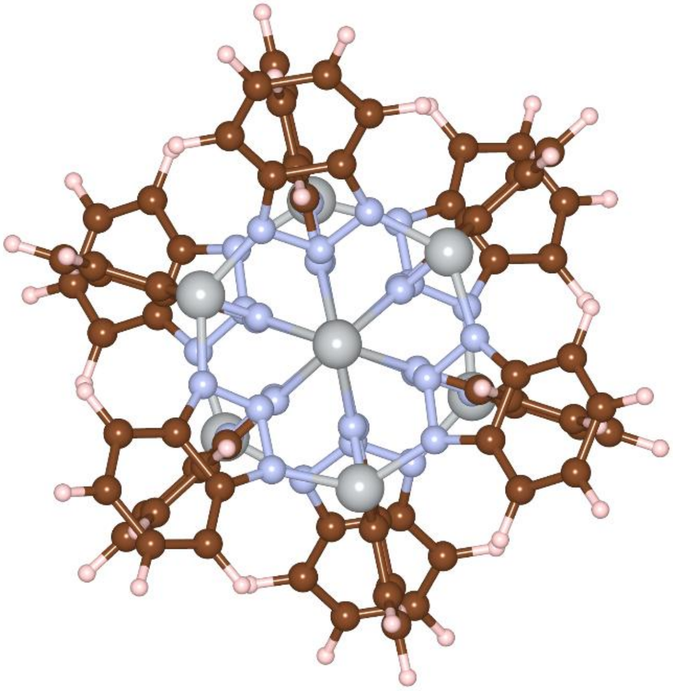

Fig. S59. The differential charge density at the **$[Ni_9]$-cluster** interface was analyzed. In the catalyst, Ni, C and N atoms were represented by gray, brown and light blue, respectively.

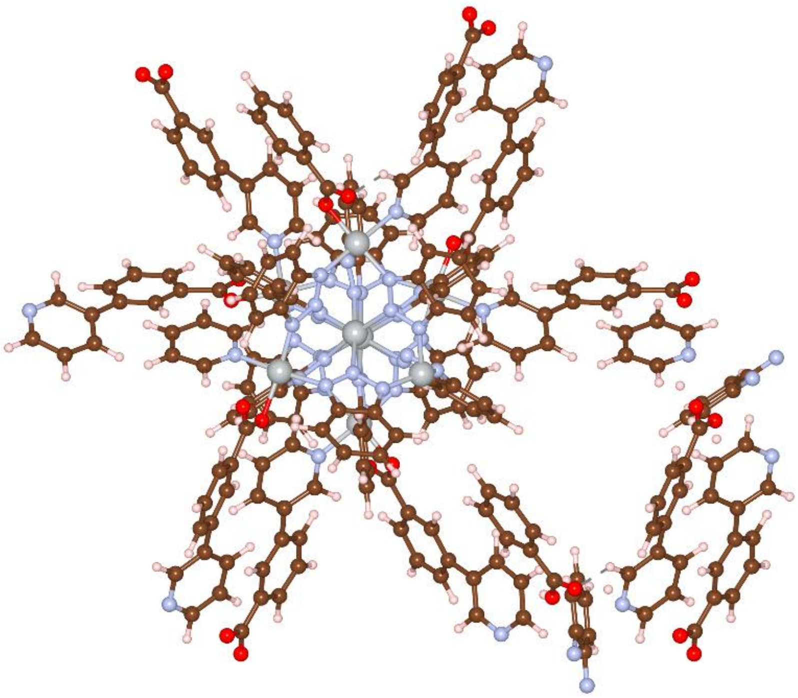

Fig. S60. The differential charge density at the **[Ni$_9$]-MOF** interface was analyzed. In the catalyst, Ni, C, N and O atoms were represented by gray, brown, light blue and red, respectively.

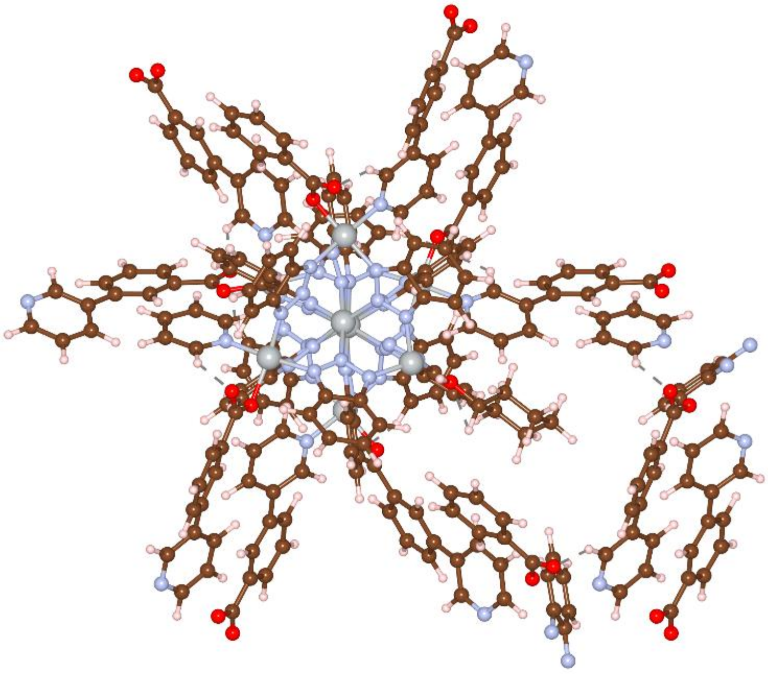

Fig. S61. The optimized adsorption configuration of cyclohexanol on the **[Ni$_9$]-MOF** surface was determined (*$C_6H_{12}O$). In the structural model, carbon (C), oxygen (O), and hydrogen (H) atoms are represented by brown, red, and pink spheres, respectively.

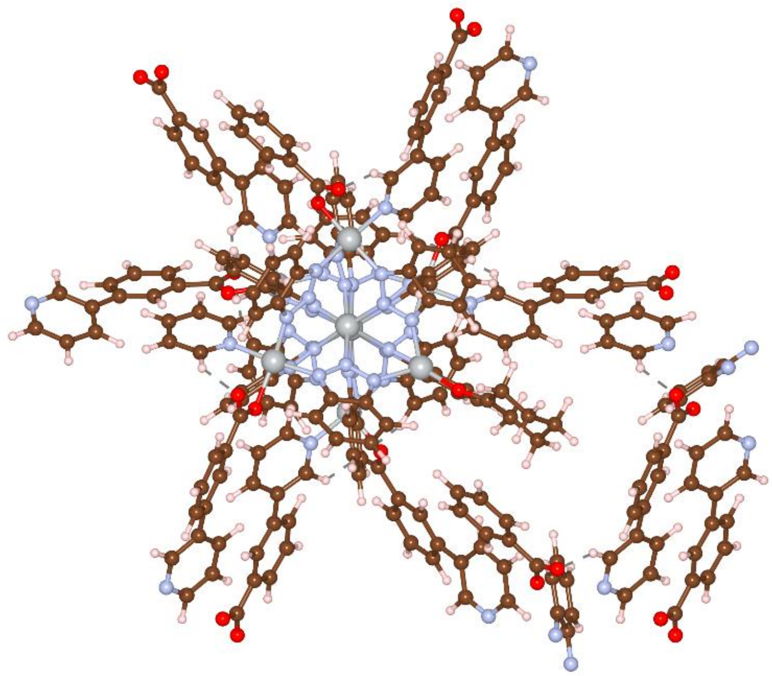

Fig. S62. The optimized adsorption configuration of cyclohexanone on the **[Ni$_9$]-MOF** surface was determined ($*C_6H_{10}O$). In the structural model, carbon (C), oxygen (O), and hydrogen (H) atoms are represented by brown, red, and pink spheres, respectively.

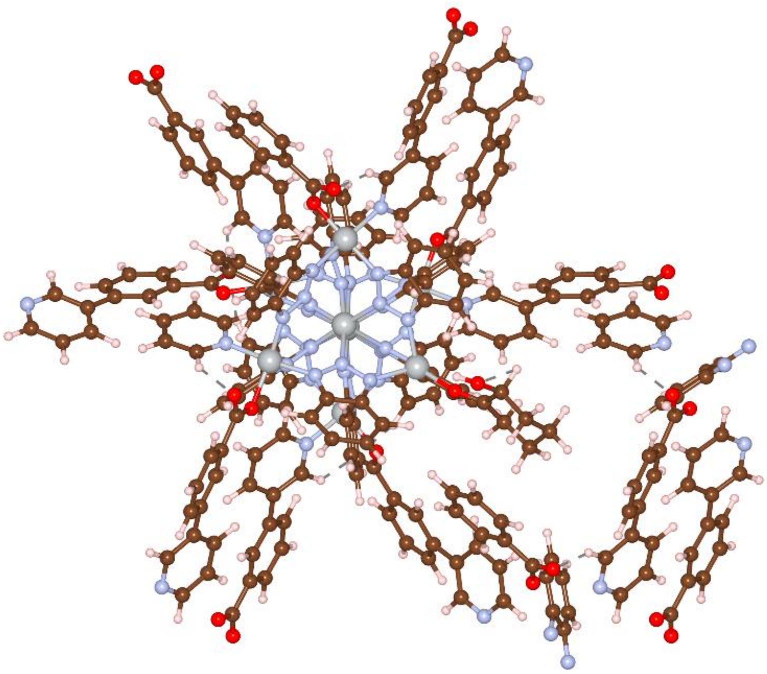

Fig. S63. The optimized adsorption configuration of 2-hydroxycyclohexanone on the **[Ni₉]-MOF** surface was determined (*$C_6H_{10}O_2$). In the structural model, carbon (C), oxygen (O), and hydrogen (H) atoms are represented by brown, red, and pink spheres, respectively.

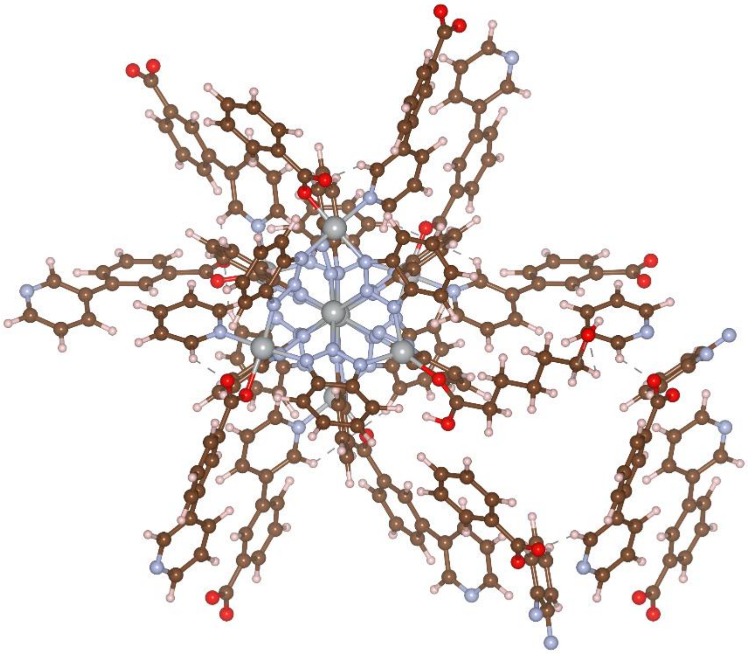

Fig. S64. The optimized adsorption configuration of 6-hydroxyhexanoic acid on the **[Ni$_9$]-MOF** surface was determined ($*C_6H_{12}O_3$). In the structural model, carbon (C), oxygen (O), and hydrogen (H) atoms are represented by brown, red, and pink spheres, respectively.

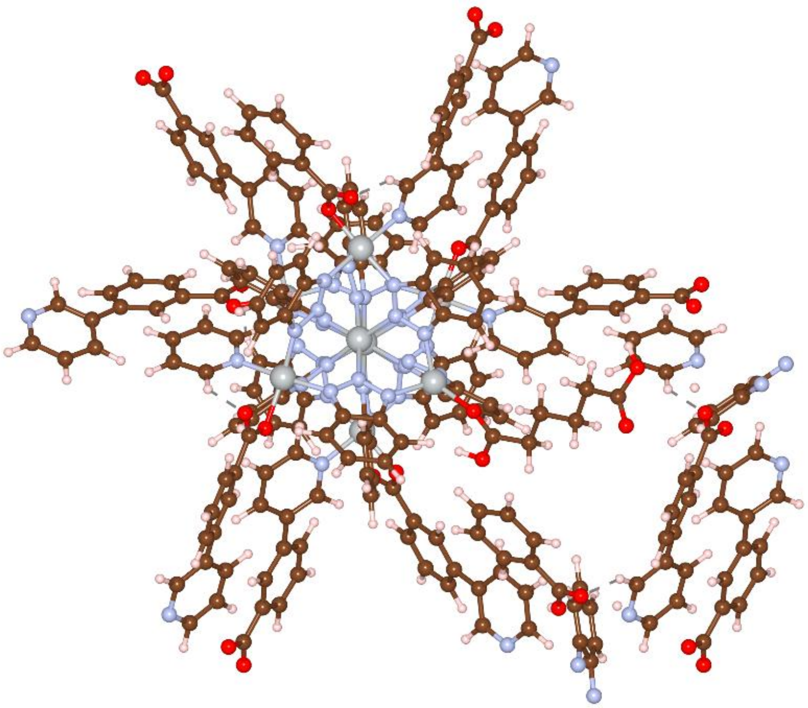

Fig. S65. The optimized adsorption configuration of adipic acid on the **[Ni$_9$]-MOF** surface was determined ($*C_6H_{10}O_4$). In the structural model, carbon (C), oxygen (O), and hydrogen (H) atoms are represented by brown, red, and pink spheres, respectively.

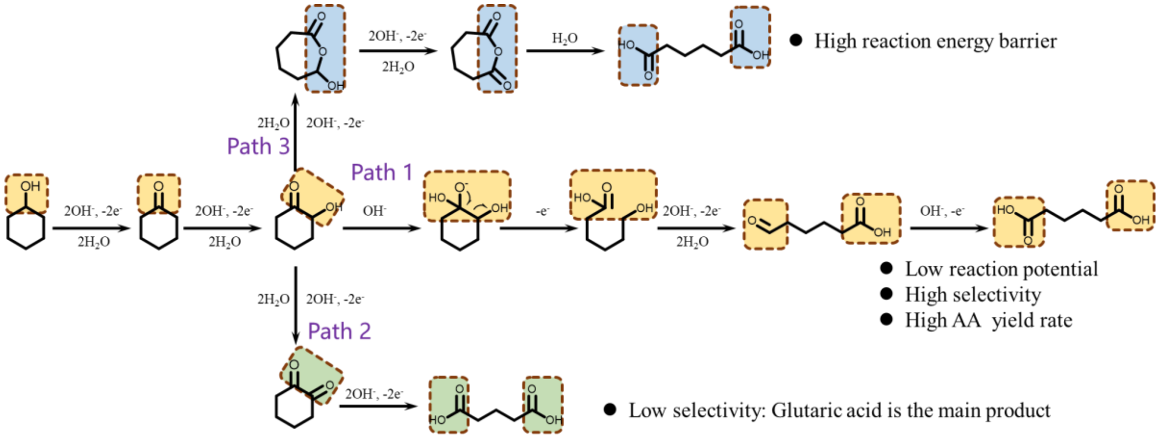

Fig. S66. Proposed reaction mechanism. Three proposed pathways for electrooxidation cyclohexanol to AA.

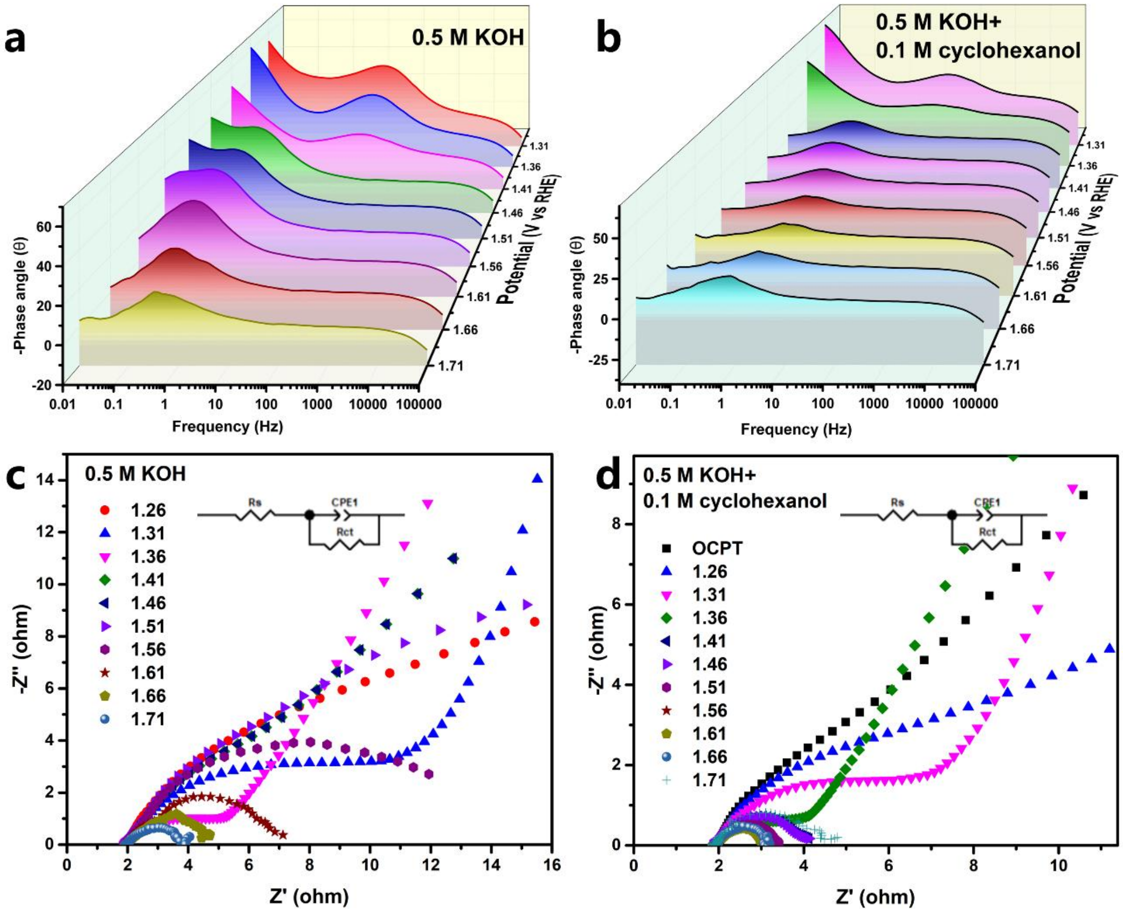

Fig. S67. Electrochemical kinetic analysis confirmed the promoting effect of **[Ni₉]-MOF** framework. Bode plots of **[Ni₉]-MOF** in 0.5 M KOH (a, c) without 0.1 M cyclohexanol and (b, d) with 0.1 M cyclohexanol.

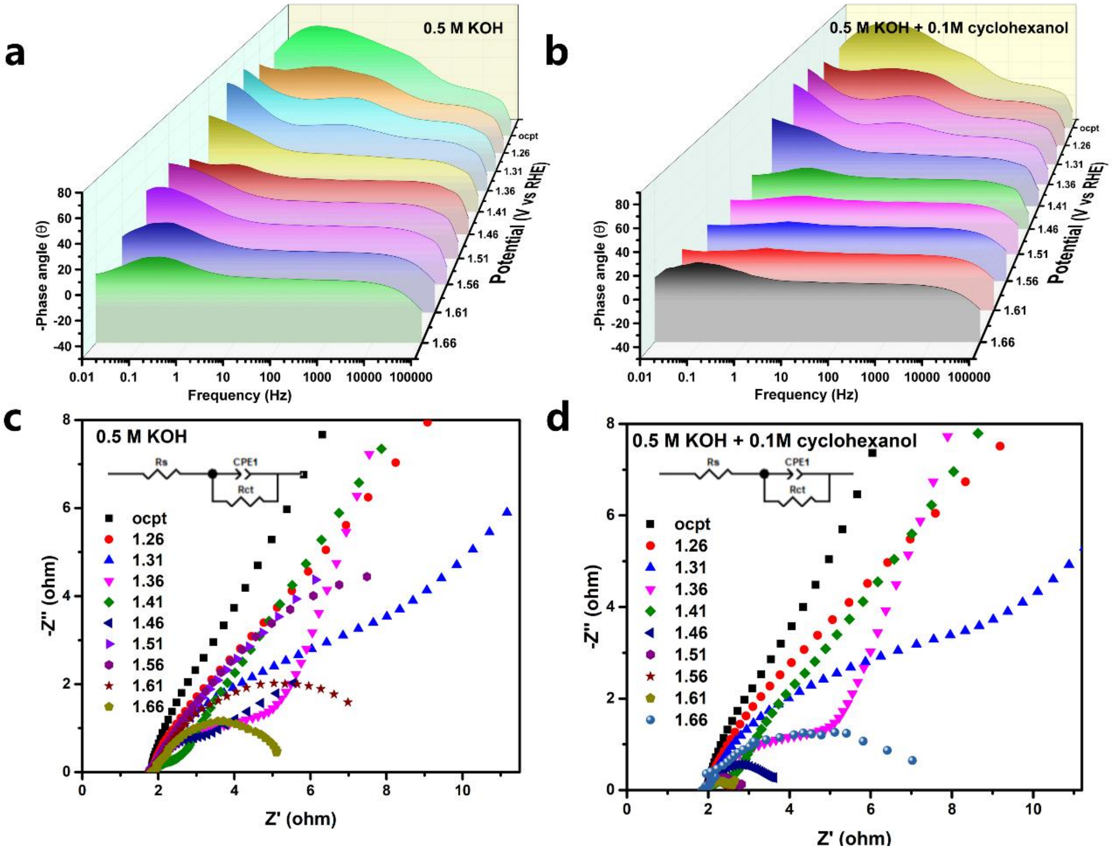

Fig. S68. Electrochemical kinetic analysis confirmed the promoting effect of **[Ni₉]-cluster** framework. Bode plots of **[Ni₉]-cluster** in 0.5 M KOH (a,c) without 0.1 M cyclohexanol and (b,d) with 0.1 M cyclohexanol.

Tables

Table S1. Crystal data and structure refinements for compound **[Ni9]-MOF**.

Identification code	**[Ni9]-MOF**
Empirical formula	$C_{48}H_{32}N_{14}Ni_3O_4$
Formula weight	1045.00
Temperature/K	120
Crystal system	Hexagonal
Space group	$P6_3$
a/Å	17.3771(5)
b/Å	17.3771(5)
c/Å	36.4536(18)
α/°	90
β/°	90
γ/°	120
Volume/Å^3	9532.9(7)
Z	6
ρ_{calc}g/cm^3	1.092
μ/mm^{-1}	0.924
F(000)	3204
Crystal size/mm^3	0.1 × 0.1 × 0.1
Radiation	CuKα (λ = 1.54184)
2Θ range for data collection/°	2.234 to 50.794
Index ranges	$-19 \le h \le 20$, $-20 \le k \le 20$, $-39 \le l \le 43$
Reflections collected	68828
Independent reflections	11162 [R_{int} = 0.0851, R_{sigma} = 0.0776]
Data/restraints/parameters	11162/440/305
Goodness-of-fit on F^2	1.038
Final R indexes [I>=2σ (I)]	R_1 = 0.1092, wR_2 = 0.2700
Final R indexes [all data]	R_1 = 0.1328, wR_2 = 0.2830
Largest diff. peak/hole / e Å^{-3}	1.22/-1.24
Flack parameter	0.32(6)
CCDC	2492643

Table S2. Crystal data and structure refinements for compound **[Ni$_9$]-cluster**.

Identification code	**[Ni$_9$]-cluster**
Empirical formula	$C_{96}H_{102}N_{48}Ni_9O_{24}$
Formula weight	2840.58
Temperature/K	120
Crystal system	Hexagonal
Space group	*R*-3
a/Å	22.1719(15)
b/Å	22.1719(15)
c/Å	24.1259(15)
α/°	90
β/°	90
γ/°	120
Volume/Å^3	10271.2(16)
Z	3.00006
ρ_{calc}g/cm^3	1.378
μ/mm^{-1}	1.286
F(000)	4374
Crystal size/mm^3	0.28 × 0.24 × 0.18
Radiation	MoKα (λ = 0.71073)
2Θ range for data collection/°	6.366 to 50.012
Index ranges	$-26 \le h \le 19$, $-16 \le k \le 23$, $-28 \le l \le 26$
Reflections collected	7233
Independent reflections	4007 [R_{int} = 0.0290, R_{sigma} = 0.0527]
Data/restraints/parameters	4007/18/270
Goodness-of-fit on F^2	1.087
Final R indexes [I>=2σ (I)]	R_1 = 0.0718, wR_2 = 0.2049
Final R indexes [all data]	R_1 = 0.0901, wR_2 = 0.2226
Largest diff. peak/hole / e Å^{-3}	1.31/-1.28
CCDC	1569801

Table S3. Crystallographic data of **[Ni_9]-MOF** after treatment in 2 M NaOH.

Conditions	As-synthesized	2M NaOH
t/h	-	2
Crystal system	hexagonal	hexagonal
Space group	$P6_3$	$P6_3$
a/Å	17.3771(5)	17.32640(10)
b/Å	17.3771(5)	17.32640(10)
c/Å	36.4536(18)	36.1394(3)
α/°	90	90
β/°	90	90
γ/°	120	120
Volume/Å^3	9532.9(7)	9395.68(13)

Table S4. Performance comparison between **[Ni_9]-MOF** and reported catalysts for cyclohexanone/cyclohexanol electrooxidation in a three-electrode system.

**Catalyst**	**Electrolyte**	**Potential / V**	**Yield rate / mmol/h**	**FE / %**	**Ref.**
**[Ni_9]-MOF**	0.5 M KOH + 0.1 M cyclohexanol	1.61	0.1239	87.6	**This work**
$Ni(OH)_2$-SDS	0.5 M KOH+0.4 M cyclohexanone	1.5	0.1958	93	(5)
Ni@Cu/CF	1 M KOH and 0.4 M cyclohexanone	1.55	0.18	88	(6)
$Cu_{0.81}Ni_{0.19}$	1 M KOH and 0.4 M cyclohexanone	1.45	0.12	92	(7)
$NiCo_2O_4/CeO_2$	1.0 M KOH + 0.1 M cyclohexanol	1.45	0.0992	87	(8)
NiV-LDH-NS	0.5 M KOH+0.4 M cyclohexanone	1.9	0.003	83	(9)
msig/ea-NiOOH-$Ni(OH)_2$/NF	1 M NaOH +0.01M cyclohexanol	1.506	-	80.3	(10)
Cu-$Ni(OH)_2$	1 M NaOH + 0.1 M cyclohexanol	1.62	0.087	84	(11)
Mo-Ni_2P@$Ni_{12}P_5$VP/NF	1.0 M KOH + 0.1 M cyclohexanol	1.53	0.0857	-	(12)
$Ni(OH)_2/TiO_2$	0.5 M KOH+0.05 M cyclohexanone	0.8	0.023	71.5	(13)

SI References

1. Kresse G, Furthmüller J. Efficiency of Ab-Initio Total Energy Calculations for Metals and Semiconductors Using A Plane-Wave Basis Set. *Comput. Mater. Sci.* 1996;**6**:15–50.
2. Blöchl PE, Jepsen O, Andersen OK. Improved Tetrahedron Method for Brillouin-Zone Integrations. *Phys. Rev. B* 1994;**49**:16223–16233.
3. Perdew JP, Chevary JA, Vosko SH *et al.* Atoms, Molecules, Solids, and Surfaces: Applications of The Generalized Gradient Approximation for Exchange and Correlation. *Phys. Rev. B* 1992;**46**:6671–6687.
4. Grimme S, Antony J, Ehrlich S *et al.* A Consistent and Accurate Ab Initio Parametrization of Density Functional Dispersion Correction (DFT-D) for the 94 Elements H-Pu. *J. Chem. Phys.* 2010;**132**:154104.
5. Li Z, Li X, Zhou H, Xu Y, Xu SM *et al.* Electrocatalytic Synthesis of Adipic Acid Coupled with H_2 Production Enhanced by A Ligand Modification Strategy. *Nat. Commun.* 2022;13:5009.
6. Jin G, Ren X, Lin C, He B, Chen P. Dual-Metal Heterogeneous Electrode Enabling Efficient Co-Electrosynthesis of Adipic Acid and Hydrogen. *J. Colloid Interface Sci.* 2025;**687**:432–438.
7. Ren X, Zhang Q, Tong Y, Zhou G *et al.* Nickel-Copper Alloying Arrays Realizing Efficient Co-Electrosynthesis of Adipic Acid and Hydrogen. *J. Energy Chem.* 2025;**101**:7–15.
8. Guo S, Wang C, Li H, Li T, Liu C *et al.* CeO_2 Modification Promotes the Oxidation Kinetics for Adipic Acid Electrosynthesis from KA Oil Oxidation at 200 mA cm^{-2}. *Angew. Chem. Int. Ed.* 2025;**64**:E202423432.
9. Liu X, Zhu YQ, Li J *et al.* Electrosynthesis of Adipic Acid with High Faradaic Efficiency within A Wide Potential Window. *Nat. Commun.* 2024;**15**:7685.
10. Jia Y, Chen Z, Gao B *et al.* Directional Electrosynthesis of Adipic Acid and Cyclohexanone by Controlling the Active Sites on NiOOH. *J. Am. Chem. Soc.* 2024;**146**:1282–1293.
11. Wang R, Kang Y, Wu J *et al.* Electrifying Adipic Acid Production: Copper-Promoted Oxidation and C−C Cleavage of Cyclohexanol. *Angew. Chem. Int. Ed.* 2022;**61**:E202214977.
12. Fan S, Yang G, Jiao Y *et al.* Doping Mo Triggers Charge Distribution Optimization and P Vacancy of Ni_2P@$Ni_{12}P_5$ Heterojunction for Industrial Electrocatalytic Production of Adipic Acid and H_2. *Adv. Mater.* 2025;**37**:2502523.
13. Zhang S, Luo L, Yang J *et al.* Photoelectrochemical Synthesis of Adipic Acid by Selective Oxidation of Cyclohexanone. *Adv. Energy Mater.* 2025;**15**:2405052.
